# Prevalence of fatty pancreas and its associations with pancreatic disease and metabolic disorders: a systematic review and meta-analysis of 124 studies

**DOI:** 10.3389/fnut.2026.1904539

**Published:** 2026-08-27

**Authors:** Wanrong Jiang, Kunpeng Wang, Lilong Zhang, Yunhao Li, Xiaoyi Zhang, Beiying Deng, Song Ding, Weixing Wang, Qiao Shi

**Affiliations:** 1 Department of Hepatobiliary and Pancreatic Surgery, Renmin Hospital of Wuhan University, Wuhan, China; 2 Central Laboratory, Renmin Hospital of Wuhan University, Wuhan, China; 3 Laboratory of General Surgery, Renmin Hospital of Wuhan University, Wuhan, China; 4 Hubei Key Laboratory of Digestive System Disease, Renmin Hospital of Wuhan University, Wuhan, China; 5 Department of Medicine, School of Clinical Medicine, Li Ka Shing Faculty of Medicine, The University of Hong Kong, Hong Kong, Hong Kong SAR, China; 6 Department of Critical Care Medicine, Zhongnan Hospital of Wuhan University, Wuhan, China; 7 Department of Gastroenterology, Renmin Hospital of Wuhan University, Wuhan, China; 8 Department of Thoracic Surgery, Renmin Hospital of Wuhan University, Wuhan, China

**Keywords:** fatty pancreas, metabolic diseases, pancreatic cancer, pancreatitis, prevalence

## Abstract

**Background:**

The prevalence and impact of fatty pancreas (FP) on pancreatic and metabolic disorders remain incompletely characterized. We synthesized current evidence on FP prevalence and its associations with pancreatic and metabolic disorders.

**Methods:**

We performed a systematic review and meta-analysis of PubMed, Embase, and Cochrane Library through October 22, 2024. Prevalence was analyzed separately in the healthy population and the overall study population, with restricted sensitivity analyses according to population selection.

**Results:**

From 3,984 records, 124 studies were included. The pooled prevalence was 30.5% among 141,416 healthy or routine-examination participants and 42.3% across all included study populations. After pancreatic disease, pancreatic surgical, and metabolic high-risk populations were excluded, the prevalence was 31.1%. In the healthy population, FP prevalence varied by region, ranging from 12.0% in Africa to 38.8% in the Region of the Americas, although estimates for some regions were based on limited country-level data. Higher prevalence was observed in older age, male sex, high-income countries, and very-high-HDI regions. FP prevalence was higher in 2020–2024 than in the preceding publication period in both the overall study population and the healthy population, while temporal meta-regression showed positive but non-significant slopes. FP was associated with increased risks of pancreatitis (OR 2.66), pancreatic cancer (OR 3.01), fatty liver (OR 4.86), metabolic syndrome (OR 3.90), obesity (OR 2.75), diabetes mellitus (OR 2.43), and hypertension (OR 2.17; all *P* < 0.001).

**Conclusion:**

FP was common in the included populations and was associated with several pancreatic and metabolic disorders. Prevalence was higher in recent publication periods, although continuous temporal trends were not statistically significant. These findings support greater clinical attention to FP, particularly among older adults and high-risk populations. Targeted evaluation for associated disorders and appropriate risk-factor management may be considered in individuals with FP.

**Systematic review registration:**

https://www.crd.york.ac.uk/PROSPERO/view/CRD42024499559, CRD42024499559.

## Graphical Abstract

**Figure:**
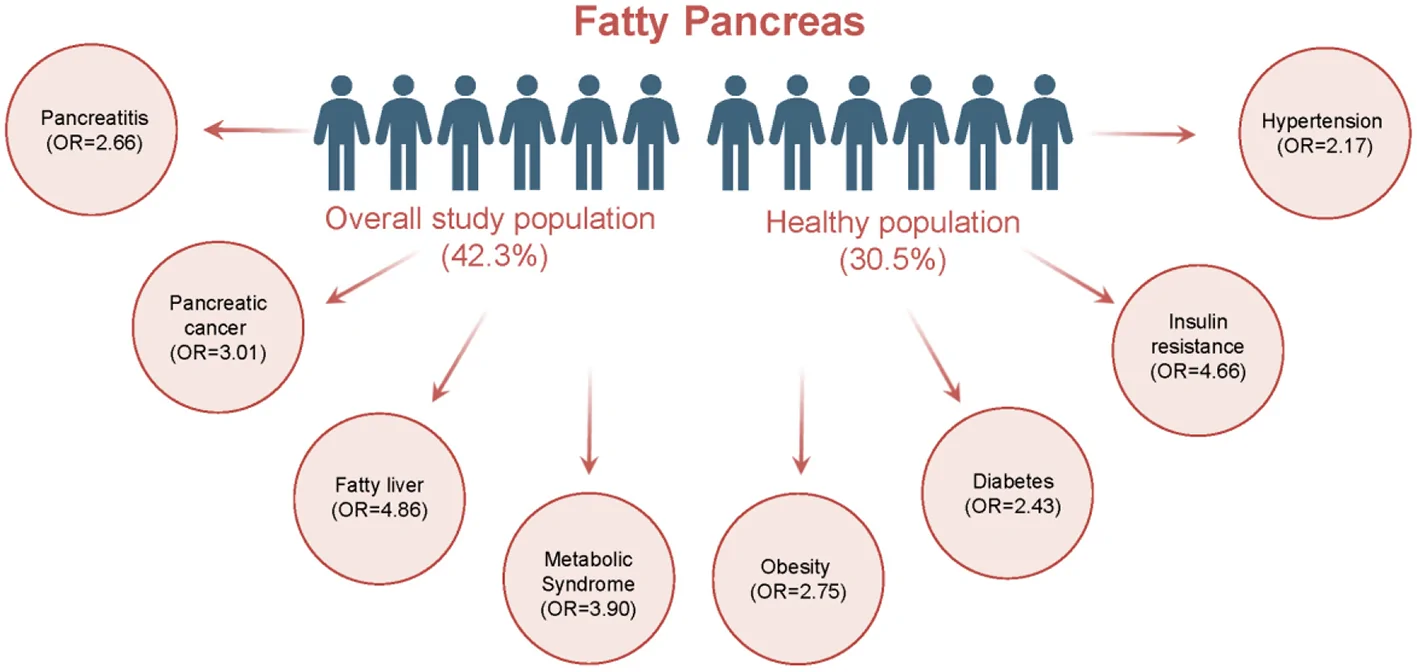
Fatty pancreas (FP) had a pooled prevalence of 30.5% in the healthy population, with higher estimates in older adults, males, and very-high-HDI settings. FP was associated with pancreatitis, pancreatic cancer, fatty liver, and metabolic disorders.

## 1 Introduction

Fatty pancreas (FP) refers to an abnormal accumulation of fat in the pancreas that exceeds the normal upper limit and is recognized as the most common form of pancreatic pathology (1). Histopathological studies have described several patterns of pancreatic fat deposition, including fatty infiltration, fatty replacement, and irregular intralobular fatty degeneration. Fatty infiltration may be related to obesity-associated visceral adiposity and local inflammation, whereas fatty replacement may be associated with acinar loss, fibrosis, or previous pancreatitis; irregular intralobular fatty degeneration may partly reflect age-related changes. These patterns may coexist and differ in their clinical implications, but they are not readily distinguished by routine imaging and are generally reported collectively as FP in epidemiological studies (2–4). In recent years, FP has garnered growing attention from researchers worldwide, with large-scale and multicenter studies from Europe and Asia increasingly reporting its prevalence in both general and high-risk populations (5–8). Across the studies included in this review, the study-level proportions of participants classified as having FP ranged from 1.21 to 92.77% (9, 10) (Supplementary Table S1), reflecting differences in population selection and diagnostic criteria. A large-scale evidence synthesis is needed to better characterize FP prevalence across regions and populations.

Recently, FP has been described as the “Pandora's box” of pancreatology (1), with research suggesting it may contribute to the development of pancreatitis and pancreatic cancer (PC) (1, 3, 11, 12). Available data also have indicated that FP impairs both the endocrine and exocrine functions of the pancreas and is associated with metabolic syndrome (MetS) and insulin resistance (IR) (5, 13, 14). More importantly, recent longitudinal evidence suggests that fatty pancreas is independently associated with subsequent development of diabetes mellitus (15). Although pancreatic fat can be assessed using imaging and histological methods, diagnostic criteria and thresholds remain heterogeneous, and FP assessment is not routinely incorporated into clinical decision-making. Its clinical implications, particularly in relation to pancreatic and metabolic diseases, also remain incompletely characterized.

Previous meta-analyses reported a pooled FP prevalence of 33% based on 11 studies and 21.11% based on 18 studies (16, 17). The review by Souza et al. (17) also examined diagnostic modalities, clinical characteristics, and related outcomes. The present review extends the available evidence by including a broader range of studies and populations and by evaluating prevalence separately in healthy or routine-examination participants and the overall study population. It further assesses the influence of population selection through restricted sensitivity analyses and examines temporal and geographical patterns, socioeconomic factors, diagnostic modalities, and associations with pancreatic and metabolic disorders.

Therefore, our systematic review and meta-analysis aimed to synthesize the available evidence on FP prevalence, temporal trends, and associations with pancreatic diseases and metabolic disorders across different regions and populations. We anticipate that the results may help and provide evidence to increase awareness among healthcare professionals and the public, promoting early diagnosis and intervention of FP to improve patient outcomes.

## 2 Methods

### 2.1 Search strategy and selection criteria

This analysis followed the Preferred Reporting Items for Systematic Reviews and Meta-Analyses (PRISMA) guidelines (18). The study protocol, registered with PROSPERO (CRD42024499559), was accessible online.

The final searches of PubMed, Embase, and the Cochrane Library were conducted on October 22, 2024, covering each database from inception. Search terms included “fatty pancreas,” “pancreatic steatosis,” “hyperechoic pancreas,” and “intrapancreatic fat deposition.” No language or regional restrictions were applied. Reference lists of relevant articles were also screened. The complete database-specific search strategies and exact queries are provided in Supplementary material. Duplicate records were removed using EndNote 20.

In addition to studies reporting FP prevalence, we included case-control studies comparing participants with FP and those without FP to investigate associations between FP and pancreatic or metabolic disorders. Studies were eligible for the prevalence analysis if they reported a defined source population, the number of FP cases, and the total number assessed, irrespective of whether prevalence was the primary study objective. Case-control studies with group sizes determined by design were used only for association analyses. Selected or augmented image datasets without a patient-level denominator were not included in the prevalence analysis. We excluded conference abstracts, editorials, reviews, commentaries, case reports, and case series. Autopsy studies were excluded because their sampling framework and pathological ascertainment differ substantially from those of studies in living populations, limiting the comparability of prevalence estimates. When multiple studies reported data from the same population or database, the most recent reports were retained to avoid duplications. This study adhered to the Declaration of Helsinki and was exempt from institutional review board approval as no confidential patient information was used.

### 2.2 Data extraction and quality assessment

Study characteristics such as the first author, publication year, country, year of sample collection, number of participants, number of FP cases, diagnostic methods and criteria for FP, age, sex, research sites, and population type were collected. The healthy population comprised participants classified in the original studies as healthy individuals or individuals undergoing routine medical examinations. All countries were classified by World Health Organization (WHO) regional settings, the World Bank's income category, and the Human Development Index (HDI). Additionally, we extracted relevant data and calculated pooled ORs for the associations of FP with fatty liver, MetS, overweight, obesity, central obesity, diabetes, IR, hypertension, hyperlipidemia, pancreatitis, and PC.

Related terms, including fatty pancreas, pancreatic steatosis, and non-alcoholic fatty pancreas disease, were grouped under FP based on the imaging or histological criteria reported in the original studies. Histopathological subtypes were not analyzed separately because they were inconsistently reported and could not be reliably inferred from imaging findings. The overall study population comprised all participants included in the prevalence analysis, including both healthy and clinically selected populations. Age-specific analyses were restricted to studies reporting FP cases and denominators within defined age groups.

The quality of the selected studies was assessed using the Joanna Briggs Institute Critical Appraisal Tool (19). To ensure transparency, studies were not excluded based on their quality ratings, which allowed all the available evidence to be considered. The literature search, screening, data extraction, and quality assessment were performed independently by three authors and cross-checked. Discrepancies were resolved by consensus or, if necessary, by consultation with the senior author.

### 2.3 Data synthesis and statistical analysis

This meta-analysis was conducted using StataMP version 18 (StataCorp LLC, College Station, TX, USA) (64-bit), with a *P*-value ≤ 0.05, set as the threshold for statistical significance. Random-effects models were used throughout (20). Pooled prevalence estimates were calculated using the DerSimonian–Laird method, whereas meta-regression models were fitted using restricted maximum likelihood. Heterogeneity was assessed using *I*^2^ and Cochran's *Q* tests (21).

The prevalence of FP was calculated using Stata's “Prevalence” module. Study-level prevalence estimates were transformed using the Freeman–Tukey double-arcsine transformation before pooling (22). Univariable and multivariable meta-regression analyses were performed using the metareg command with restricted maximum likelihood and Knapp–Hartung adjustment to explore sources of between-study heterogeneity. These analyses were conducted on the transformed prevalence scale. The predefined moderators included population, sex, age, WHO region, World Bank income level, HDI, diagnostic modality, publication year, sample size, and country. Stepwise backward selection was performed by excluding variables with *P* values greater than 0.10. Restricted sensitivity analyses excluded pancreatic disease or pancreatic surgical populations, metabolic high-risk populations, and both categories. The same model and transformation were used as in the primary prevalence analysis.

Temporal patterns were evaluated separately. Pooled prevalence was estimated across predefined publication periods using the primary meta-analysis model. Continuous temporal trends were assessed by random-effects meta-regression of raw study-level proportions, with publication year entered as a continuous moderator. Sampling variances were calculated using the binomial variance, and models were fitted using restricted maximum likelihood with Knapp–Hartung adjustment in the metafor package in R version 4.4.1 (R Foundation for Statistical Computing, Vienna, Austria). Separate models were fitted for the overall study population, the healthy population, and the sex- and HDI-specific subgroups. The regression coefficient was expressed as the annual change in prevalence in percentage points. Negative *R*^2^ estimates were reported as 0. The low–medium-HDI subgroup was not modeled because too few estimates were available. Because the estimates were obtained from different studies at irregular intervals, serial autocorrelation was not assessed. Histograms and Q-Q plots were inspected descriptively, and figures were generated using ggplot2.

The odds ratio (OR) was calculated using Stata's “Binary Outcomes” module. Sensitivity analyses were conducted by sequentially excluding individual studies to assess the results' robustness (23). Publication bias was assessed using Begg's and Egger's tests, with a *P*-value < 0.05 indicating significant bias (24, 25). If publication bias was detected, findings were confirmed using a trim-and-fill funnel plot (26).

## 3 Results

### 3.1 Characteristics of studies

**Figure 1:**
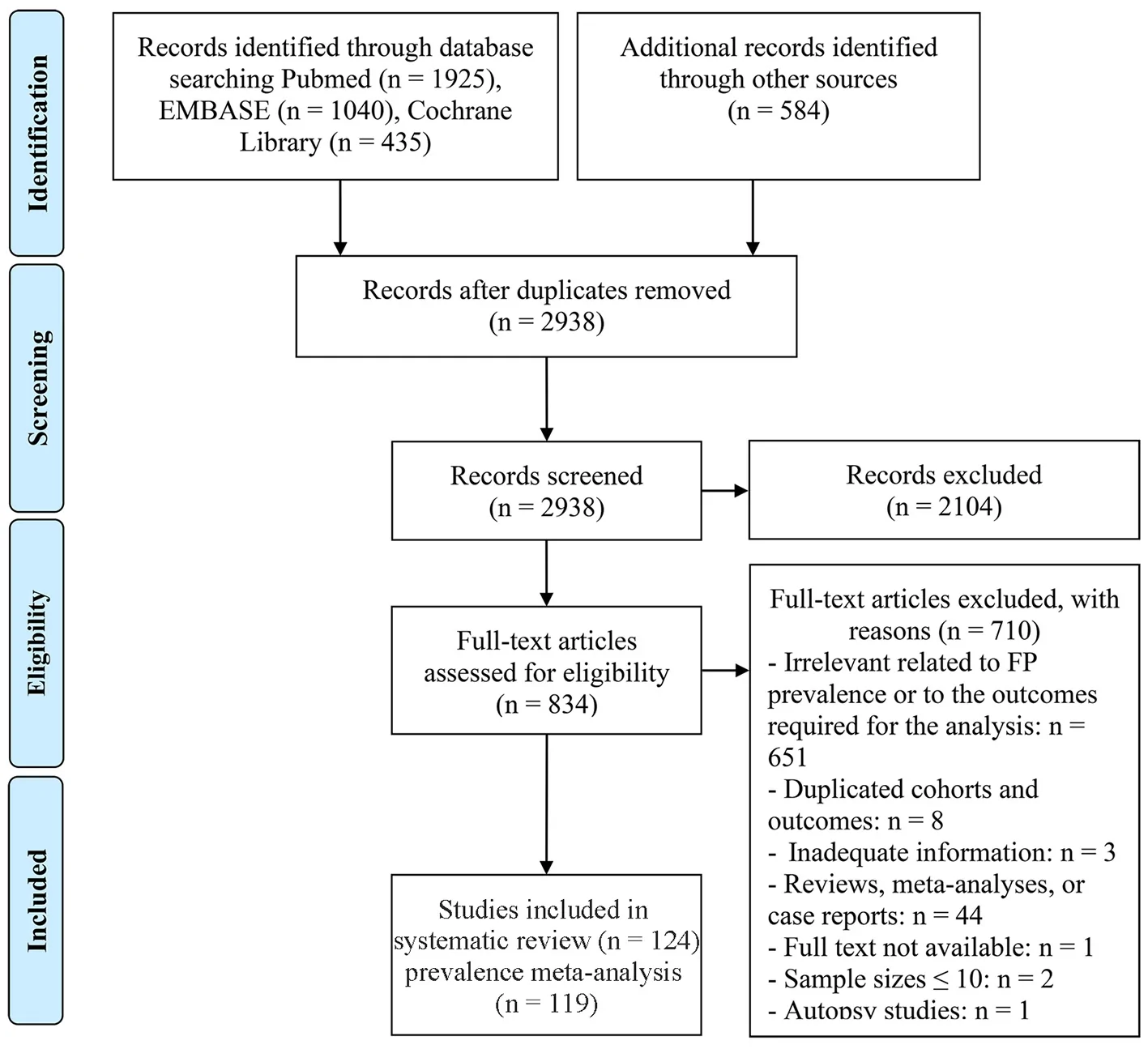
PRISMA flow diagram of study selection. FP, fatty pancreas.

The search identified 3,984 records, of which 2,938 remained after duplicate removal. Screening titles and abstracts excluded 2,104 records that did not meet the eligibility criteria. Full-text assessments were performed on the remaining 834 records, excluding 710 studies. Ultimately, 124 eligible studies from 24 countries and regions across all six WHO regions were included in the final analysis (Figure 1 and Supplementary Table S1). However, the geographical distribution of the available evidence was uneven, and many countries were not represented. Five studies were excluded from the FP prevalence calculation, including two with overlapping cohorts (Supplementary Table S2) and three case-control studies (Supplementary Table S3). The characteristics, quality assessment scores, and outcomes of the 124 studies are provided in Supplementary Tables S1–S4 and the section “References of included studies” in the Supplementary material. Sample sizes ranged from 18 to 42,599, and the study-level proportions of participants classified as having FP ranged from 1.21 to 92.77% (Supplementary Table S1).

### 3.2 Prevalence of fatty pancreas in the overall study population

**Figure 2:**
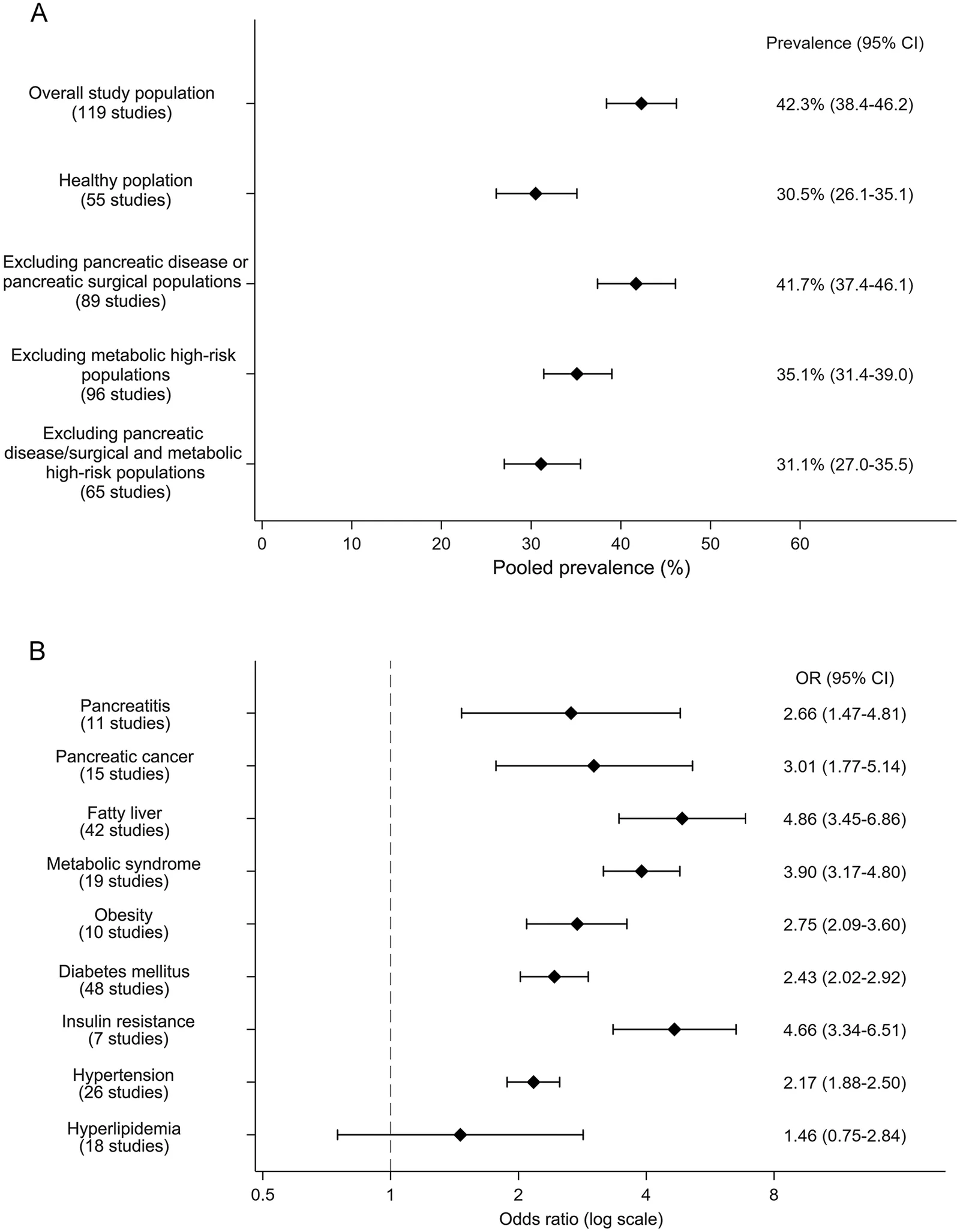
Summary forest plots of the main meta-analytic findings. **(A)** Pooled prevalence of fatty pancreas in the overall study population, healthy population, and restricted analyses. **(B)** Pooled odds ratios for pancreatic and metabolic disorders. Diamonds indicate pooled estimates and horizontal lines indicate 95% confidence intervals. CI, confidence interval; OR, odds ratio.

The pooled prevalence of FP in the overall study population, comprising both healthy and clinically selected populations, was 42.3% (95% CI: 38.4–46.2; *I*^2^ = 99.48%) across 119 studies involving 152,684 participants (Table 1, Figure 2A, and Supplementary Figure S1). Prevalence was notably higher in male patients (43.4%, 95% CI: 38.4–48.6; *I*^2^ = 99.30%) compared to female patients (36.9%, 95% CI: 32.4–41.4; *I*^2^ = 99.11%; Table 1 and Supplementary Figure S2).

**Table 1:** Prevalence of fatty pancreas in the overall study population.

Variables	Number of studies	Number of participants	Prevalence (95% CI)^‡^	*P*_heterogeneity_ (*I*^2^)	*P* value for subgroup analysis
Overall study population	119	152,684	42.3% (38.4–46.2)	< 0.001 (99.48%)	
Sex					0.057
Male	62	69,079	43.4% (38.4–48.6)	< 0.001 (99.30%)	
Female	66	68,874	36.9% (32.4–41.4)	< 0.001 (99.11%)	
WHO regions					0.048
African region	5	705	50.2% (27.6–72.7)	< 0.001 (97.11%)	
European region	42	51,829	45.2% (37.1–53.4)	< 0.001 (99.13%)	
Western Pacific region	55	96,987	41.4% (35.7–47.4)	< 0.001 (99.64%)	
Region of the Americas	11	1,739	35.9% (21.9–51.2)	< 0.001 (97.57%)	
South-East Asia region	3	1,109	34.9% (32.1–37.7)	0.408 (0)	
Eastern Mediterranean region	3	315	30.4% (20.0–41.8)	0.064 (63.57%)	
World Bank income level					0.349
High-income countries	64	81,298	44.2% (38.9–49.4)	< 0.001 (99.30%)	
Middle-income countries	55	71,386	40.1% (33.7–46.7)	< 0.001 (99.58%)	
Human Development Index					0.060
Very high human development	87	87,862	45.0% (40.1–49.9)	< 0.001 (99.32%)	
High human development	30	64,708	35.0% (28.1–42.3)	< 0.001 (99.61%)	
Low-medium human development	2	114	34.2% (18.7–51.6)	0.062 (71.24%)	
Diagnostic modality					0.311
Magnetic resonance imaging	18	45,172	45.9% (30.3–61.8)	< 0.001 (99.26%)	
Transabdominal ultrasonography	42	94,760	45.4% (38.4–52.6)	< 0.001 (99.74%)	
Histology assessment	19	3,055	42.4% (33.7–51.3)	< 0.001 (95.62%)	
Computed tomography	32	7,747	38.1% (32.8–43.6)	< 0.001 (95.70%)	
Endoscopic ultrasound	8	1,950	33.2% (22.4–44.9)	< 0.001 (96.34%)	
Population					< 0.001
Healthy population^†^	55	141,416	30.5% (26.1–35.1)	< 0.001 (99.62%)	
Children (< 18 years)	3	486	17.6% (8.9–28.4)	0.001 (86.80%)	
Pancreatic diseases					
Pancreatitis	2	433	62.2% (1.0–100.0)	< 0.001 (99.14 %)	
Pancreatic cancer	13	1,073	58.1% (39.7–75.6)	< 0.001 (96.96%)	
IPMN	3	239	48.1% (26.7–70.0)	< 0.001 (91.07%)	
PD and DP	19	2,732	45.1% (37.8–52.5)	< 0.001 (92.93%)	
Pancreatic neuroendocrine neoplasms	2	232	34.4% (8.6–66.6)	< 0.001 (96.09%)	
Extrapancreatic metabolic diseases					
Overweight or obesity	11	1,231	67.5% (56.1–78.0)	< 0.001 (93.18%)	
MASLD/formerly NAFLD	13	1,750	63.2% (48.1–77.2)	< 0.001 (97.46%)	
Diabetes mellitus	7	666	58.9% (40.4–76.2)	< 0.001 (94.45%)	

^‡^Pooled prevalence and 95% confidence interval (CI) estimated by the random-effects model.

^†^The healthy population refers to healthy individuals and those undergoing medical examinations.

PD, pancreaticoduodenectomy; DP, distal pancreatectomy; IPMN, intraductal papillary mucinous neoplasms; MASLD, metabolic dysfunction-associated steatotic liver disease; NAFLD, non-alcoholic fatty liver disease.

**Figure 3:**
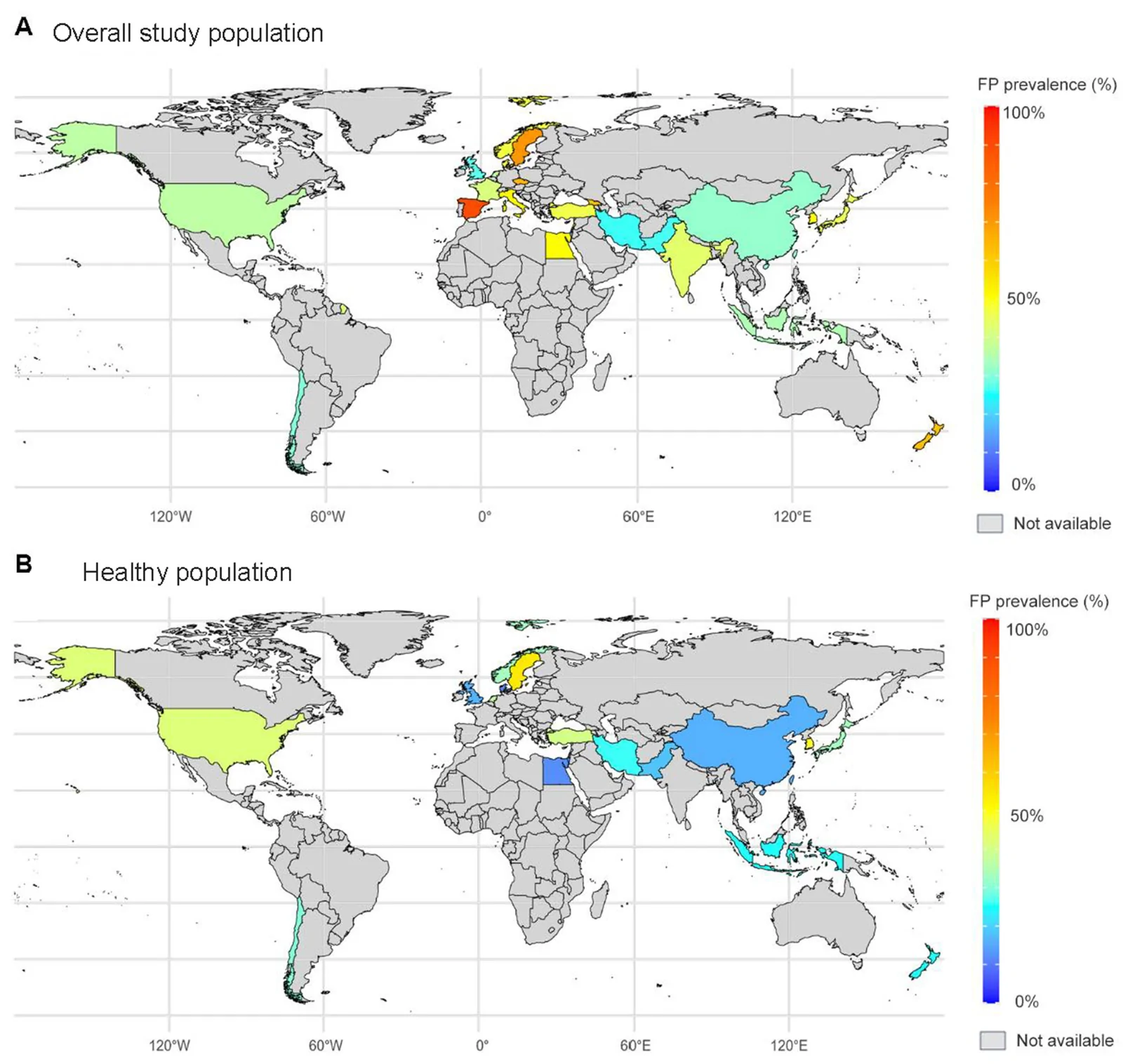
Prevalence of fatty pancreas in the overall study population **(A)** and the healthy population **(B)**.

Stratification by WHO region revealed the lowest prevalence of FP in the Eastern Mediterranean (30.4%, 95% CI: 20.0–41.8; *I*^2^ = 63.57%) and the highest in Africa (50.2%, 95% CI: 27.6–72.7; *I*^2^ = 97.11%). Prevalence estimates for Europe, the Western Pacific, the Americas, and Southeast Asia were 45.2%, 41.4%, 35.9%, and 34.9%, respectively (Table 1 and Supplementary Figure S3). The prevalence of FP across countries and territories is shown in Figure 3A and Supplementary Figure S4.

When stratified by World Bank income levels, the prevalence of FP was higher in high-income countries (44.2%, 95% CI: 38.9–49.4; *I*^2^ = 99.30%) than in middle-income countries (40.1%, 95% CI: 33.7–46.7; *I*^2^ = 99.58%; Table 1 and Supplementary Figure S5). In the meta-regression analysis, World Bank income categories were further considered as high-income, upper-middle-income, and lower-middle-income groups. No eligible studies from World Bank-defined low-income countries were identified; therefore, a pooled estimate could not be calculated for this income category. Similarly, countries with very high HDI had a higher FP prevalence (45.0%, 95% CI: 40.1–49.9; *I*^2^ = 99.32%) than those with high HDI (35.0%, 95% CI: 28.1–42.3; *I*^2^ = 99.61%). The lowest prevalence was observed in countries with low-medium HDI (34.2%, 95% CI: 18.7–51.6; *I*^2^ = 71.24%; Table 1 and Supplementary Figure S6).

Regarding the diagnostic methods, transabdominal ultrasonography (TUS) was the most commonly used for FP among the included studies (*n* = 42, 35.29%). FP prevalence estimates were 45.9% for magnetic resonance imaging (MRI), 45.4% for TUS, 42.4% for histological assessment, 38.1% for CT, and 33.2% for endoscopic ultrasonography (EUS). The subgroup difference was not statistically significant (*P* = 0.311), although substantial heterogeneity remained within each diagnostic modality (Table 1 and Supplementary Figure S7).

We also conducted a subgroup analysis based on comorbidities within the population. The healthy population comprised participants classified in the original studies as healthy individuals or individuals undergoing routine medical examinations (Table 1 and Supplementary Figure S8). Results indicated higher FP prevalence in patients with pancreatitis (62.2%, 95% CI: 1.0–100.0; *I*^2^ = 99.14%), PC (58.1%, 95% CI: 39.7–75.6; *I*^2^ = 96.96%), intraductal papillary mucinous neoplasms (48.1%, 95% CI: 26.7–70.0; *I*^2^ = 91.07%), those undergoing pancreaticoduodenectomy or distal pancreatectomy (45.1%, 95% CI: 37.8–52.5; *I*^2^ = 92.93%), as well as among individuals with overweight/obesity (67.5%, 95% CI: 56.1–78.0; *I*^2^ = 93.18%), metabolic dysfunction-associated steatotic liver disease (MASLD; formerly known as non-alcoholic fatty liver disease [NAFLD]; 63.2%, 95% CI: 48.1–77.2; *I*^2^ = 97.46%), and diabetes (58.9%, 95% CI: 40.4–76.2; *I*^2^ = 94.45%), compared to healthy population (30.5%, 95% CI: 26.1–35.1; *I*^2^ = 99.62%; Table 1 and Supplementary Figure S8).

Considerable heterogeneity was observed between these findings. We performed meta-regression analysis to explore the potential sources of this heterogeneity. In the multivariate meta-regression model, sample size and population emerged as the strongest predictors, accounting for 30.28% of the explained heterogeneity (Supplementary Table S5). In restricted sensitivity analyses, the pooled prevalence was 41.7% after excluding pancreatic disease or pancreatic surgical populations, 35.1% after excluding metabolic high-risk populations, and 31.1% after excluding both categories (Figure 2A and Supplementary Table S9). Substantial heterogeneity remained in all analyses (Supplementary Table S9).

### 3.3 Prevalence of fatty pancreas in the healthy population

We conducted subgroup analyses to examine FP prevalence in the healthy population (30.5%, 95% CI: 26.1–35.1; *I*^2^ = 99.62%). The findings revealed a higher prevalence of FP in males (36.2%, 95% CI: 30.1–42.6; *I*^2^ = 99.57%) compared to females (30.4%, 95% CI: 25.4–35.5; *I*^2^ = 99.35%; Table 2 and Supplementary Figure S9). Age-stratified prevalence data were available from three studies of healthy populations. The pooled prevalence increased from 5.8% (95% CI: 2.3–10.5; *I*^2^ = 42.14%), in individuals aged < 40 years to 17.4% at 40–49 years, 27.8% at 50–59 years, and 39.4% (95% CI: 18.0–63.1; *I*^2^ = 95.44%) at ≥60 years (Table 2 and Supplementary Figure S10).

**Table 2:** Prevalence of fatty pancreas in the healthy population.

Variable	Number of studies	Number of participants	Prevalence (95% CI)^‡^	*P*_heterogeneity_ (*I*^2^)	*P* value for subgroup analysis
Healthy population	55	141,416	30.5% (26.1–35.1)	< 0.001 (99.62%)	
Sex					0.151
Male	31	66,515	36.2% (30.1–42.6)	< 0.001 (99.57%)	
Female	33	65,535	30.4% (25.4–35.5)	< 0.001 (99.35%)	
Age at diagnosis					< 0.001
< 40 years	3	353	5.8% (2.3–10.5)	0.178 (42.14%)	
40–49 years	3	363	17.4% (13.6–21.5)	0.588 (0)	
50–59 years	3	432	27.8% (13.4–44.8)	< 0.001 (90.91%)	
≥60	3	404	39.4% (18.0–63.1)	< 0.001 (95.44%)	
WHO regions					< 0.001
Region of the Americas	4	716	38.8% (26.9–51.4)	< 0.001 (91.44%)	
European region	22	48,346	34.4% (24.4–45.2)	< 0.001 (99.23%)	
Western Pacific region	24	90,923	27.8% (21.5–34.5)	< 0.001 (99.74%)	
South-East Asia region	2	1,020	25.3% (9.4–45.6)	< 0.001 (95.01%)	
Eastern Mediterranean region	2	261	24.6% (19.4–30.1)	0.372 (0)	
African region	1	150	12.0% (7.2–17.7)	–	
World Bank income level					0.186
High-income countries	24	75,351	34.2% (27.6–41.1)	< 0.001 (99.53%)	
Middle-income countries	31	66,065	27.7% (21.1–34.8)	< 0.001 (99.64%)	
Human Development Index					< 0.001
Very high human development	39	80,302	36.4% (30.3–42.7)	< 0.001 (99.51%)	
High human development	15	61,081	17.6% (11.9–24.0)	< 0.001 (99.62%)	
Low–medium human development	1	33	18.2% (6.6–33.4)	–	
Diagnostic modality					0.001
Histology assessment	1	98	43.9% (34.2–53.8)	–	
Computed tomography	13	4,737	35.1% (30.2–40.2)	< 0.001 (91.97%)	
Transabdominal ultrasonography	24	91,077	30.1% (22.7–38.1)	< 0.001 (99.80%)	
Endoscopic ultrasound	8	1,695	28.0% (18.4–38.7)	< 0.001 (99.05%)	
Magnetic resonance imaging	9	43,809	22.7% (17.9–27.8)	< 0.001 (84.88%)	

^‡^Pooled prevalence and 95% confidence interval (CI) estimated by the random-effects model.

Data were stratified by WHO region (Table 2 and Supplementary Figure S11), showing that the Americas had the highest FP prevalence at 38.8% (95% CI: 26.9–51.4; *I*^2^ = 91.44%).

The estimate for the Region of the Americas was based on four healthy-population datasets involving 716 participants. Descriptively, three datasets were from North America, all from the United States, with 206 FP cases among 513 participants, corresponding to a crude prevalence of 40.2%. One dataset was from South America, from Chile, with 61 FP cases among 203 participants, corresponding to a crude prevalence of 30.1%. No eligible healthy-population studies were available from Central America or the Caribbean. Prevalence rates for the European, Western Pacific, Eastern Mediterranean, and South-East Asia regions were 34.4%, 27.8%, 24.6%, and 25.3%, respectively, while the African region exhibited the lowest prevalence (12.0%; 95% CI: 7.2–17.7). Regional coverage was uneven: the healthy-population estimates for Africa, South-East Asia, and the Eastern Mediterranean were based on one, two, and two studies, respectively. Figure 3B and Supplementary Figure S12 display FP prevalence across individual countries and territories in the healthy population.

The prevalence of FP in the healthy population was higher in high-income countries (34.2%, 95% CI: 27.6–41.1; *I*^2^ = 99.53%) and regions with very high HDI (36.4%, 95% CI: 30.3–42.7; *I*^2^ = 99.51%), compared to middle-income countries (27.7%, 95% CI: 21.1–34.8; *I*^2^ = 99.64%) and high-HDI regions (17.6%, 95% CI: 11.9–24.0; *I*^2^ = 99.62%; Table 2 and Supplementary Figures S13, S14). Histological assessment recorded the highest FP prevalence in the healthy population at 43.9% (95% CI: 34.2–53.8), whereas MRI yielded the lowest prevalence at 22.7% (95% CI: 17.9–27.8; *I*^2^ = 84.88%; Table 2 and Supplementary Figure S15).

In the multivariate meta-regression model, sample size and HDI emerged as primary contributors to heterogeneity, collectively accounting for 28.95% of the explained variance (Supplementary Table S6).

### 3.4 Temporal trend of fatty pancreas prevalence

Period-stratified analyses showed significant variation in pooled FP prevalence in the overall study population (*P* < 0.001). Prevalence was 57.6% in 2006–2009, 34.3% in 2010–2014, 38.7% in 2015–2019, and 44.2% in 2020–2024. Thus, prevalence increased across the three periods after 2010, whereas the higher estimate for 2006–2009 was based on only four studies involving 441 participants (Table 3 and Supplementary Figure S16). In the healthy population, prevalence was 32.6% in 2010–2014, 24.4% in 2015–2019, and 32.8% in 2020–2024. The estimate for 2020–2024 was higher than that for 2015–2019, although the overall difference across publication periods was not statistically significant (*P* = 0.139; Table 3 and Supplementary Figure S17).

**Table 3:** Temporal trend of fatty pancreas prevalence.

Variable	Number of studies	Number of participants	Prevalence (95% CI)^‡^	*P*_heterogeneity_ (*I*^2^)	*P* value for subgroup analysis
Overall study population					< 0.001
2006–2009	4	441	57.6% (51.6–63.4)	0.306 (16.95%)	
2010–2014	10	2,251	34.3% (24.0–45.3)	< 0.001 (96.29%)	
2015–2019	31	69,567	38.7% (33.3–44.3)	< 0.001 (99.30%)	
2020–2024	74	80,425	44.2% (38.3–50.2)	< 0.001 (99.51%)	
Healthy population					0.139
2010–2014	5	1,415	32.6% (20.8–45.6)	< 0.001 (95.48%)	
2015–2019	16	67,164	24.4% (19.6–29.6)	< 0.001 (99.24%)	
2020–2024	34	72,837	32.8% (25.4–40.6)	< 0.001 (99.66%)	

^‡^Pooled prevalence and 95% confidence interval (CI) estimated using the random-effects model.

**Figure 4:**
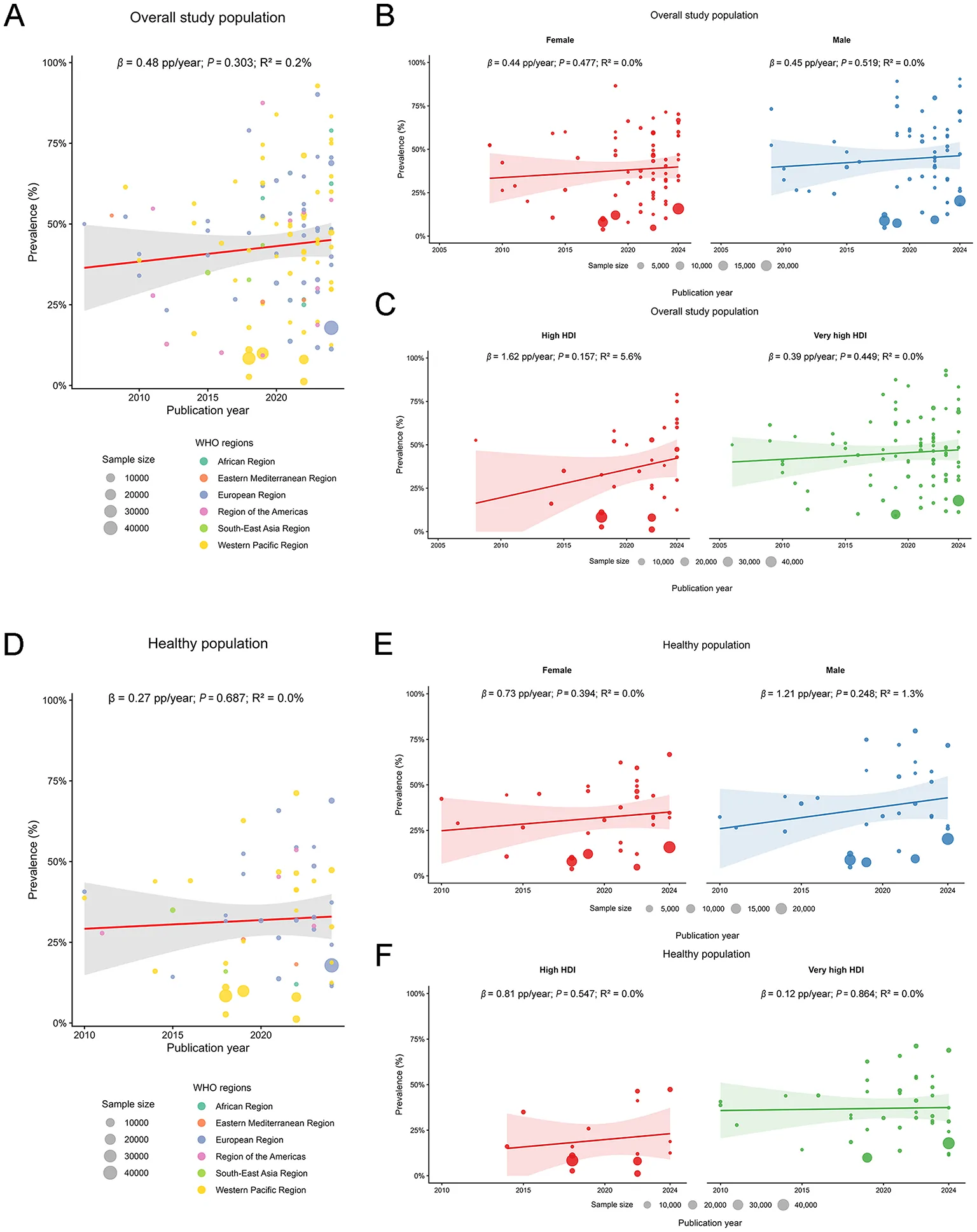
Temporal trends in fatty pancreas prevalence. **(A)** Overall study population. **(B)** Sex-specific estimates in the overall study population. **(C)** Human Development Index (HDI)-stratified estimates in the overall study population. **(D)** Healthy population. **(E)** Sex-specific estimates in the healthy population. **(F)** HDI-stratified estimates in the healthy population. Bubble size is proportional to study sample size; colors in panels A and D indicate WHO region. Solid lines and shaded bands show the fitted random-effects meta-regression and 95% confidence intervals. β denotes the annual change in prevalence, expressed as pp/year (percentage points per year), and R^2^ denotes the proportion of between-study heterogeneity explained by publication year. Low–medium-HDI subgroups were not shown because too few estimates were available for reliable meta-regression.

In a separate random-effects meta-regression of raw study-level proportions, the estimated slopes were positive but non-significant in both the overall study population (β = 0.48 percentage points/year, 95% CI: −0.44–1.40; *P* = 0.303; *R*^2^ = 0.2%) and the healthy population (β = 0.27 percentage points/year, 95% CI: −1.06–1.60; *P* = 0.687; *R*^2^ = 0.0%). Positive but non-significant slopes were also observed in the sex- and HDI-specific analyses (all *P* > 0.05; Figure 4). The low–medium-HDI subgroup was not modeled because too few estimates were available. Distribution histograms and Q-Q plots are shown in Supplementary Figures S18–S21.

### 3.5 Association of fatty pancreas with pancreatic diseases

Pancreatitis was more common in participants with FP than in those without FP (17.9 vs. 8.0%; OR: 2.66, 95% CI: 1.47–4.81, *P* < 0.001), as was PC (37.7 vs. 14.9%; OR: 3.01, 95% CI: 1.77–5.14, *P* < 0.001; Table 4, Figure 2B, and Supplementary Figures S22–S24, S26–S28). Pancreatic adenocarcinoma was also more common in participants with FP (42.6 vs. 24.6%; OR: 2.12, 95% CI: 1.18–3.81, *P* = 0.011; Supplementary Table S7 and Supplementary Figures S30–S32).

**Table 4:** Analyzing the risk of various pancreatic and metabolism-related diseases in patients with fatty pancreas.

Variable	Number of studies	Group	Number of participants	Prevalence meta		Risk meta			
				Prevalence (95% CI)^‡^	*P*^heterogeneity^ (*I*^2^)	OR (95%CI)	*P*-value	*P*^heterogeneity^ (*I*^2^)	*P*^Begg^; *P*^Egger^
Pancreatitis	11	FP	8,703	17.9% (7.9–30.6)	< 0.001 (97.90%)	2.66 (1.47–4.81)	< 0.001	< 0.001 (79.00%)	0.533; 0.059
		Non-FP	46,592	8.0% (4.3–12.7)	< 0.001 (99.07%)				
Pancreatic cancer	15	FP	8,692	37.7% (16.5–61.6)	< 0.001 (99.19%)	3.01 (1.77–5.14)	< 0.001	< 0.001 (83.04%)	0.113; 0.003
		Non-FP	36,598	14.9% (5.7–27.1)	< 0.001 (98.62%)				
Fatty liver	42	FP	9,977	61.0% (55.4–66.5)	< 0.001 (96.31%)	4.86 (3.45–6.86)	< 0.001	< 0.001 (96.24%)	0.100; 0.616
		Non-FP	52,854	25.8% (22.4–29.3)	< 0.001 (97.62%)				
Metabolic syndrome	19	FP	6,720	44.9% (37.2–52.8)	< 0.001 (96.91%)	3.90 (3.17–4.80)	< 0.001	< 0.001 (73.65%)	0.484; 0.201
		Non-FP	44,882	16.3% (13.8–19.0)	< 0.001 (94.51%)				
Obesity	10	FP	2,842	44.1% (27.2–55.6)	< 0.001 (97.70%)	2.75 (2.09–3.60)	< 0.001	< 0.001 (74.85%)	1.000; 0.238
		Non-FP	20,896	22.7% (14.6–31.8)	< 0.001 (99.25%)				
Diabetes mellitus	48	FP	17,394	26.2% (20.6–32.2)	< 0.001 (98.29%)	2.43 (2.02–2.92)	< 0.001	< 0.001 (82.70%)	0.083; 0.905
		Non-FP	88,484	13.1% (10.4–16.1)	< 0.001 (98.99%)				
Insulin resistance	7	FP	711	52.9% (33.0–72.3)	< 0.001 (96.17%)	4.66 (3.34–6.51)	< 0.001	0.336 (12.22%)	0.230; 0.141
		Non-FP	629	23.6% (10.3–40.1)	< 0.001 (94.06%)				
Hypertension	26	FP	15,164	43.4% (37.4–49.6)	< 0.001 (97.47%)	2.17 (1.88–2.50)	< 0.001	< 0.001 (78.27%)	0.597; 0.096
		Non-FP	84,671	25.2% (21.4–29.0)	< 0.001 (98.90%)				
Hyperlipidemia	18	FP	10,502	34.2% (18.7–51.6)	< 0.001 (99.38%)	1.46 (0.75–2.84)	0.268	< 0.001 (97.99%)	0.762; 0.013
		Non-FP	50,169	25.4% (3.3–58.3)	< 0.001 (99.96%)				

^‡^Pooled prevalence and 95% confidence interval (CI) estimated by the random-effects model.

FP, fatty pancreas; OR, odds ratio.

Egger's test indicated significant publication bias for PC (Table 4). Following a trim-and-fill analysis, we included the two missing studies, resulting in a pooled OR of 2.55 (95% CI: 1.49–4.37). Notably, even with the addition of these studies, the overall effect size remained consistent, suggesting that our initial findings were robust and unaffected by the absence of specific data. Sensitivity analyses conducted by systematically excluding individual studies consistently confirmed the robustness of the pooled OR (Supplementary Figures S25, S29, S33).

### 3.6 Association of fatty pancreas with metabolic diseases

By analyzing data from 62,831 patients across 42 studies, we found that the prevalence of fatty liver in FP patients was 61.0% (95% CI: 55.4–66.5), significantly higher than the 25.8% (95% CI: 22.4–29.3) observed in non-FP patients (OR: 4.86, 95% CI: 3.45–6.86, *P* < 0.001; Table 4 and Supplementary Figures S34–S36). Among these studies, 15 studies showed a significantly higher prevalence of study-defined NAFLD, now generally termed MASLD, in FP patients compared to non-FP patients (65.0 vs. 29.6%; OR: 4.95, 95% CI: 2.67–9.19, *P* < 0.001; Supplementary Table S7 and Supplementary Figures S38–S40).

Furthermore, a pooled analysis of 19 studies found that the odds of MetS were 3.90-fold higher in FP patients than in non-FP patients (44.9 vs. 16.3%; OR: 3.90, 95% CI: 3.17–4.80, *P* < 0.001; Table 4 and Supplementary Figures S42–S44). We also evaluated the associations of FP with overweight, obesity, and central obesity, with diagnostic criteria provided in Supplementary Table S8. The results revealed that the prevalence of overweight (41.3 vs. 31.6%; OR: 1.89, 95% CI: 1.23–2.92, *P* = 0.004), obesity (44.1 vs. 22.7%; OR: 2.75, 95% CI: 2.09–3.60, *P* < 0.001), and central obesity (76.1 vs. 40.4%; OR: 4.88, 95% CI: 3.51–6.77, *P* < 0.001) were significantly higher in FP patients compared to non-FP patients (Table 4, Supplementary Table S7 and Supplementary Figures S46–S48, S50–S52, S54–S56).

This pooled analysis of 17,394 FP patients showed a diabetes prevalence of 26.2% (95% CI: 20.6%−32.2%), significantly higher than the 13.1% (95% CI: 10.4–16.1) prevalence observed in 88,484 non-FP patients (OR: 2.43, 95% CI: 2.02–2.92, *P* < 0.001; Table 4 and Supplementary Figures S58–S60). Among these, 12 studies focused on T2DM prevalence, revealing a markedly higher rate in FP patients (35.2 vs. 13.4%; OR: 3.81, 95% CI: 2.11–6.87, *P* < 0.001; Supplementary Table S7 and Supplementary Figures S62–S64). Additionally, the odds of IR were higher in participants with FP than in those without FP (52.9 vs. 23.6%; OR: 4.66, 95% CI: 3.34–6.51, *P* < 0.001; Table 4 and Supplementary Figures S66–S68). In a meta-analysis of 26 studies (99,835 participants), FP patients had a significantly higher rate of hypertension (43.4 vs. 25.2%; OR: 2.17, 95% CI: 1.88–2.50, *P* < 0.001; Table 4 and Supplementary Figures S70–S72). No significant difference in hyperlipidemia prevalence was found between FP and non-FP groups (34.2 vs. 25.4%; OR: 1.46, 95% CI: 0.75–2.84, *P* = 0.268; Table 4 and Supplementary Figures S74–S76). The principal pooled associations are summarized in Figure 2B.

Egger's test indicated publication bias for hyperlipidemia; however, the trim-and-fill method showed no missing studies and no change in the OR after correction. Sensitivity analyses were conducted for all evaluated outcomes (Supplementary Figures S37, S41, S45, S49, S53, S57, S61, S65, S69, S73, S77). Most pooled estimates remained stable after sequential study exclusion. However, the association with overweight was sensitive to study omission; after exclusion of Berger et al. (27) the pooled OR became non-significant, indicating limited robustness of this association (Supplementary Figure S49).

## 4 Discussion

This review included 124 studies from all six WHO regions. The pooled prevalence of FP was 42.3% in the overall study population and 30.5% in healthy populations. Because the overall estimate included clinically selected cohorts, it should not be interpreted as an age-standardized prevalence for the general population. After pancreatic disease, pancreatic surgical, and metabolic high-risk populations were excluded, the pooled prevalence decreased to 31.1%. FP prevalence was higher in 2020–2024 than in the preceding publication period, although continuous temporal trends were not statistically significant. Age-stratified estimates increased with age, and higher pooled estimates were observed in males and very-high-HDI settings. FP was also associated with pancreatitis, PC, fatty liver, MetS, obesity, diabetes, and hypertension.

Period-stratified analyses showed progressively higher FP prevalence after 2010 in the overall study population, with the highest estimate observed in 2020–2024. A similar pattern was found in the healthy population, in which prevalence was higher in 2020–2024 than in 2015–2019. Continuous meta-regression also yielded positive slopes, although these trends did not reach statistical significance. Together, these findings suggest that FP has been more frequently detected in recent publication periods, but do not establish a continuous linear increase over time. Greater recognition of FP, wider availability of abdominal imaging, and advances in imaging techniques may have contributed to this pattern. Recent multicenter and biobank-based studies have similarly reported frequent pancreatic steatosis and associations with metabolic risk (6, 8, 28). However, diagnostic modality was not significantly associated with prevalence in the subgroup analysis, indicating that changes in imaging use alone are unlikely to explain the temporal pattern. The estimate for 2006–2009 was less precise because it was derived from only four small studies. In healthy populations, FP prevalence increased with age and reached 39.4% among individuals aged ≥60 years, although age-stratified data were available from only three studies. Earlier autopsy studies likewise reported greater pancreatic lipomatosis with advancing age (29, 30). Prevalence was also slightly higher in males than in females, but the subgroup difference was not statistically significant.

In healthy populations, FP prevalence was higher in very-high-HDI settings, whereas the difference between high- and middle-income countries was not statistically significant. This pattern may reflect differences in metabolic risk, population age, and access to health examinations and imaging. However, no eligible studies were available from low-income countries, and regional coverage was uneven. Africa, South-East Asia, and the Eastern Mediterranean were represented by few studies, while the Region of the Americas was represented by datasets from the United States and Chile. The regional estimates therefore summarize the available evidence rather than population-weighted prevalence across all countries.

CT, MRI, ultrasonography, and histological assessment were used to evaluate FP, with variation in diagnostic criteria and thresholds. Prevalence did not differ significantly by modality in the overall study population, but this does not imply equivalent diagnostic performance. Histological studies generally used surgically selected specimens and may detect microscopic fat not apparent on imaging, whereas imaging studies included broader populations. Pancreatic hyperechogenicity is also not specific for fat because fibrosis may produce a similar appearance (4, 31). Quantitative CT and MRI may provide more reproducible assessment, but validated thresholds remain lacking. MRI-PDFF thresholds such as >6.2% have been used, although population- and modality-specific validation is required (16, 32, 33). Emerging quantitative and deep learning-based ultrasound methods may further improve reproducibility (34, 35).

FP was associated with both pancreatitis and PC. Pancreatic fat may contribute to pancreatitis through local lipolysis and the release of unsaturated fatty acids, which can promote acinar injury and necrosis (36–38). Clinical studies have also linked pancreatic steatosis with pancreatitis severity, pancreatic cancer and post-ERCP pancreatitis (3, 37, 39, 40). A recent whole-pancreas histopathological study in elderly cadavers distinguished fatty infiltration, fatty replacement, and irregular intralobular fatty degeneration as different patterns of pancreatic fat deposition. Fatty infiltration was more often accompanied by inflammation, fibrosis, and acinar changes, whereas irregular intralobular fatty degeneration appeared to represent a less inflammatory, age-related pattern (2). Because most included studies did not distinguish these patterns, the pooled estimate reflects the association between overall FP and pancreatitis rather than that of any specific histopathological subtype.

The association between FP and PC may involve adipose-tissue inflammation, metabolic dysfunction, and pancreatic cell injury (41, 42). Fatty infiltration and fatty replacement may contribute through partly different pathways, including obesity- and insulin-related mechanisms, acinar-to-ductal metaplasia, and acinar-to-adipocyte transdifferentiation (43–45). These mechanisms remain difficult to evaluate in imaging-based epidemiological studies. Pancreatic hyperechogenicity has also been associated with post-operative pancreatic fistula, suggesting possible perioperative relevance (46).

In the prevalence analysis, FP was more common in populations with MASLD than in healthy population. Conversely, fatty liver was more common among individuals with FP than among those without FP (OR 4.86). This pattern is consistent with the reported bidirectional association between pancreatic and hepatic steatosis, although temporal sequence and causality remain uncertain (47). Shared mechanisms may include insulin resistance, altered lipid trafficking, and lipotoxicity, which can promote ectopic fat accumulation in both the liver and pancreas. Fetuin-A-mediated inflammatory signaling may further contribute to metabolic crosstalk between these organs (48, 49).

FP was also associated with MetS and several of its components. Prevalence was higher in populations with overweight or obesity and diabetes, and individuals with FP had higher odds of MetS, obesity, diabetes, and hypertension. These associations are consistent with the clustered metabolic and inflammatory features of MetS (4, 50). Clinical evidence links FP with insulin resistance and impaired glucose metabolism, including higher frequencies of prediabetes and diabetes among patients with NASH (14, 51, 52). Experimental and mechanistic studies suggest that pancreatic fat may contribute to oxidative stress, β-cell lipotoxicity, and lipoapoptosis (53–55). The association with hyperlipidemia was not statistically significant, indicating that circulating lipid abnormalities alone do not account for FP. Associations with obstructive sleep apnoea and gut microbiota-derived metabolites further suggest that FP may reflect broader metabolic dysfunction (56, 57).

This study has several limitations. The included studies covered 24 countries and regions, with no eligible data from low-income countries and uneven regional representation. Age-specific data were available from only three healthy-population studies, precluding age-standardized estimates. Definitions of healthy populations and clinical selection criteria varied; although restricted analyses reduced the pooled prevalence, heterogeneity remained substantial. Alcohol exposure and criteria for a non-alcoholic phenotype were inconsistently reported, so the pooled estimates reflect pancreatic fat accumulation rather than a uniformly defined non-alcoholic condition. Diagnostic modalities, thresholds, and histopathological reporting also varied, preventing direct comparison of diagnostic performance or subtype-specific estimates. Publication year was used as a study-level proxy for time. The temporal analysis comprised independent study-level estimates reported at irregular intervals rather than repeated observations from a single population; therefore, conventional time-series autocorrelation tests were not performed, and the findings should be interpreted as between-study temporal patterns rather than within-population longitudinal changes. Finally, most studies were cross-sectional or case-control, limiting causal inference.

Despite these limitations, this study provides an updated and broad synthesis of FP prevalence and its associations with pancreatic and metabolic disorders. The findings support greater awareness of FP among primary care physicians, specialists, and policymakers. Future studies should establish standardized diagnostic criteria and report age-stratified prevalence data to improve comparability across populations. Quantitative imaging and artificial intelligence-assisted approaches may improve the reproducibility of FP assessment, but require external validation and standardized thresholds. Multicenter longitudinal cohorts are needed to clarify temporal and potentially causal relationships, while population-based studies in low-income and low-HDI settings would improve the geographical representativeness of the evidence.

## 5 Conclusion

This study highlights that FP was common in the included populations and was associated with pancreatic and metabolic disorders. Prevalence was higher in recent publication periods, although continuous temporal trends were not statistically significant. These findings support greater clinical attention to FP, particularly among older adults and high-risk populations. Targeted evaluation for associated pancreatic and metabolic disorders and appropriate risk-factor management may be considered in individuals with FP.

## Data Availability

The original contributions presented in the study are included in the article/Supplementary material, further inquiries can be directed to the corresponding authors.
