# Supplementary material for: Prevalence of fatty pancreas and its associations with pancreatic disease and metabolic disorders: a systematic review and meta-analysis of 124 studies
Source: Front Nutr. 2026 Aug 27;13:1904539. doi: 10.3389/fnut.2026.1904539 (PMC13561114; doi:10.3389/fnut.2026.1904539)
Supplement: Supplementary file 1 [file Supplementary_File_1.docx]

**Supplementary materials**

**Content list:**

| 1. Supp.1 Search strategy……………………………………………………………………………………………………………………………... | Page 5 |
| --- | --- |
| 2. Supp. Table 1 Reported prevalence of fatty pancreas in six WHO regions……………………………………………………………………… | Page 7 |
| 3. Supp. Table 2 Bibliography of Repeat Populations…………………………………………………………………………………………...…… | Page 33 |
| 4. Supp. Table 3 The case-control study of fatty versus non-fatty pancreas………………………………………………………………………….. | Page 34 |
| 5. Supp. Table 4 Quality assessment and study outcomes…….……………………………………………………………………………………… | Page 35 |
| 6. Supp. Table 5 Meta-regression analyses of potential sources of heterogeneity in fatty pancreas prevalence in the overall study population…… | Page 44 |
| 7. Supp. Table 6 Meta-regression analyses of potential sources of heterogeneity in fatty pancreas prevalence in the healthy population………… | Page 52 |
| 8. Supp. Table 7 Analyzing the risk of various pancreatic and metabolism-related diseases in patients with fatty pancreas………………………... | Page 58 |
| 9. Supp. Table 8 Diagnostic criteria for overweight, obesity, and central obesity……………………………………………………………………. | Page 59 |
| 10. Supp. Table 9 Restricted sensitivity analyses of fatty pancreas prevalence according to population selection………………. ………………. … 11. References of included studies…………………………………………………………………………………………………………………... | Page 61  Page 62 |
| 12. Supp. Figure 1 Forest plot of fatty pancreas prevalence in the overall study population…………………………..…………………...……….. | Page 71 |
| 13. Supp. Figure 2 Forest plot of fatty pancreas prevalence in the overall study population by sex…………………………………………...……. | Page 74 |
| 14. Supp. Figure 3 Forest plot of fatty pancreas prevalence in the overall study population by WHO region…………………………………….... | Page 78 |
| 15. Supp. Figure 4 Forest plot of fatty pancreas prevalence in the overall study population across different countries……………………..……… | Page 82 |
| 16. Supp. Figure 5 Forest plot of fatty pancreas prevalence in the overall study population by World Bank income levels…………………………. | Page 88 |
| 17. Supp. Figure 6 Forest plot of fatty pancreas prevalence in the overall study population by Human Development Index levels………...……… | Page 91 |
| 18. Supp. Figure 7 Forest plot of fatty pancreas prevalence in the overall study population by diagnostic modalities………………..……...……… | Page 94 |
| 19. Supp. Figure 8 Forest plot of fatty pancreas prevalence across different populations………………….…………………………………………. | Page 98 |
| 20. Supp. Figure 9 Forest plot of fatty pancreas prevalence in the healthy population by sex………………………...……………………………… | Page 103 |
| 21. Supp. Figure 10 Forest plot of fatty pancreas prevalence in the healthy population by age at diagnosis…………………………….…………… | Page 105 |
| 22. Supp. Figure 11 Forest plot of fatty pancreas prevalence in the healthy population by WHO region……………………..…………..…….……. | Page 106 |
| 23. Supp. Figure 12 Forest plot of fatty pancreas prevalence in the healthy population across different countries……………..……………….…… | Page 108 |
| 24. Supp. Figure 13 Forest plot of fatty pancreas prevalence in the healthy population by World Bank income levels…………………………….... | Page 112 |
| 25. Supp. Figure 14 Forest plot of fatty pancreas prevalence in the healthy population by Human Development Index levels……………….……... | Page 114 |
| 26. Supp. Figure 15 Forest plot of fatty pancreas prevalence in the healthy population by diagnostic modalities………………………….…….…... | Page 116 |
| 27. Supp. Figure 16 Forest plot of fatty pancreas prevalence in the overall study population by publication period………………….……………. | Page 118 |
| 28. Supp. Figure 17 Forest plot of fatty pancreas prevalence in the healthy population by publication period……………………………………... | Page 122 |
| 29. Supp. Figure 18 Normality histogram for the FP prevalence in the overall study population…………………………………………………… | Page 124 |
| 30. Supp. Figure 19 Normality Q-Q plot for the FP prevalence in the overall study population……………………………………………………… | Page 125 |
| 31. Supp. Figure 20 Normality histogram for the FP prevalence in healthy population……………………………………………………….……… | Page 126 |
| 32. Supp. Figure 21 Normality Q-Q plot for the FP prevalence in healthy population……………………………………………………….……… | Page 127 |
| 33. Supp. Figure 22 Forest plot of pancreatitis prevalence in fatty pancreas…………………………..…….…....…………..………………...…… | Page 128 |
| 34. Supp. Figure 23 Forest plot of pancreatitis prevalence in the population without fatty pancreas……………….…….….……………..………… | Page 128 |
| 35. Supp. Figure 24 Forest plot of the risk of pancreatitis in patients with fatty pancreas………………………..….………..……………………… | Page 129 |
| 36. Supp. Figure 25 Sensitivity analysis of the risk of pancreatitis in patients with fatty pancreas….…….….….….…………..……………………. | Page 129 |
| 37. Supp. Figure 26 Forest plot of pancreatic cancer in fatty pancreas………………..……………….….…….….…...………..…………………… | Page 130 |
| 38. Supp. Figure 27 Forest plot of pancreatic cancer prevalence in the population without fatty pancreas………………………..…….….………... | Page 131 |
| 39. Supp. Figure 28 Forest plot of the risk of pancreatic cancer in patients with fatty pancreas……………..…….……...…………………….……. | Page 132 |
| 40. Supp. Figure 29 Sensitivity analysis of the risk of pancreatic cancer in patients with fatty pancreas……..……….….………………………….. | Page 133 |
| 41. Supp. Figure 30 Forest plot of pancreatic adenocarcinoma in fatty pancreas……..………………..….…….…..……………….……………….. | Page 134 |
| 42. Supp. Figure 31 Forest plot of pancreatic adenocarcinoma prevalence in the population without fatty pancreas…………..….….……………… | Page 134 |
| 43. Supp. Figure 32 Forest plot of the risk of pancreatic adenocarcinoma in patients with fatty pancreas.…….….………….…………...…..……... | Page 135 |
| 44. Supp. Figure 33 Sensitivity analysis of the risk of pancreatic adenocarcinoma in patients with fatty pancreas…….….….……...………….…… | Page 136 |
| 45. Supp. Figure 34 Forest plot of fatty liver prevalence in fatty pancreas…………….……………………………………………………………… | Page 136 |
| 46. Supp. Figure 35 Forest plot of fatty liver prevalence in the population without fatty pancreas…………………………………………………… | Page 138 |
| 47. Supp. Figure 36 Forest plot of the risk of fatty liver in patients with fatty pancreas…...…...…………………………………………………….. | Page 140 |
| 48. Supp. Figure 37 Sensitivity analysis of the risk of fatty liver in patients with fatty pancreas…..…………………………………………………. | Page 141 |
| 49. Supp. Figure 38 Forest plot of MASLD/formerly NAFLD prevalence in fatty pancreas………………………..…….….... .….. .….... ….... …. | Page 142 |
| 50. Supp. Figure 39 Forest plot of nonalcoholic fatty liver disease prevalence in the population without fatty pancreas………..……….………….. | Page 143 |
| 51. Supp. Figure 40 Forest plot of the risk of nonalcoholic fatty liver disease in patients with fatty pancreas………….…………..…………..……. | Page 144 |
| 52. Supp. Figure 41 Sensitivity analysis of the risk of nonalcoholic fatty liver disease in patients with fatty pancreas.…………..…………..…...… | Page 145 |
| 53. Supp. Figure 42 Forest plot of metabolic syndrome prevalence in fatty pancreas…………………..……………..…………..………………….. | Page 146 |
| 54. Supp. Figure 43 Forest plot of metabolic syndrome prevalence in the population without fatty pancreas……..….…………..…………..……… | Page 147 |
| 55. Supp. Figure 44 Forest plot of the risk of metabolic syndrome in patients with fatty pancreas…………………….……..….…………..………. | Page 148 |
| 56. Supp. Figure 45 Sensitivity analysis of the risk of metabolic syndrome in patients with fatty pancreas..……………..….…………..…………... | Page 149 |
| 57. Supp. Figure 46 Forest plot of overweight prevalence in fatty pancreas…………………………...……………….…………………………….. | Page 150 |
| 58. Supp. Figure 47 Forest plot of overweight prevalence in the population without fatty pancreas ..….…………………………………....……..... | Page 150 |
| 59. Supp. Figure 48 Forest plot of the risk of overweight in patients with fatty pancreas….…….………….….…………..…………………...…..... | Page 151 |
| 60. Supp. Figure 49 Sensitivity analysis of the risk of overweight in patients with fatty pancreas…………….….…………………..……………… | Page 151 |
| 61. Supp. Figure 50 Forest plot of obesity prevalence in fatty pancreas…………………..…………..….………….….…………..………………… | Page 152 |
| 62. Supp. Figure 51 Forest plot of obesity prevalence in the population without fatty pancreas..………..………….….…………..………………… | Page 152 |
| 63. Supp. Figure 52 Forest plot of the risk of obesity in patients with fatty pancreas…………..…….…….……………..……………..…………… | Page 153 |
| 64. Supp. Figure 53 Sensitivity analysis of the risk of obesity in patients with fatty pancreas.………………….…….…..…………………..……… | Page 153 |
| 65. Supp. Figure 54 Forest plot of central obesity prevalence in fatty pancreas………………….…………….……...….………..……………...….. | Page 154 |
| 66. Supp. Figure 55 Forest plot of central obesity prevalence in the population without fatty pancreas……………….…….….…………..…...…… | Page 154 |
| 67. Supp. Figure 56 Forest plot of the risk of central obesity in patients with fatty pancreas………………..…….…….….…………….................... | Page 155 |
| 68. Supp. Figure 57 Sensitivity analysis of the risk of central obesity in patients with fatty pancreas……………….…….….…..…………..……… | Page 155 |
| 69. Supp. Figure 58 Forest plot of diabetes prevalence in fatty pancreas…………………………….………….….…………..…………………...... | Page 156 |
| 70. Supp. Figure 59 Forest plot of diabetes prevalence in the population without fatty pancreas….……………….………….……..………………. | Page 158 |
| 71. Supp. Figure 60 Forest plot of the risk of diabetes in patients with fatty pancreas………………….…….….…………..……………...….….…. | Page 160 |
| 72. Supp. Figure 61 Sensitivity analysis of the risk of diabetes in patients with fatty pancreas…………….….…….…….….……...………………. | Page 162 |
| 73. Supp. Figure 62 Forest plot of type 2 diabetes prevalence in fatty pancreas………………….……………….………………………………….… | Page 163 |
| 74. Supp. Figure 63 Forest plot of type 2 diabetes prevalence in the population without fatty pancreas………….…….….………………….……… | Page 164 |
| 75. Supp. Figure 64 Forest plot of the risk of type 2 diabetes in patients with fatty pancreas….……………………..………..……………….……… | Page 165 |
| 76. Supp. Figure 65 Sensitivity analysis of the risk of type 2 diabetes in patients with fatty pancreas….……………………..……………….……… | Page 166 |
| 77. Supp. Figure 66 Forest plot of insulin resistance prevalence in fatty pancreas……………………..….……………………..…...……….……….. | Page 167 |
| 78. Supp. Figure 67 Forest plot of insulin resistance prevalence in the population without fatty pancreas.……………….…….…….……………… | Page 167 |
| 79. Supp. Figure 68 Forest plot of the risk of insulin resistance in patients with fatty pancreas…….…..……………………….…………………… | Page 168 |
| 80. Supp. Figure 69 Sensitivity analysis of the risk of insulin resistance in patients with fatty pancreas………..……….…….….…………..……… | Page 168 |
| 81. Supp. Figure 70 Forest plot of hypertension in fatty pancreas……………………………….……….….….…………..………………………… | Page 169 |
| 82. Supp. Figure 71 Forest plot of hypertension in the population without fatty pancreas……….……………….…….…...………………….…….. | Page 170 |
| 83. Supp. Figure 72 Forest plot of the risk of hypertension in patients with fatty pancreas………….………..………….…….…..………………… | Page 171 |
| 84. Supp. Figure 73 Sensitivity analysis of the risk of hypertension in patients with fatty pancreas…….……...………….…….….………………... | Page 172 |
| 85. Supp. Figure 74 Forest plot of hyperlipidemia in fatty pancreas…………………………………………………………………………………... | Page 173 |
| 86. Supp. Figure 75 Forest plot of hyperlipidemia in the population without fatty pancreas………..…….….………………………………………. | Page 174 |
| 87. Supp. Figure 76 Forest plot of the risk of hyperlipidemia in patients with fatty pancreas……..…………..…….….…………………………….. | Page 175 |
| 88. Supp. Figure 77 Sensitivity analysis of the risk of hyperlipidemia in patients with fatty pancreas……...…………..…….….…………..………. | Page 176 |

**Supp.1 Search strategy**

**Pubmed**:

((echogenic pancreas[Title/Abstract]) OR (hyperechoic pancreas[Title/Abstract]) OR (fatty pancreas[Title/Abstract]) OR (pancreas steatosis[Title/Abstract]) OR (steatosis pancreas[Title/Abstract]) OR (pancreatic steatosis[Title/Abstract]) OR (pancreatic fat deposition[Title/Abstract]) OR (intrapancreatic fat deposition[Title/Abstract]) OR (fatty infiltration of the pancreas[Title/Abstract]) OR (fatty pancreatic disease[Title/Abstract]) OR (fatty pancreatic infiltration[Title/Abstract]) OR (pancreas fatty infiltration[Title/Abstract]) OR (pancreatic fatty replacement[Title/Abstract]) OR (pancreatic fatty infiltration[Title/Abstract]) OR (pancreas lipidosis[Title/Abstract]) OR (pancreas lipomatosis[Title/Abstract]) OR (pancreatic lipidosis[Title/Abstract]) OR (pancreatic lipomatosis[Title/Abstract]) OR (pancreatic lipomatous infiltration[Title/Abstract]) OR (pancreatic lipomatous lesion[Title/Abstract]) OR (pancreatic lipomatous pseudohypertrophy[Title/Abstract]) OR (pancreas lipomatous pseudo-hypertrophy[Title/Abstract])) OR (("echogenic pancreas") OR ("hyperechoic pancreas") OR ("fat pancreas") OR ("fatty pancreas") OR ("pancreas steatosis") OR ("pancreatic steatosis") OR ("pancreatic fat deposition") OR ("intrapancreatic fat deposition") OR ("pancreatic fat accumulation") OR ("fatty infiltration of the pancreas") OR ("fatty pancreatic disease") OR ("fatty pancreatic infiltration") OR ("pancreatic fatty replacement") OR ("pancreatic fatty infiltration") OR ("pancreatic fat infiltration") OR ("pancreas lipomatosis") OR ("pancreatic lipomatosis") OR ("pancreatic lipomatous pseudohypertrophy") OR ("intrapancreatic fat"))

**Embase**:

#1 'fatty pancreas'/exp

#2 'echogenic pancreas':ab,ti OR 'hyperechoic pancreas':ab,ti OR 'fatty pancreas':ab,ti OR 'pancreas steatosis':ab,ti OR 'steatosis pancreas':ab,ti OR 'pancreatic steatosis':ab,ti OR 'pancreatic fat deposition':ab,ti OR 'fatty infiltration of the pancreas':ab,ti OR 'fatty pancreatic disease':ab,ti OR 'fatty pancreatic infiltration':ab,ti OR 'pancreas fatty infiltration':ab,ti OR 'pancreatic fatty replacement':ab,ti OR 'pancreatic fatty infiltration':ab,ti OR 'pancreas lipidosis':ab,ti OR 'pancreas lipomatosis':ab,ti OR 'pancreatic lipidosis':ab,ti OR 'pancreatic lipomatosis':ab,ti OR 'pancreatic lipomatous infiltration':ab,ti OR 'pancreatic lipomatous lesion':ab,ti OR 'pancreatic lipomatous pseudohypertrophy':ab,ti OR 'pancreas lipomatous pseudo-hypertrophy':ab,ti OR 'intrapancreatic fat deposition':ab,ti

#3 'hyperechoic pancreas' OR 'fat pancreas' OR 'echogenic pancreas' OR 'fatty pancreas' OR 'pancreas steatosis' OR 'pancreatic steatosis' OR 'pancreatic fat deposition' OR 'pancreatic fat accumulation' OR 'fatty infiltration of the pancreas' OR 'fatty pancreatic disease' OR 'fatty pancreatic infiltration' OR 'pancreatic fatty replacement' OR 'pancreatic fatty infiltration' OR 'pancreatic fat infiltration' OR 'pancreas lipomatosis' OR 'pancreatic lipomatosis' OR 'pancreatic lipomatous pseudohypertrophy' OR 'intrapancreatic fat' OR 'intrapancreatic fat deposition'

#4 #1 OR #2 OR #3

**Cochrane**:

#1 (echogenic pancreas):ti,ab,kw OR (hyperechoic pancreas):ti,ab,kw OR (fatty pancreas):ti,ab,kw OR (pancreas steatosis):ti,ab,kw OR (steatosis pancreas):ti,ab,kw

#2 (pancreatic steatosis):ti,ab,kw OR (pancreatic fat deposition):ti,ab,kw OR (fatty infiltration of the pancreas):ti,ab,kw OR (fatty pancreatic disease):ti,ab,kw OR (fatty pancreatic infiltration):ti,ab,kw

#3 (pancreas fatty infiltration):ti,ab,kw OR (pancreatic fatty replacement):ti,ab,kw OR (pancreatic fatty infiltration):ti,ab,kw OR (pancreas lipidosis):ti,ab,kw OR (pancreas lipomatosis):ti,ab,kw

#4 (pancreatic lipomatosis):ti,ab,kw OR (pancreatic lipomatous infiltration):ti,ab,kw OR (pancreatic lipomatous lesion):ti,ab,kw OR (pancreatic lipomatous pseudohypertrophy):ti,ab,kw OR (pancreas lipomatous pseudo-hypertrophy):ti,ab,kw

#1 OR #2 OR #3 OR #4

**Supp. Table 1 Reported prevalence of fatty pancreas in six WHO regions (African region, European Region, Region of the Americas, Eastern Mediterranean Region, Western Pacific Region, South-East Asia Region)**

| **African region** | | | | | | | | | | | | | | | | | |
| --- | --- | --- | --- | --- | --- | --- | --- | --- | --- | --- | --- | --- | --- | --- | --- | --- | --- |
| Author | Country | Year of sample collection | | No. of participants | Fatty pancreas | Prevalence(%) | Methods for detection | | Enrolled  age group | Gender (male/  female) | Population | Human development index | | World Bank income level | | Diagnostic criteria for fatty pancreas | |
| Elhady et al, 2019^1^ | Egypt | | 01/2015-05/2017 | 50 | 29 | 58.00 | TUS | 10.6^c^ | | 27/23 | Obesity | HHD | | MICs | | The echo of the pancreas was enhanced and higher than that of the kidney cortex | |
| Hamed et al, 2022^2^ | Egypt | | - | 60 | 16 | 26.67 | TUS | 6.3±3.7 | | 20/40 | Cystic fibrosis | HHD | | MICs | | The echo of the pancreas was enhanced and higher than that of the liver | |
| Okasha et al, 2024^3^ | Egypt | | - | 95 | 75 | 78.95 | TUS | 44.0±11.0 | | 35/60 | Overweight and Obesity | HHD | | MICs | | The echo of the pancreas was enhanced and higher than that of the kidney cortex | |
| Osman et al, 2022 (1)^4^ | Egypt | | - | 150 | 18 | 12.00 | TUS | - | | 0/150 | Healthy population | HHD | | MICs | | The echo of the pancreas was enhanced and higher than that of liver or renal cortex | |
| Osman et al, 2022 (2)^4^ | Egypt | | - | 150 | 57 | 38.00 | TUS | 32.0(28.0-36.0)^b^ | | 0/150 | Polycystic Ovary Syndrome | | HHD | | MICs | | The echo of the pancreas was enhanced and higher than that of liver or renal cortex |
| Naguib et al, 2024^5^ | Egypt | | 08/2020-03/2023 | 200 | 125 | 62.50 | TUS | 48.8±8.1/  41.4±10.4^d^ | | 110/90 | MASLD/formerly NAFLD | HHD | | MICs | | The echo of the pancreas was enhanced and higher than that of the kidney cortex | |

| **Eastern Mediterranean Region** | | | | | | | | | | | | | | |
| --- | --- | --- | --- | --- | --- | --- | --- | --- | --- | --- | --- | --- | --- | --- |
| Author | Country | Year of sample collection | | No. of participants | Fatty pancreas | Prevalence(%) | Methods for detection | | Enrolled  age group | Gender (male/  female) | Population | Human development index | World Bank income level | Diagnostic criteria for fatty pancreas |
| Qureshi et al, 2022 (1)^6^ | Pakistan | | 10/2019-09/2020 | 35 | 12 | 34.29 | EUS | 51.1±12.5 | | 25/10 | Pancreatic adenocarcinoma | LMHD | MICs | Increased echogenicity of tail of pancreas compared with left kidney cortex and spleen |
| Qureshi et al, 2022 (2)^6^ | Pakistan | | 10/2019-09/2020 | 33 | 6 | 18.18 | EUS | 48.8±12.3 | | 19/14 | Healthy population | LMHD | MICs | Increased echogenicity of tail of pancreas compared with left kidney cortex and spleen |
| Noun et al, 2008^7^ | Lebanon | | 1999-2006 | 19 | 10 | 52.63 | HA | 61.7±13.0 | | 6/13 | Obesity | HHD | MICs | Surgical pathology specimens from the pancreatic neck of the obese group were studied further for the amount of intrapancreatic fat, and were graded from absent to massive on a 4-point pancreatic fat scale: 0-absent, 1-normal, 2-high, 3-massive |
| Sotoudehmanesh et al, 2019^8^ | Iran | | 01/2017-06/2018 | 228 | 59 | 25.88 | EUS | 56.6±15.4 | | 113/115 | Healthy population | HHD | MICs | Increased echogenicity of tail of pancreas compared with left kidney cortex and spleen |

| **European Region** | | | | | | | | | | | | | | |
| --- | --- | --- | --- | --- | --- | --- | --- | --- | --- | --- | --- | --- | --- | --- |
| Author | Country | Year of sample collection | | No. of participants | Fatty pancreas | Prevalence(%) | Methods for detection | | Enrolled  age group | Gender (male/  female) | Population | Human development index | World Bank income level | Diagnostic criteria for fatty pancreas |
| Blaho et al, 2022^9^ | Czech | | 03/2020-01/2021 | 34 | 22 | 64.71 | MRI | 44.6±8.2 | | 7/27 | Obesity | VHHD | HICs | Definition of MRI-PDFF > 10.4%. |
| Gjela et al, 2024 (1)^10^ | Denmark | | 10/2020-04/2022 | 35 | 4 | 11.43 | MRI | 45.7±7.8 | | 17/18 | Healthy population | VHHD | HICs | Definition of MRI-PDFF > 6.2% |
| Gjela et al, 2024 (2)^10^ | Denmark | | 10/2020-04/2022 | 62 | 43 | 69.35 | MRI | 46.4±7.4 | | 22/40 | Obesity | VHHD | HICs | Definition of MRI-PDFF > 6.2% |
| Previtali et al, 2023 (1)^11^ | France | | 01/2016-12/2019 | 60 | 28 | 46.67 | HA | 56.0±11.7 | | 27/33 | Pancreatectomy | VHHD | HICs | Fatty infiltration was defined as the presence of adipocytes that could be located between (extralobular fatty infiltration) or in the lobules of the pancreas (intralobular fatty infiltration) |
| Previtali et al, 2023 (2)^11^ | France | | 2019-2021 | 34 | 17 | 50.00 | HA | 49.5±16.2 | | 17/17 | Pancreatectomy | VHHD | HICs | Fatty infiltration was defined as the presence of adipocytes that could be located between (extralobular fatty infiltration) or in the lobules of the pancreas (intralobular fatty infiltration) |
| Rebours et al, 2015^12^ | France | | 2008-2011 | 110 | 56 | 50.91 | HA | - | | 47/63 | Pancreatic neuroendocrine neoplasms | VHHD | HICs | Perilobular fatty infiltration was defined as the presence of adipocytes within the interlobular space and was scored as follows: 0, no fatty infiltration; 1, some adipocytes; and 2, numerous adipocytes separating the lobules. Intralobular fatty infiltration was scored as 0, no or rare adipocytes in some lobules; 1, scattered adipocytes among most of the lobules; and 2, numerous adipocytes among most of the lobules forming clusters of more than 10 cells. The quantify the fat infiltration (ranging from 0 to 2) |
| Tranchart et al, 2012^13^ | France | | 06/2007-08/2008 | 103 | 24 | 23.30 | HA | 58.0(51.0-67.0)^b^ | | 58/45 | Pancreatoduodenectomy | VHHD | HICs | The score of pancreatic fatty infiltration was obtained by adding the scores of perilobular (ranging from 0 to 2) and intralobular fatty infiltration (ranging from 0 to 2). Samples with a global score ranging from 3 or 4 were considered fatty |
| Gaujoux et al, 2010^14^ | France | | 06/2004-12/2005 | 100 | 34 | 34.00 | HA | 58.0(16.0-82.0)^a^ | | 62/38 | Pancreatoduodenectomy | VHHD | HICs | Perilobular fatty infiltration was defined as the presence of adipocytes within the interlobular space and was scored as follows: 0, no fatty infiltration; 1, some adipocytes; 2, numerous adipocytes separating the lobules. Intralobular fatty infiltration was scored as 0, no or rare adipocytes in some lobules; 1, scattered adipocytes among most of the lobules; 2, numerous adipocytes among most of the lobules forming clusters of more than 10 cells. Samples with a global score that ranged from 3 or 4 were considered as fatty infiltration |
| Rosso et al, 2009^15^ | France | | 01/2004-12/2006 | 111 | 58 | 52.25 | HA | 65.0±11.0 | | 65/46 | Pancreatoduodenectomy | VHHD | HICs | The amount of fatty infiltration of the pancreas was expressed as a ratio of fat cells both intralobular and interlobular/overall tissue surface, > 10% of pancreatic fat |
| Harrell et al, 2020^16^ | Georgia | | 01/2010-05/2015 | 301 | 191 | 63.46 | HA | 63.2±11.5 | | 150/151 | Pancreatoduodenectomy | VHHD | MICs | Fat scores were determined by percentage of adipocyte collections in the specimen parenchyma and took into account both interlobular and intralobular fat content. Low fat, fat score < 2; high fat, fat score ≥ 2 |
| Sbeit et al, 2023^17^ | Israel | | 1/2010-9/2021 | 409 | 48 | 11.74 | CT | 54.6±21.3/57.6±16.8^d^ | | 253/156 | Pancreatitis | VHHD | HICs | Focal pancreatic steatosis can be presented as a hypo-attenuating mass lesion on CT |
| Sbeit et al, 2021^18^ | Israel | | 01/2018-01/2020 | 569 | 78 | 13.71 | EUS | 62.1±14.1/62.9±14.1^d^ | | 302/267 | Healthy population | VHHD | HICs | The diagnostic criterion for fatty pancreas was that the echo of the pancreas was enhanced and higher than that of the kidney cortex |
| Gullo et al, 2006^19^ | Italy | | - | 18 | 9 | 50.00 | TUS | 55.0(38.0-68.0)^a^ | | 12/6 | Pancreatic hyperenzymemia | VHHD | HICs | NA |
| Angrisani et al, 2022^20^ | Italy | | 09/2017-06/2020 | 73 | 41 | 56.16 | HA | 68.0(57.0-79.0)^b^ | | 37/36 | Pancreatoduodenectomy or distal pancreatectomy | VHHD | HICs | Pancreatic steatosis was graded into 4 categories: normal (0%-10%), mild lipomatosis (11%-40%), moderate lipomatosis (41%-70%), and severe lipomatosis (71%-100%) |
| Della et al, 2015^21^ | Italy | | 03/2014-11/2014 | 121 | 58 | 47.93 | TUS | 13.2±2.7 | | 73/48 | MASLD/formerly NAFLD | VHHD | HICs | The diagnostic criterion for fatty pancreas was that the echo of the pancreas was enhanced and higher than that of the kidney cortex |
| Mak et al, 2024^22^ | Netherlands | | - | 75 | 28 | 37.33 | MRI | 47.4(40.6-54.2)^b^ | | 45/31 | Healthy population | VHHD | HICs | Definition of MRI-PDFF > 6.2% |
| Engjom et al, 2018 (1)^23^ | Norway | | 12/2010-03/2016 | 21 | 12 | 57.14 | TUS | 28.3^c^ | | 10/11 | Cystic fibrosis | VHHD | HICs | The diagnostic criterion for fatty pancreas was that the echo of the pancreas was enhanced and higher than that of the liver |
| Engjom et al, 2018 (2)^23^ | Norway | | 12/2010-03/2016 | 15 | 5 | 33.33 | TUS | 38.2^c^ | | 7/8 | Healthy population | VHHD | HICs | The diagnostic criterion for fatty pancreas was that the echo of the pancreas was enhanced and higher than that of the liver |
| Marti-Aguado et al, 2023^24^ | Spain | | 2017-2022 | 324 | 292 | 90.12 | MRI | 55.0±11.0 | | 130/194 | MASLD/formerly NAFLD | VHHD | HICs | Definition of MRI-PDFF > 6.2% |
| Maetzel et al, 2023 (1)^25^ | Sweden | | - | 11 | 6 | 54.55 | MRI | 61.0(48.0-72)^a^ | | 8/5 | Healthy population | VHHD | HICs | Fatty pancreas was then defined as a pancreas with a fat fraction of > 6.2% |
| Maetzel et al, 2023 (2)^25^ | Sweden | | - | 13 | 11 | 84.62 | MRI | 61.0(48.0-81.0)^a^ | | 7/4 | MASLD/formerly NAFLD | VHHD | HICs | Fatty pancreas was then defined as a pancreas with a fat fraction of > 6.2% |
| Sezgin et al, 2024^26^ | Türkiye | | 05/2021-06/2021 | 1700 | 1171 | 68.88 | TUS | 48.0±20.9 | | 733/967 | Healthy population | VHHD | MICs | The diagnostic criterion for fatty pancreas was that the echo of the pancreas was enhanced and higher than that of the kidney cortex |
| Abbasoğlu et al, 2023^27^ | Türkiye | | 11/2015-11/2017 | 200 | 58 | 29.00 | MRI | 48.6±12.3 | | 64/136 | Healthy population | VHHD | MICs | Definition of MRI-PDFF > 6.2% |
| Ozturk et al, 2018 (1)^28^ | Türkiye | | - | 100 | 97 | 97.00 | TUS | 33.9±7.3 | | 97/3 | MASLD/formerly NAFLD | VHHD | MICs | The diagnostic criterion for fatty pancreas was that the echo of the pancreas was enhanced and higher than that of the kidney cortex |
| Ozturk et al, 2018 (2)^28^ | Türkiye | | - | 38 | 12 | 31.58 | TUS | 30.4±6.0 | | 34/4 | Healthy population | VHHD | MICs | The diagnostic criterion for fatty pancreas was that the echo of the pancreas was enhanced and higher than that of the kidney cortex |
| Uygun et al, 2015 (1)^29^ | Türkiye | | - | 84 | 43 | 51.19 | TUS | 39.4±6.8/37.6±7.0^d^ | | 64/20 | MASLD/formerly NAFLD | VHHD | MICs | The diagnostic criterion for fatty pancreas was that the echo of the pancreas was enhanced and higher than that of the kidney cortex. |
| Uygun et al, 2015 (2)^29^ | Türkiye | | - | 35 | 5 | 14.29 | TUS | 36.8±10.0 | | 24/11 | Healthy population | VHHD | MICs | The diagnostic criterion for fatty pancreas was that the echo of the pancreas was enhanced and higher than that of the kidney cortex |
| Gursoy et al, 2020^30^ | Türkiye | | 12/2016-12/2018 | 98 | 40 | 40.82 | CT | 37.5±7.5/34.7±9.2^d^ | | 60/38 | Liver transplant | VHHD | MICs | Fatty pancreas was defined as pancreatic and splenic attenuation ≤ -5 |
| Kirsoy et al, 2024^31^ | Türkiye | | - | 136 | 96 | 70.59 | TUS | 44.5±14.6 | | 52/84 | Overweight and Obesity | VHHD | MICs | The diagnostic criterion for fatty pancreas was that the echo of the pancreas was enhanced and higher than that of the kidney cortex |
| Koc et al, 2019^32^ | Türkiye | | 01/2016-12/2017 | 46 | 20 | 43.48 | CT | 66.5(36.0-88.0)^b^ | | 6/40 | Cholecystectomy | VHHD | MICs | The quantitative evaluation was calculated by dividing the mean ROI values measured from three anatomic regions (head-neck, body, and tail) by the ROI density values measured in the spleen on the same slices. A ratio of < 0.7 was accepted as pancreatic steatosis |
| Koc et al, 2020^33^ | Türkiye | | 01/2016-12/2017 | 637 | 202 | 31.71 | CT | 48^c^ | | 320/317 | Healthy population | VHHD | MICs | A quantitative evaluation was undertaken by dividing the mean region of interest (ROI) values measured from 3 anatomic regions (head-neck, body, and tail) by the ROI CT number values measured from the spleen on the same slices. A ratio of <0.7 was accepted as fatty pancreas |
| Doğan et al, 2023^34^ | Türkiye | | 2020-2021 | 461 | 151 | 32.75 | CT | - | | 255/206 | Healthy population | VHHD | MICs | Pancreatic index (Pancreatic density/spleen density) values less than 0.7 were taken to indicate steatosis |
| Ahbab et al, 2019 (1)^35^ | Türkiye | | 01/2016-01/2017 | 39 | 18 | 46.15 | CT | 55.3±14.2 | | - | Healthy population | VHHD | MICs | Defined as the difference in pancreas-spleen attenuation in negative HU value |
| Ahbab et al, 2019 (2)^35^ | Türkiye | | 01/2016-01/2017 | 43 | 31 | 72.09 | CT | 53.2±7.6 | | - | Prediabetes | VHHD | MICs | Defined as the difference in pancreas-spleen attenuation in negative HU value |
| Ahbab et al, 2019 (3)^35^ | Türkiye | | 01/2016-01/2017 | 28 | 19 | 67.86 | CT | 50.2±10.8 | | - | Diabetes | VHHD | MICs | Defined as the difference in pancreas-spleen attenuation in negative HU value |
| Altinmakas et al, 2021^36^ | Türkiye | | 07/2018-02/2020 | 322 | 85 | 26.40 | CT | 46.3±13.5 | | 163/159 | Healthy population | VHHD | MICs | Defined as the mean pancreatic density was at least 5 HU less than that of the spleen |
| Ulasoglu et al, 2021^37^ | Türkiye | | - | 345 | 227 | 65.80 | TUS | 50.7^c^ | | 125/220 | Healthy population | VHHD | MICs | The diagnostic criterion for fatty pancreas was that the echo of the pancreas was enhanced and higher than that of the kidney cortex |
| Bayyigit et al, 2024 (1)^38^ | Türkiye | | 07/2021-01/2022 | 30 | 19 | 63.33 | TUS | 40.3±9.3 | | 9/21 | Hypothyroidism | VHHD | MICs | The diagnostic criterion for fatty pancreas was that the echo of the pancreas was enhanced and higher than that of the kidney cortex |
| Bayyigit et al, 2024 (2)^38^ | Türkiye | | 07/2021-01/2022 | 30 | 18 | 60.00 | TUS | 43.1±14.6 | | 11/19 | Subclinical hypothyroidism | VHHD | MICs | The diagnostic criterion for fatty pancreas was that the echo of the pancreas was enhanced and higher than that of the kidney cortex |
| Bayyigit et al, 2024 (3)^38^ | Türkiye | | 07/2021-01/2022 | 33 | 8 | 24.24 | TUS | 40.3±9.3 | | 17/16 | Healthy population | VHHD | MICs | The diagnostic criterion for fatty pancreas was that the echo of the pancreas was enhanced and higher than that of the kidney cortex |
| Hasbal et al, 2024 (1)^39^ | Türkiye | | 12/2018-04/2023 | 304 | 23 | 7.57 | CT | 39.9±11.6 | | 195/109 | Kidney transplant | VHHD | MICs | Pancreatic steatosis was present if the pancreas-spleen attenuation ratio was < 0.7 |
| Hasbal et al, 2024 (2)^39^ | Türkiye | | 12/2018-04/2023 | 69 | 19 | 27.54 | CT | 48.2±13.4 | | 44/25 | Diabetes | VHHD | MICs | Pancreatic steatosis was present if the pancreas-spleen attenuation ratio was <0.7 |
| Guneyli et al, 2022^40^ | Türkiye | | 03/2020-12/2020 | 396 | 126 | 31.82 | CT | 52.5±16.9 | | 227/169 | Healthy population | VHHD | MICs | The pancreas-to-spleen attenuation ratio was found to be <0.70 |
| Öz et al, 2023^41^ | Türkiye | | - | 399 | 194 | 48.62 | CT | 54.3±14.1 | | 240/159 | Healthy population | VHHD | MICs | Difference between the mean densities of the pancreas and spleen < -5 HU |
| Sezgin et al, 2022^42^ | Türkiye | | 05/2019-03/2020 | 125 | 68 | 54.40 | TUS | 43.5±16.7 | | 48/77 | Healthy population | VHHD | MICs | The diagnostic criterion for fatty pancreas was that the echo of the pancreas was enhanced and higher than that of the kidney cortex |
| Sahin et al, 2022 (A)^43^ | Türkiye | | - | 99 | 49 | 49.49 | TUS | 61.0±10.0/64.0±11.0^d^ | | 74/25 | Acute Coronary Syndrome | VHHD | MICs | The diagnostic criterion for fatty pancreas was that the echo of the pancreas was enhanced and higher than that of the kidney cortex |
| Sahin et al, 2022 (N)^44^ | Türkiye | | - | 183 | 85 | 46.45 | TUS | 51.4±9.3/53.6±9.7^d^ | | 77/106 | MASLD/formerly NAFLD | VHHD | MICs | The diagnostic criterion for fatty pancreas was that the echo of the pancreas was enhanced and higher than that of the kidney cortex |
| Kul et al, 2019^45^ | Türkiye | | - | 103 | 54 | 52.43 | TUS | 52.0±10.0/49.0±8.0^d^ | | 38/65 | Healthy population | VHHD | MICs | Fatty pancreas was considered positive when pancreatic echogenicity exceeded renal echogenicity |
| Diamond et al, 2024 (1)^46^ | United Kingdom | | 01/2020-03/2022 | 112 | 70 | 62.50 | MRI | - | | - | Type 2 diabetes | VHHD | HICs | Definition of MRI-PDFF ≥4 % |
| Diamond et al, 2024 (2)^46^ | United Kingdom | | 01/2020-03/2022 | 91 | 11 | 12.09 | MRI | - | | - | Healthy population | VHHD | HICs | Definition of MRI-PDFF ≥4 % |
| Dong et al, 2024^47^ | United Kingdom | | 07/2014-01/2023 | 42599 | 7607 | 17.86 | MRI | 65.0(58.0-70.0)^b^ | | 19855/22744 | Healthy population | VHHD | HICs | The determination of fatty pancreas was derived from the sex- and age-specific 95th percentile upper limit of IPFD in the healthy population |
| Halle-Smith et al, 2017^48^ | United Kingdom | | 08/2008-12/2011 | 165 | 44 | 26.67 | HA | - | | 88/109 | Pancreatoduodenectomy or distal pancreatectomy | VHHD | HICs | The scoring criteria used for perilobular fatty infiltration were: 0 (no fatty infiltration), 1 (some adipocytes) and 2 (numerous adipocytes separating the lobules) and similarly for intralobular fibrosis: 0 (no or rare adipocytes in some lobules), 1 (scattered adipocytes among most of the lobules) and 2 (numerous adipocytes among most of the adipocytes forming clusters of more than 10 cells). Once added together, if the intralobular and perilobular fat scores were between 3 or 4 were deemed a high steatosis score and thus fatty |
| Karasu et al, 2021^49^ | Türkiye | | 01/2010-01/2018 | 62 | 33 | 53.23 | CT | - | | - | Pancreatoduodenectomy | VHHD | MICs | Pancreatic density/spleen density values less than 0.7 were taken to indicate steatosis |
| Ozbulbul et al, 2010^50^ | Türkiye | | 2010-2014 | 118 | 48 | 40.68 | CT | 53 (18-84)^a^ | | 72/46 | Healthy population | VHHD | MICs | The radiologist evaluated the CT scans visually to determine whether there was a fatty infiltration of the pancreas |

| **Region of the Americas** | | | | | | | | | | | | | | |
| --- | --- | --- | --- | --- | --- | --- | --- | --- | --- | --- | --- | --- | --- | --- |
| Author | Country | Year of sample collection | | No. of participants | Fatty pancreas | Prevalence(%) | Methods for detection | | Enrolled  age group | Gender (male/  female) | Population | Human Development Index | World Bank income level | Diagnostic criteria for fatty pancreas |
| Berger et al, 2023^51^ | Chile | | 2015 | 203 | 61 | 30.05 | CT | 55.0 (15.0-88.0)^a^ | | 96/107 | Healthy population | VHHD | HICs | Difference between the mean densities of the pancreas and spleen < -10 HU |
| Swauger et al, 2023^52^ | United States | | 05/2018-02/2020 | 128 | 24 | 18.75 | MRI | 12.1(9.0-14.8)^b^ | | 60/76 | Children | VHHD | HICs | Definition of MRI-PDFF > 6% |
| Trout et al, 2019^53^ | United States | | 08/2015-07/2017 | 86 | 8 | 9.30 | MRI | 14.2±3.2 | | 60/26 | MASLD/formerly NAFLD | VHHD | HICs | Definition of MRI-PDFF > 6% |
| Hoogenboom et al, 2021 (1)^54^ | United States | | 2010-2016 | 32 | 23 | 71.88 | CT | 68.1±11.6 | | 22/10 | Pancreatic adenocarcinoma | VHHD | HICs | Pancreatic steatosis was defined as a pancreas-to-spleen attenuation ratio ratio < 0.70 |
| Hoogenboom et al, 2021 (2)^54^ | United States | | 2010-2016 | 117 | 53 | 45.30 | CT | 68.6±10.3 | | 83/34 | Healthy population | VHHD | HICs | Pancreatic steatosis was defined as a pancreas-to-spleen attenuation ratio ratio < 0.70 |
| Mathur et al, 2011^55^ | United States | | 01/2002-12/2008 | 42 | 23 | 54.76 | CT | 46.0±11.0 | | 23/19 | Pancreatic adenocarcinoma | VHHD | HICs | Fatty infiltration of the pancreas was assessed by attenuation, which was measured in HU. Attenuation in CT was individually measured in the head, body and tail of the pancreas (pancreatic body CT attenuation of ≥30 HU) |
| Pham et al, 2016^56^ | United States | | 12/2011-12/2012 | 256 | 26 | 10.16 | CT | 10.3±4.7 | | 121/135 | Children | VHHD | HICs | An HU difference of -20 in comparison to the spleen was used as the cutoff for significant steatosis |
| Krill et al, 2022^57^ | United States | | 12/2014-10/2017 | 166 | 89 | 53.61 | EUS | 55.0(38.0-66.0)^b^ | | 55/111 | Healthy population | VHHD | HICs | Diagnosis of FP was established if there was a diffuse, homogenous increase in echogenicity in the pancreas compared to the spleen or liver |
| Sepe et al, 2011^58^ | United States | | 10/2009-01/2010 | 230 | 64 | 27.83 | EUS | 62.9±13.9 | | 102/128 | Healthy population | VHHD | HICs | Fatty pancreas was defined as pancreas in which > 80% of the parenchyma was moderately more hyperechoic as compared with the spleen, the main pancreatic duct margins were moderately obscured, and fine, salt and pepper dots in the pancreatic parenchyma were moderately blurry |
| Rosenblatt et al, 2019^59^ | United States | | 1999-2017 | 104 | 91 | 87.50 | TUS | 53.0±14.0 | | 37/67 | MASLD/formerly NAFLD | VHHD | HICs | The diagnostic criterion for fatty pancreas was that the echo of the pancreas was enhanced and higher than that of the kidney cortex |
| Gaujoux et al, 2012^60^ | United States | | 2000-2005 | 328 | 42 | 12.80 | HA | 71.0(63.0-77.0)^b^ | | 154/174 | Pancreatic adenocarcinoma | VHHD | HICs | The score of pancreatic fatty infiltration was obtained by adding the scores of perilobular (ranging from 0 to 2) and intralobular fatty infiltration (ranging from 0 to 2). Samples with a global score of 3 or 4 were classified as fatty |
| Becetti et al, 2024^61^ | USA | | - | 47 | 27 | 57.45 | CT | 18.29^c^ | | - | Obesity | VHHD | HICs | Pancreas-spleen difference ≤ -5 HU is suggestive of pancreatic steatosis |

| **South-East Asia Region** | | | | | | | | | | | | | | |
| --- | --- | --- | --- | --- | --- | --- | --- | --- | --- | --- | --- | --- | --- | --- |
| Author | Country | Year of sample collection | | No. of participants | Fatty pancreas | Prevalence(%) | Methods for detection | | Enrolled  age group | Gender (male/  female) | Population | Human development index | World Bank income level | Diagnostic criteria for fatty pancreas |
| Patel et al, 2019^62^ | India | | 10/2012-05/2014 | 46 | 20 | 43.48 | HA | 56.0(30.0-73.0)^a^ | | 30/16 | Pancreatoduodenectomy | LMHD | MICs | The scoring system is described as follows: the score of pancreatic fatty infiltration was obtained by adding the scores of perilobular and intralobular fatty infiltration. Perilobular fatty infiltration was defined as the presence of adipocytes within the interlobular space and was scored as follows: 0, no fatty infiltration; 1, some adipocytes; 2, numerous adipocytes separating the lobules. Intralobular fatty infiltration was scored as 0, no or rare adipocytes in some lobules; 1, scattered adipocytes among most of the lobules; 2. numerous adipocytes among most of the lobules forming clusters of more than ten cells. Samples with a global score that ranged from 0 to 2 were considered to be free of fatty infiltration, whereas those with a global score of 3 or 4 were considered to have fatty infiltration |
| Lesmana et al, 2015^63^ | Indonesia | | 01/2013-12/2013 | 901 | 315 | 34.96 | TUS | 43.1±12.2 | | 574/327 | Healthy population | HHD | MICs | Diagnosis of pancreas lipomatosis was established if there is increased echogenicity of the pancreas over the liver or renal cortex |
| Lesmana et al, 2018 (1)^64^ | Indonesia | | - | 43 | 34 | 79.07 | EUS | 62.0(51.5-72.5)^b^ | | 27/16 | Pancreatic cancer | HHD | MICs | The diagnostic criterion for fatty pancreas was that the echo of the pancreas was enhanced and higher than that of the kidney cortex |
| Lesmana et al, 2018 (2)^64^ | Indonesia | | - | 119 | 19 | 15.97 | EUS | 52.0(40.0-76.0)^b^ | | 60/59 | Healthy population | HHD | MICs | The diagnostic criterion for fatty pancreas was that the echo of the pancreas was enhanced and higher than that of the kidney cortex |

| **Western Pacific Region** | | | | | | | | | | | | |
| --- | --- | --- | --- | --- | --- | --- | --- | --- | --- | --- | --- | --- |
| Author | Country | Year of sample collection | No. of participants | Fatty pancreas | Prevalence(%) | Methods for detection | Enrolled  age group | Gender (male/  female) | Population | Human development index | World Bank income level | Diagnostic criteria for fatty pancreas |
| Sun et al. 2021^65^ | China | 01/2016-01/2020 | 337 | 117 | 34.72 | CT | 60.1±11.7 | 201/136 | Type 2 diabetes | HHD | MICs | Patients with low pancreas-to-spleen attenuation ratio ≤ 0.72 were defined as pancreatic steatosis |
| Bai et al, 2023^66^ | China | 2016-2020 | 21 | 8 | 38.10 | MRI | 30.0(21.0-48.0)^a^ | 13/8 | Obesity | HHD | MICs | Definition of MRI-PDFF > 10.4% |
| Chen et al, 2024^67^ | China | 03/2021-09/2023 | 40 | 5 | 12.50 | TUS | 40.0(21.0-59.0)^a^ | 22/18 | Healthy population | HHD | MICs | The diagnostic criterion for fatty pancreas was that the echo of the pancreas was enhanced and higher than that of the liver and kidney cortex |
| Chiyanika et al, 2020^68^ | China | 02/2014-03/2014 | 52 | 26 | 50.00 | MRI | 15.7±1.2 | 33/19 | MASLD/formerly NAFLD | HHD | MICs | Definition of MRI-PDFF > 5% |
| Bi et al, 2022^69^ | China | 2013 | 12551 | 1010 | 8.05 | TUS | 52.2±12.9/47.1±14.3^d^ | 8915/3636 | Healthy population | HHD | MICs | The diagnostic criterion for fatty pancreas was that the echo of the pancreas was enhanced and higher than that of the kidney cortex |
| Weng et al, 2018^70^ | China | 01/2015-10/2017 | 4418 | 488 | 11.05 | TUS | 49.5±9.2/47.2±6.9^d^ | 2287/2131 | Healthy population | HHD | MICs | The diagnostic criterion for fatty pancreas was that the echo of the pancreas was enhanced and higher than that of the kidney cortex |
| Hong et al, 2022 (1)^71^ | China | 05/2017-08/2019 | 961 | 572 | 59.52 | TUS | 50.0±10.9 | 0/961 | Breast cancer | HHD | MICs | The diagnostic criterion for fatty pancreas was that the echo of the pancreas was enhanced and higher than that of the kidney cortex |
| Hong et al, 2022 (2)^71^ | China | 05/2017-08/2019 | 1006 | 467 | 46.42 | TUS | 50.6±10.9 | 0/1006 | Healthy population | HHD | MICs | The diagnostic criterion for fatty pancreas was that the echo of the pancreas was enhanced and higher than that of the kidney cortex |
| Ke et al, 2024^72^ | China | 02/2018-11/2018 | 215 | 129 | 60.00 | TUS | 51.2^c^ | 0/215 | Breast cancer | HHD | MICs | The diagnostic criterion for fatty pancreas was that the echo of the pancreas was enhanced and higher than that of the liver |
| Xiao et al, 2024^73^ | China | 01/2016-09/2016 | 1774 | 840 | 47.35 | TUS | 44.53^c^ | 846/928 | Healthy population | HHD | MICs | The diagnostic criterion for fatty pancreas was that the echo of the pancreas was enhanced and higher than that of the kidney cortex |
| Li Y et al, 2023^74^ | China | 01/2013-05/2022 | 122 | 24 | 19.67 | CT | - | 52/70 | Pancreatic neuroendocrine neoplasms | HHD | MICs | The pancreatic index was calculated by dividing the mean CT value of the pancreas by the mean CT value of the spleen. PI less than 0.7 was considered pancreatic steatosis |
| Chen et al, 2022^75^ | China | 09/2012-08/2020 | 4704 | 57 | 1.21 | TUS | - | - | Healthy population | HHD | MICs | The diagnostic criterion for fatty pancreas was that the echo of the pancreas was enhanced and higher than that of the kidney cortex |
| Hung et al, 2018^76^ | China | 01/2005-12/2011 | 32346 | 2720 | 8.41 | TUS | 50.4±12.2 | 16857/15489 | Healthy population | HHD | MICs | The diagnostic criterion for fatty pancreas was that the echo of the pancreas was enhanced and higher than that of the liver |
| Luo et al, 2023^77^ | China | 12/2015-03/2018 | 147 | 88 | 59.86 | TUS | 47.5(37.0-58.0)/45.5±10.8^d^ | 126/21 | MASLD/formerly NAFLD | HHD | MICs | The diagnostic criterion for fatty pancreas was that the echo of the pancreas was enhanced and higher than that of the kidney cortex |
| Wen et al, 2022^78^ | China | 06/2018-11/2019 | 34 | 14 | 41.18 | MRI | 28.0(25.0-35.0)/26.0(21.0-31.0)^d^ | 16/18 | Healthy population | HHD | MICs | The pancreatic fat fraction greater than 6.2% was defined as fatty pancreas |
| Guo et al, 2019^79^ | China | 01/2014-12/2017 | 609 | 317 | 52.05 | HA | 54.6(18.0-82.0)^a^ | 383/226 | Pancreatoduodenectomy | HHD | MICs | The degree of pancreatic fat infiltration was assessed based on the percentage of the interlobular fat to total interlobular space and the percentage of the intralobular fat to total intralobular space. The sum of the intralobular and interlobular fat percentages was calculated. Pancreatic steatosis > 10% |
| Li C, et al, 2024^80^ | China | 12/2019-11/2022 | 128 | 96 | 75.00 | MRI | 33.4±7.7/29.9±6.2^d^ | 59/69 | Overweight and obesity | HHD | MICs | Pancreatic fat fraction ≥ 10.33% was defined as fatty pancreas |
| Wang et al, 2018^81^ | China | 2016 | 2093 | 56 | 2.68 | TUS | 52.7±12.0/45.4±14.2^d^ | - | Healthy population | HHD | MICs | Fatty pancreas: full shape, normal or slightly increased volume, blurred edges, a similar or slightly higher echogenicity to the adipose tissue in the area of the superior mesenteric artery |
| Dei et al, 2022^82^ | Japan | 2011-2020 | 221 | 92 | 41.63 | CT | - | 126/95 | Pancreatoduodenectomy | VHHD | HICs | The pancreatic fat ratio on CT images (I-PFR) was determined as the pancreatic fat volume-to-pancreatic volume. The optimal I-PFR cut-off value according to ROC curve was 3.2% |
| Tanaka et al, 2021^83^ | Japan | 01/2016-12/2017 | 150 | 74 | 49.33 | CT | 66.0(58.0-72.0)^a^ | 97/53 | Pancreatoduodenectomy | VHHD | HICs | Pancreatic fat volume was evaluated using the Hounsfield unit thresholds of - 200 to - 50. Definition of pancreatic fat volume/pancreatic volume ≥ 4.83%. |
| Kuriyama et al, 2024^84^ | Japan | 01/2010-03/2021 | 42 | 35 | 83.33 | CT | 66.2^c^ | 26/16 | Type 2 diabetes | VHHD | HICs | Defined as pancreatic and splenic attenuation < -5 |
| Okada et al, 2021^85^ | Japan | 04/2019-03/2020 | 919 | 430 | 46.79 | TUS | 59.5 ± 13.2 | 499/420 | Healthy population | VHHD | HICs | The diagnostic criterion for fatty pancreas was that the echo of the pancreas was enhanced and higher than that of the spleen and kidneys cortex |
| Mandai et al, 2019 (1)^86^ | Japan | 01/2012-11/2018 | 23 | 21 | 91.30 | TUS | 73.0(69.0-79.0)^b^ | 10/13 | Pancreatic adenocarcinoma | VHHD | HICs | The diagnostic criterion for fatty pancreas was that the echo of the pancreas was enhanced and higher than that of the kidney cortex |
| Mandai et al, 2019 (2)^86^ | Japan | 01/2012-11/2018 | 92 | 60 | 65.22 | TUS | 73.0(69.0-79.0)^b^ | 40/52 | Intraductal papillary mucinous neoplasm | VHHD | HICs | The diagnostic criterion for fatty pancreas was that the echo of the pancreas was enhanced and higher than that of the kidney cortex |
| Miura et al, 2020 (1)^87^ | Japan | 01/2011-02/2020 | 10 | 7 | 70.00 | HA | 67.3±9.2 | 6/4 | Pancreatic adenocarcinoma | VHHD | HICs | Interlobular fat infiltration was defined as the presence of adipocytes within the interlobular space and was scored as follows: 0, no fat infiltration; 1, some adipocytes present; 2, numerous adipocytes separating the lobules. Intralobular fat infiltration was scored as follows: 0, no or rare adipocytes present in some lobules; 1, scattered adipocytes present in the majority of lobules; 2, numerous adipocytes forming clusters of more than 10 cells present in the majority of lobules |
| Miura et al, 2020 (2)^87^ | Japan | 01/2011-02/2020 | 10 | 1 | 10.00 | HA | 60.9±11.6 | 7/3 | Pancreatic benign tumor | VHHD | HICs | Interlobular fat infiltration was defined as the presence of adipocytes within the interlobular space and was scored as follows: 0, no fat infiltration; 1, some adipocytes present; 2, numerous adipocytes separating the lobules. Intralobular fat infiltration was scored as follows: 0, no or rare adipocytes present in some lobules; 1, scattered adipocytes present in the majority of lobules; 2, numerous adipocytes forming clusters of more than 10 cells present in the majority of lobules |
| Kanno et al, 2018^88^ | Japan | 01/2006-12/2015 | 196 | 82 | 41.84 | HA | 68.8±9.5 | 111/89 | Pancreatic adenocarcinoma | VHHD | HICs | Histologic findings reveal fatty changes in localized regions of the pancreatic parenchyma |
| Silman et al, 2018 (1)^89^ | Japan | 01/2016-10/2017 | 460 | 85 | 18.48 | CT | 66.4±12.3 | 256/204 | Healthy population | VHHD | HICs | The pancreatic groove fat plane was evaluated using a four-point scale based on an estimation of how much of the fat plane was visualised between the medial border of the duodenum and the lateral portion of the pancreatic head on the 5 mm- and 1 mm-thick slices of the axial MDCT images (grade 1: visualisation of 0-25%, grade 2: 26-50%, grade 3: 51-75%, grade 4: 76-100%) |
| Silman et al, 2018 (2)^89^ | Japan | 01/2006-10/2017 | 25 | 2 | 8.00 | CT | 70.0±12.8 | 16/9 | Groove pancreatitis or carcinoma | VHHD | HICs | The pancreatic groove fat plane was evaluated using a four-point scale based on an estimation of how much of the fat plane was visualised between the medial border of the duodenum and the lateral portion of the pancreatic head on the 5 mm- and 1 mm-thick slices of the axial MDCT images (grade 1: visualisation of 0-25%, grade 2: 26-50%, grade 3: 51-75%, grade 4: 76-100%) |
| Fukuda et al, 2017^90^ | Japan | 02/2007-01/2017 | 43 | 14 | 32.56 | HA | 64.0(32.0-83.0)^a^ | 28/15 | Distal pancreatectomy | VHHD | HICs | The observed pathological changes were defined according to the criteria of our previous study as follows. When changes affected more than 5% of the sample, the sample was considered positive for fatty degeneration |
| Ookura et al, 2023^91^ | Japan | 04/2021-09/2021 | 200 | 88 | 44.00 | CT | 63.5(20.0-96.0)^a^ | 96/104 | Healthy population | VHHD | HICs | Grade 0: Normal. Pancreatic parenchyma is homogeneous, with smooth or mildly lobulated margins and no fat tissue is observed in the pancreatic parenchyma. Grade 1: Mild. The margin of the pancreas is serrated but the deep pancreatic parenchyma (around main pancreatic duct) is homogeneous. Mildly low-attenuated areas in the pancreatic parenchyma may be seen, but no coarse fat tissue is observed. Grade 2: Moderate. Coarse fat tissues are scattered and extends to the deep pancreatic parenchyma. Grade 3: Severe. More than half of the pancreas shows isoattenuation with fat |
| Tomita et al, 2014 (1)^92^ | Japan | 05/2008-09/2010 | 76 | 55 | 72.37 | HA | 66.0(35.0-88.0)^a^ | 41/35 | Pancreatic adenocarcinoma | VHHD | HICs | Fatty degeneration was categorized as grade 0 (0%-1% of fatty change in the pancreas), 1 (2%-5%), 2 (6%-10%), 3 (11%-15%), or 4 (>16%). Grades 0 and 1 weredefined as negative for fatty degeneration, grades ≥2 defined as fatty degeneration |
| Tomita et al, 2014 (2)^92^ | Japan | 05/2001-07/2011 | 98 | 43 | 43.88 | HA | 65.0(22.0-90.0)^a^ | 62/36 | Healthy population | VHHD | HICs | Fatty degeneration was categorized as grade 0 (0%-1% of fatty change in the pancreas), 1 (2%-5%), 2 (6%-10%), 3 (11%-15%), or 4 (>16%). Grades 0 and 1 weredefined as negative for fatty degeneration, grades ≥2 defined as fatty degeneration |
| Sano et al, 2022^93^ | Japan | 01/2010-12/2013 | 237 | 39 | 16.46 | HA | 69.0(38.0-88.0)^a^ | 156/81 | Pancreatoduodenectomy | VHHD | HICs | Specimens with fatty infiltration grade ranging from 0 to 2 and from 3 to 4 were defined as histological non-fatty pancreas and histological fatty pancreas, respectively. The degree of pancreatic fatty infiltration was graded from 0 to 4 by adding perilobular grades and intralobular grades. Perilobular fatty infiltration was defined as the presence of adipocytes within the interlobular space and was scored as follows: 0, no fatty infiltration; 1, some adipocytes; 2, numerous adipocytes separating the lobules. Intralobular fatty infiltration was defined as follows: 0, no or rare adipocytes; 1, scattered adipocytes; 2, numerous adipocytes among most of the lobules |
| Ishibashi et al, 2020^94^ | Japan | 04/2008-09/2018 | 56 | 47 | 83.93 | CT | 66.0(58.5-73.5)^b^ | 25/31 | Type 2 diabetes | VHHD | HICs | Fatty pancreas was defined as pancreatic and splenic attenuation ≤ -5 |
| Fujii et al, 2019^95^ | Japan | 2008 | 25274 | 2506 | 9.92 | TUS | 47.6^c^ | 11659/13615 | Healthy population | VHHD | HICs | The diagnostic criterion for fatty pancreas was that the echo of the pancreas was enhanced and higher than that of the liver |
| Horii et al, 2021^96^ | Japan | 04/2014-04/2020 | 22 | 11 | 50.00 | CT | 64.9±10.4 | 16/6 | Type 2 diabetes | VHHD | HICs | Fatty pancreas was defined as pancreatic and splenic attenuation ≤ -8 |
| Hori et al, 2014 (1)^97^ | Japan | 01/2004-12/2010 | 102 | 64 | 62.75 | HA | 63.5(56.0-69.0)^b^ | 60/42 | Pancreatic adenocarcinoma | VHHD | HICs | Fatty infiltraton (FI) areas were measured quantitatively as the percentage of area infiltrated by adipocytes relative to the total area on the section. FI > 20% is defined as fat pancreas |
| Hori et al, 2014 (2)^97^ | Japan | 01/2004-12/2010 | 85 | 30 | 35.29 | HA | 68.0(63.0-73.0)^b^ | 60/25 | Pancreatoduodenectomy | VHHD | HICs | Fatty infiltraton (FI) areas were measured quantitatively as the percentage of area infiltrated by adipocytes relative to the total area on the section. FI > 20% is defined as fat pancreas |
| Kawamura et al, 2022 (1)^98^ | Japan | 04/2017-10/2019 | 44 | 44 | 100.00 | EUS | 66.6±11.4 | 24/20 | Pancreatic cancer | VHHD | HICs | The average echogenicity of the pancreas was compared with that of the spleen or left kidney as the control, and the ratio of the echogenic value of the patient was calculated by dividing that of the pancreas by that of the control. The cut-off points of the pancreas-control ratio in EUS was determined to be 1.3 |
| Kawamura et al, 2022 (2)^98^ | Japan | 04/2017-10/2019 | 24 | 24 | 100.00 | EUS | 65.2±11.8 | 22/2 | Pancreatitis | VHHD | HICs | The average echogenicity of the pancreas was compared with that of the spleen or left kidney as the control, and the ratio of the echogenic value of the patient was calculated by dividing that of the pancreas by that of the control. The cut-off points of the pancreas-control ratio in EUS was determined to be 1.3 |
| Kawamura et al, 2022 (3)^98^ | Japan | 04/2017-10/2019 | 109 | 35 | 32.11 | EUS | 68.4±10.4 | 50/59 | Intraductal papillary mucinous neoplasm | VHHD | HICs | The average echogenicity of the pancreas was compared with that of the spleen or left kidney as the control, and the ratio of the echogenic value of the patient was calculated by dividing that of the pancreas by that of the control. The cut-off points of the pancreas-control ratio in EUS was determined to be 1.3 |
| Kawamura et al, 2022 (4)^98^ | Japan | 04/2017-10/2019 | 66 | 23 | 34.85 | EUS | 63.6±14.7 | 37/29 | Healthy population | VHHD | HICs | The average echogenicity of the pancreas was compared with that of the spleen or left kidney as the control, and the ratio of the echogenic value of the patient was calculated by dividing that of the pancreas by that of the control. The cut-off points of the pancreas-control ratio in EUS was determined to be 1.3. |
| Otsuka et al, 2023 (1)^99^ | Japan | 2007-2010 | 281 | 126 | 44.84 | CT | 53.9±5.5 | 152/129 | MASLD/formerly NAFLD | VHHD | HICs | Pancreatic density/spleen density values less than 0.8 were taken to indicate steatosis. |
| Otsuka et al, 2023 (2)^99^ | Japan | 2016-2019 | 38 | 18 | 47.37 | CT | 64.1±12.1 | 16/22 | Intraductal papillary mucinous neoplasm | VHHD | HICs | Pancreatic density/spleen density values less than 0.8 were taken to indicate steatosis. |
| Kobashi et al, 2021^100^ | Japan | 2011-2017 | 41 | 8 | 19.51 | CT | 74.8±10.5 | 20/21 | Pancreatic adenocarcinoma | VHHD | HICs | NA |
| Matsumoto et al, 2024^101^ | Japan | 01/2017-03/2020 | 42 | 32 | 76.19 | TUS | 70.5(30.0-82.0)^a^ | 24/18 | Pancreatoduodenectomy or distal pancreatectomy | VHHD | HICs | Hyperechoic: equal to or higher than the fat tissue of the retroperitoneum |
| Kobayashi et al, 2022^102^ | Japan | 01/2012-01/2015 | 138 | 54 | 39.13 | CT | 63.8±10.7 | 91/47 | Gastric cancer | VHHD | HICs | As a control, we used images of normal pancreas, which has an even and dense parenchyma with a smooth surface. Fatty pancreas was classified into two types according to the degree and distribution of low attenuation corresponding to the fat components: type S denoting superficial fat deposition with an irregular but dense parenchymal surface; and type D denoting diffuse fatty replacement with low attenuation areas unevenly distributed throughout the parenchyma |
| Makino et al, 2016^103^ | Japan | 2005-2006 | 472 | 208 | 44.07 | TUS | 60.8±9.4/56.9±9.8^d^ | 201/271 | Healthy population | VHHD | HICs | The echogenicity of the pancreatic body was compared with the liver |
| Choi et al, 2010^104^ | Korea | 01/2007-12/2007 | 284 | 110 | 38.73 | EUS | 52.1±12.2 | 102/182 | Healthy population | VHHD | HICs | The diagnostic criterion for fatty pancreas was that the echo of the pancreas was enhanced and higher than that of the kidney cortex |
| Park et al, 2022^105^ | Korea | 07/2015-05/2020 | 858 | 354 | 41.26 | CT | 66.6±16.8/58.4±17.4^d^ | 474/384 | Healthy population | VHHD | HICs | A threshold of < 36 HU of average pancreatic attenuation was considered as fatty pancreas |
| Chung et al, 2024^106^ | Korea | 11/2020-07/2022 | 527 | 157 | 29.79 | CT | 65.2±16.2 | 296/231 | Healthy population | VHHD | HICs | Defined as a pancreas-to-spleen attenuation of < 0.70 |
| Kang et al, 2019^107^ | Korea | 03/2006-08/2017 | 55 | 41 | 74.55 | TUS | 11.6±3.1 | 40/15 | MASLD/formerly NAFLD | VHHD | HICs | The degree of fatty pancreas was rated according to three grades as follows: 1) grade 0 (the echogenicity of pancreas is similar or slightly higher than that of the kidney); 2) grade 1 (pancreas echogenicity is much higher than that of the kidney, but lower than that of the retroperitoneal fat); and 3) grade 2 (pancreas echogenicity is similar to that of the retroperitoneal fat) |
| An et al, 2019^108^ | Korea | 09/2017-06/2018 | 544 | 341 | 62.68 | TUS | 31.3±4.8 | 310/234 | Healthy population | VHHD | HICs | The diagnostic criterion for fatty pancreas was that the echo of the pancreas was enhanced and higher than that of the kidney cortex |
| Chang et al, 2017^109^ | Korea | 06/2011-01/2013 | 57 | 36 | 63.16 | MRI | 60.5±11.9 | - | Distal pancreatectomy | VHHD | HICs | Pancreatic fat percentage was defined as the highest ratio between the area of intraparenchymal fat and the total area of the pancreatic parenchyma on the slide. Fatty pancreas > 5% |
| Oh et al, 2022^110^ | Korea | 12/2008-02/2014 | 1459 | 1039 | 71.21 | TUS | 47.3±10.6 | 854/605 | Healthy population | VHHD | HICs | The diagnostic criterion for fatty pancreas was that the echo of the pancreas was enhanced and higher than that of the liver and spleen |
| Lee et al, 2009^111^ | Korea | - | 293 | 180 | 61.43 | TUS | 44.9±9.5 | 127/166 | Obesity | VHHD | HICs | HU were measured at five different parts of the pancreas (head, neck, body, tail, and uncinate process) and three different parts of the spleen. The differences between the mean values of them were determined. If this difference was -5 or lower, it was classified into the fatty pancreas group |
| Hong et al, 2024^112^ | Korea | 04/2006-03/2021 | 94 | 30 | 31.91 | TUS | 65.0(57.0-72.0)^b^ | 44/50 | Pancreatectomy | VHHD | HICs | The diagnostic criterion for fatty pancreas was that the echo of the pancreas was enhanced and higher than that of the liver |
| Singh et al. 2019^113^ | New Zealand | - | 79 | 20 | 25.32 | MRI | 57.0(44.0-68.0)^b^ | 47/32 | Healthy population | VHHD | HICs | The 95th percentile of intra-pancreatic fat % in healthy non-obese individuals was considered as the upper limit and was used to determine the presence of fatty pancreas in the study cohort |
| Skudder-Hill et al, 2023^114^ | New Zealand | 2016-2020 | 346 | 321 | 92.77 | MRI | 50.5(36.7-60.0)^b^ | 145/201 | Overweight and Obesity | VHHD | HICs | Definition of pancreatic fat fraction > 6.2% |
| Cho et al, 2022^115^ | Korea | 01/2020-12/2020 | 102 | 27 | 26.47 | TUS | 12.5±3.2 | 72/30 | Children | VHHD | HICs | The diagnostic criterion for fatty pancreas was that the echo of the pancreas was enhanced and higher than that of the kidney cortex |
| Wong et al, 2014^116^ | China | 05/2008-09/2010 | 685 | 110 | 16.06 | MRI | 48.0±10.0 | 271/414 | Healthy population | HHD | MICs | Using the upper limit value of normal for pancreatic fat of 10.4 % ; The fat fraction was calculated as F/(W+F) × 100 % , where F and W were the signal intensities of fat and water, respectively |
| Sun et al, 2024^117^ | China | 01/2017-08/2022 | 139 | 90 | 64.75 | HA | 58.8±13.0 | 78/61 | Pancreatectomy | HHD | MICs | The degree of fatty pancreas was quantified as the ratio of intraparenchymal fat to the total pancreatic parenchyma area. Defined as intraparenchymal fat comprising >10 % of the total pancreatic tissue |
| Lin et al, 2024 (1)^118^ | China | - | 32 | 6 | 18.75 | TUS | 39.4±16.2 | 24/8 | Healthy population | HHD | MICs | The sonographic appearance of a pancreas affected by NAFPD includes a fuller morphology, normal or slightly enlarged size, blurred margins, and dense, bright internal echoes that are close to or stronger than those of the adipose tissue around the superior mesentericartery |
| Lin et al, 2024 (2)^118^ | China | - | 178 | 84 | 47.19 | TUS | 41.5±12.1 | 146/32 | Obstructive sleep apnoea | HHD | MICs | The sonographic appearance of a pancreas affected by NAFPD includes a fuller morphology, normal or slightly enlarged size, blurred margins, and dense, bright internal echoes that are close to or stronger than those of the adipose tissue around the superior mesentericartery |
| Chan et al, 2024^119^ | China | 01/2022-12/2022 | 101 | 30 | 29.70 | CT | 69.4±13.1/65.3±10.8^d^ | 51/50 | Pancreatic cancer | HHD | MICs | The diagnosis of a fatty pancreas was confirmed when the measuring area's average attenuation for the pancreas was below the spleen |

^a^Median (range), ^b^Median and interquartile, ^c^Mean, ^d^Fatty pancreas/Healthy population; TUS, Transabdominal ultrasonography; MRI, Magnetic resonance imaging; HA, Histology assessment; CT, Computed Tomography; EUS, Endoscopic ultrasound; MICs, middle-income countries; HICs, High-income countries; PDFF, proton density fat fraction; MASLD, metabolic dysfunction-associated steatotic liver disease; NAFLD, nonalcoholic fatty liver disease. In the included studies, NAFLD refers to the study-defined diagnosis reported by the original articles and is now generally termed MASLD; HHD, High human development; VHHD, Very high human development; LMHD, Low-medium human development; HU, Hounsfield units.

*The healthy population consisted of healthy controls and individuals undergoing medical examinations at the study sites.

| **Supp. Table 2 Bibliography of Repeat Populations** | | | | | | | | | | |
| --- | --- | --- | --- | --- | --- | --- | --- | --- | --- | --- |
| Author | Country | Year of sample collection | No. of participants | Fatty pancreas | Methods for detection | Enrolled  age group | Gender (male/  female) | Population | Diagnostic criteria for fatty pancreas | Repeated studies |
| Chan et al, 2022^120^ | China | 05/2008-09/2010 | 631 | 93 | MRI | 48.0±11.0 | 243/388 | Healthy population | Defined as having a pancreatic fat content of more than 10.4% on least-squares estimation (IDEAL)-MRI | Wong et al, 2014^116^ |
| Wang et al, 2014^121^ | China | 01/2009-12/2009 | 8097 | 1297 | TUS | 56.0±10.0/51.0±11.0^†^ | 4476/3621 | Healthy population | The diagnostic criterion for fatty pancreas was that the echo of the pancreas was enhanced and higher than that of the liver | Hung et al, 2018^76^ |

^†^Fatty pancreas/Healthy population; TUS, Transabdominal ultrasonography; MRI, Magnetic resonance imaging

Note: In meta-analyses, it is crucial to avoid including duplicate populations, as this can introduce bias into the results. During the literature screening process, we meticulously compared various factors such as recruitment locations, recruitment time, inclusion and exclusion criteria of study populations to assess whether overlapping cohorts were present across studies. After consulting with the authors of the relevant papers, we identified that the populations in two studies overlapped with those in Supp. Table 1. Consequently, these studies were excluded from the calculation of fatty pancreas prevalence. However, when assessing the risk of diseases such as obesity, central obesity, liver disease, and metabolic syndrome in patients with fatty pancreas compared to those without, the two studies were included in the analysis of disease risk if Supp. Table 1 did not assess these specific outcomes. This approach ensured adherence to the principle of not including duplicate populations.

| Author | Country | Year of sample collection | No. of participants | Fatty pancreas | Methods for detection | Enrolled  age group | Gender (male/  female) | Population | Diagnostic criteria for fatty pancreas |
| --- | --- | --- | --- | --- | --- | --- | --- | --- | --- |
| Al-Haddad et al, 2024^122^ | United States | - | 120 | 60 | EUS | 65.0(30.0-88.0)/66.0 (30.0-86.0)^†^ | 72/48 | Fat pancreas patients and healthy controls | The degree of pancreatic body and tail echogenicity was judged relative to the liver at a similar depth. If the liver seemed hyperechogenic, then pancreatic echogenicity was judged relative to the spleen |
| Han et al, 2023^123^ | United States | 08/2018-06/2019 | 115 | 99 | TUS | 48.0±11.4/38.6±12.4^†^ | 67/48 | Fat pancreas patients and healthy controls | The characteristics of the ultrasonic image of fatty pancreas were as follows: diffuse strong echoes of pancreatic parenchyma, normal or slightly increased volume, similar or slightly higher echogenicity compared to the adipose tissue in the area of the superior mesenteric artery |
| Tahtacı et al, 2018^124^ | Turkey | 2014-2016 | 56 | 31 | MRI | 66.0±1.6/65.8±1.6^†^ | 27/29 | Fat pancreas patients and healthy controls | For determining the severity of fatty pancreas , the percentage steatosis area with respect to total pancreatic volume was measured using the T1W and T2W sequences and classified in a 3-point scale: Score 1: 0%-33% steatosis of the whole pancreatic tissue, Score 2: 34%-66% steatosis of the whole pancreatic tissue, Score 3: 67%-100% steatosis of whole pancreatic tissue |

**Supp. Table 3 The case-control study of fatty versus non-fatty pancreas**

^†^Fatty pancreas/Healthy population; TUS, Transabdominal ultrasonography; MRI, Magnetic resonance imaging; EUS, Endoscopic ultrasound.

Note: The three studies in this table, which are case-control studies that included both fatty pancreas and healthy populations, do not reflect the incidence of fatty pancreas in the real population. Therefore, these studies were excluded from the calculation of fatty pancreas prevalence. However, they were included in the disease risk analysis when assessing the risk of diseases such as obesity, central obesity, liver disease and metabolic syndrome in patients with and without fatty pancreas.

| **Supp. Table 4 Quality assessment and study outcomes** | | | | | | |  |  |  |  |  |  |  |  |  |  |  |  |  |
| --- | --- | --- | --- | --- | --- | --- | --- | --- | --- | --- | --- | --- | --- | --- | --- | --- | --- | --- | --- |
| Author | Quality  Score | Prevalence of fatty pancreas | | Fatty liver | MASLD/formerly NAFLD | Metabolic syndrome | | Overweight | Obesity | Central obesity | Diabetes | Type 2 diabetes | Insulin resistance | Hypertension | Pancreatitis | Pancreatic cancer | Pancreatic adenocarcinoma | Hyperlipidemia |  |
| Elhady et al, 2019^1^ | 7 | Yes | - | | - | Yes | | - | - | - | - | - | Yes | - | - | - | - | - |  |
| Hamed et al, 2022^2^ | 7 | Yes | - | | - | - | | - | - | - | - | - | - | - | - | - | - | - |  |
| Okasha et al, 2024^3^ | 8 | Yes | Yes | | - | - | | - | - | - | - | - | - | - | - | - | - | - |  |
| Osman et al, 2022^4^ | 8 | Yes | Yes | | Yes | Yes | | - | - | - | Yes | - | Yes | Yes | - | - | - | - |  |
| Naguib et al, 2024^5^ | 7 | Yes | - | | - | Yes | | - | - | - | Yes | Yes | - | - | - | - | - | - |  |
| Noun et al, 2008^7^ | 6 | Yes | - | | - | - | | - | - | - | - | - | - | - | - | - | - | - |  |
| Qureshi et al, 2022^6^ | 6 | Yes | - | | - | - | | Yes | - | - | - | - | - | - | - | Yes | - | - |  |
| Sotoudehmanesh et al, 2019^8^ | 8 | Yes | Yes | | - | - | | - | - | - | Yes | - | - | Yes | - | - | - | Yes |  |
| Blaho et al, 2022^9^ | 6 | Yes | - | | - | Yes | | - | - | - | - | - | - | - | - | - | - | - |  |
| Gjela et al, 2024^10^ | 8 | Yes | - | | - | - | | - | - | - | - | - | - | - | - | - | - | - |  |
| Previtali et al, 2023^11^ | 7 | Yes | - | | - | - | | - | - | - | Yes | - | - | - | - | - | - | - |  |
| Rebours et al, 2015^12^ | 7 | Yes | - | | - | - | | - | - | - | - | - | - | - | - | - | - | - |  |
| Tranchart et al, 2012^13^ | 7 | Yes | - | | - | - | | - | - | - | - | - | - | - | - | - | - | - |  |
| Gaujoux et al, 2010^14^ | 8 | Yes | - | | - | - | | - | - | - | - | - | - | - | - | - | - | - |  |
| Rosso et al, 2009^15^ | 8 | Yes | - | | - | - | | - | - | - | Yes | - | - | - | - | - | - | - |  |
| Harrell et al, 2020^16^ | 8 | Yes | - | | - | - | | - | - | - | Yes | - | - | - | - | Yes | Yes | - |  |
| Sbeit et al, 2023^17^ | 7 | Yes | Yes | | - | - | | - | Yes | - | Yes | - | - | Yes | - | - | - | Yes |  |
| Sbeit et al, 2021^18^ | 8 | Yes | Yes | | - | - | | - | Yes | - | Yes | - | - | Yes | Yes | Yes | Yes | Yes |  |
| Gullo et al, 2006^19^ | 6 | Yes | - | | - | - | | - | - | - | - | - | - | - | - | - | - |  |  |
| Angrisani et al, 2022^20^ | 7 | Yes | - | | - | - | | - | - | - | - | - | - | - | - | - | - | - |  |
| Della et al, 2015^21^ | 7 | Yes | Yes | | - | - | | - | - | - | - | - | - | - | - | - | - | - |  |
| Mak et al, 2024^22^ | 6 | Yes | - | | - | - | | - | - | - | - | - | - | - | - | - | - | - |  |
| Engjom et al, 2018^23^ | 6 | Yes | - | | - | - | | - | - | - | - | - | - | - | - | - | - | - |  |
| Marti-Aguado et al, 2023^24^ | 8 | Yes | - | | - | - | | - | - | - | - | - | - | - | - | - | - | - |  |
| Maetzel et al, 2023^25^ | 6 | Yes | Yes | | Yes | - | | - | - | - | - | - | - | - | - | - | - | - |  |
| Sezgin et al, 2024^26^ | 8 | Yes | Yes | | - | Yes | | - | - | - | Yes | - | Yes | Yes | - | - | - | - |  |
| Abbasoğlu et al, 2023^27^ | 7 | Yes | - | | - | - | | - | - | - | - | - | - | - | - | - | - | - |  |
| Ozturk et al, 2018^28^ | 8 | Yes | Yes | | Yes | Yes | | - | - | - | Yes | - | - | - | - | - | - | - |  |
| Uygun et al, 2015^29^ | 7 | Yes | Yes | | Yes | - | | - | - | - | - | - | - | - | - | - | - | - |  |
| Gursoy et al, 2020^30^ | 6 | Yes | - | | - | - | | - | - | - | - | - | - | - | - | - | - | - |  |
| Kirsoy et al, 2024^31^ | 7 | Yes | Yes | | - | Yes | | Yes | Yes | - | Yes | - | Yes | - | - | - | - | - |  |
| Koc et al, 2019^32^ | 6 | Yes | - | | - | - | | - | - | - | - | - | - | - | - | - | - | - |  |
| Koc et al, 2020^33^ | 8 | Yes | - | | - | - | | - | - | - | - | - | - | - | - | - | - | - |  |
| Doğan et al, 2023^34^ | 8 | Yes | Yes | | - | - | | - | - | - | Yes | - | - | - | - | - | - | - |  |
| Ahbab et al, 2019^35^ | 7 | Yes | - | | - | - | | - | - | - | Yes | Yes | - | - | - | - | - | - |  |
| Altinmakas et al, 2021^36^ | 8 | Yes | Yes | | - | - | | - | - | - | - | - | - | - | - | - | - | - |  |
| Ulasoglu et al, 2021^37^ | 8 | Yes | Yes | | Yes | - | | - | - | - | Yes | - | Yes | - | - | - | - | - |  |
| Bayyigit et al, 2024^38^ | 7 | Yes | - | | - | - | | - | - | - | - | - | - | - | - | - | - | - |  |
| Hasbal et al, 2024^39^ | 8 | Yes | - | | - | - | | - | - | - | Yes | - | - | - | - | - | - | - |  |
| Guneyli et al, 2022^40^ | 7 | Yes | - | | - | - | | - | - | - | - | - | - | - | - | - | - | - |  |
| Öz et al, 2023^41^ | 8 | Yes | - | | - | - | | - | - | - | - | - | - | - | - | - | - | - |  |
| Karasu et al, 2021^49^ | 7 | Yes | - | | - | - | | - | - | - | - | - | - | - | - | - | - | - |  |
| Sezgin et al, 2022^42^ | 8 | Yes | Yes | | - | Yes | | - | - | - | Yes | - | - | - | - | - | - | Yes |  |
| Sahin et al, 2022 (A)^43^ | 7 | Yes | - | | - | - | | - | - | - | Yes | - | - | Yes | - | - | - | Yes |  |
| Sahin et al, 2022 (N)^44^ | 7 | Yes | - | | - | - | | - | - | - | Yes | - | - | Yes | - | - | - | Yes |  |
| Kul et al, 2019^45^ | 7 | Yes | - | | - | - | | - | - | - | Yes | - | - | Yes | - | - | - | Yes |  |
| Diamond et al, 2024^46^ | 8 | Yes | - | | - | - | | - | - | - | Yes | Yes | - | - | - | - | - | - |  |
| Dong et al, 2024^47^ | 9 | Yes | - | | - | - | | - | - | Yes | Yes | - | - | Yes | Yes | Yes | - | Yes |  |
| Halle-Smith et al, 2017^48^ | 7 | Yes | - | | - | - | | - | - | - | - | - | - | - | - | - | - | - |  |
| Berger et al, 2023^51^ | 7 | Yes | Yes | | - | - | | Yes | Yes | - | Yes | - | - | - | - | - | - | - |  |
| Swauger et al, 2023^52^ | 7 | Yes | - | | - | - | | - | - | - | - | - | - | - | Yes | - | - | - |  |
| Trout et al, 2019^53^ | 6 | Yes | - | | - | - | | - | - | - | - | - | - | - | - | - | - | - |  |
| Hoogenboom et al, 2021^54^ | 7 | Yes | - | | - | - | | - | - | - | - | - | - | - | - | Yes | Yes | - |  |
| Mathur et al, 2011^55^ | 6 | Yes | - | | - | - | | - | - | - | - | - | - | - | - | - | - | - |  |
| Pham et al, 2016^56^ | 8 | Yes | - | | - | - | | - | - | - | - | - | - | - | - | - | - | - |  |
| Krill et al, 2022^57^ | 7 | Yes | - | | - | - | | - | - | - | Yes | - | - | - | Yes | - | - | Yes |  |
| Sepe et al, 2011^58^ | 8 | Yes | Yes | | - | Yes | | - | - | - | Yes | - | - | Yes | Yes | Yes | - | Yes |  |
| Rosenblatt et al, 2019^59^ | 6 | Yes | - | | - | Yes | | - | - | - | Yes | - | - | Yes | - | - | - | - |  |
| Gaujoux et al, 2012^60^ | 8 | Yes | - | | - | - | | Yes | Yes | - | - | - | - | - | - | - | - | - |  |
| Patel et al, 2019^62^ | 6 | Yes | - | | - | - | | - | - | - | Yes | - | - | Yes | - | - | - | Yes |  |
| Lesmana et al, 2015^63^ | 6 | Yes | Yes | | - | - | | - | - | - | Yes | Yes | - | - | - | - | - | - |  |
| Lesmana et al, 2018^64^ | 7 | Yes | - | | - | - | | - | - | - | - | - | - | - | - | Yes | - | - |  |
| Sun et al. 2021^65^ | 8 | Yes | - | | - | - | | - | - | - | - | - | - | - | - | - | - | - |  |
| Bai et al, 2023^66^ | 5 | Yes | - | | - | - | | - | - | - | - | - | - | - | - | - | - | - |  |
| Chen et al, 2024^67^ | 6 | Yes | - | | - | - | | - | - | - | - | - | - | - | - | - | - | - |  |
| Wong et al, 2014^116^ | 9 | Yes | Yes | | - | Yes | | - | - | - | Yes | - | - | Yes | - | - | - | - |  |
| Chiyanika et al, 2020^68^ | 6 | Yes | - | | - | Yes | | - | - | - | - | - | Yes | - | - | - | - | - |  |
| Bi et al, 2022^69^ | 7 | Yes | Yes | | Yes | Yes | | - | Yes | - | Yes | - | - | Yes | - | - | - | Yes |  |
| Weng et al, 2018^70^ | 8 | Yes | Yes | | Yes | - | | - | - | Yes | Yes | Yes | - | Yes | - | - | - | - |  |
| Hong et al, 2022^71^ | 9 | Yes | - | | - | - | | - | - | - | - | - | - | - | - | - | - | - |  |
| Ke et al, 2024^72^ | 8 | Yes | - | | - | - | | - | - | - | - | - | - | - | - | - | - | - |  |
| Xiao et al, 2024^73^ | 7 | Yes | - | | - | - | | - | - | - | - | - | - | - | - | - | - | - |  |
| Li Y et al, 2023^74^ | 7 | Yes | Yes | | - | - | | - | - | - | - | - | - | - | - | - | - | - |  |
| Chen et al, 2022^75^ | 8 | Yes | Yes | | Yes | - | | - | - | - | Yes | Yes | - | Yes | - | - | - | - |  |
| Hung et al, 2018^76^ | 7 | Yes | Yes | | Yes | Yes | | - | - | Yes | Yes | - | - | Yes | - | - | - | - |  |
| Luo et al, 2023^77^ | 7 | Yes | - | | - | - | | - | - | - | - | - | - | - | - | - | - | - |  |
| Wen et al, 2022^78^ | 6 | Yes | - | | - | - | | - | - | - | - | - | - | - | - | - | - | - |  |
| Guo et al, 2019^79^ | 7 | Yes | - | | - | - | | - | - | - | - | - | - | - | - | - | - | - |  |
| Li C, et al, 2024^80^ | 7 | Yes | - | | - | - | | - | - | - | Yes | - | - | - | - | - | - | - |  |
| Wang et al, 2018^81^ | 8 | Yes | Yes | | - | Yes | | - | Yes | Yes | Yes | Yes | - | - | - | Yes | - | - |  |
| Dei et al, 2022^82^ | 6 | Yes | - | | - | - | | - | - | - | Yes | - | - | - | - | - | - | - |  |
| Tanaka et al, 2021^83^ | 7 | Yes | - | | - | - | | - | - | - | Yes | - | - | - | - | Yes | Yes | - |  |
| Kuriyama et al, 2024^84^ | 6 | Yes | - | | - | - | | - | - | - | - | - | - | - | - | - | - | - |  |
| Okada et al, 2021^85^ | 6 | Yes | Yes | | - | Yes | | - | - | - | Yes | - | - | Yes | - | - | - | Yes |  |
| Mandai et al, 2019^86^ | 7 | Yes | - | | - | - | | - | - | - | - | - | - | - | - | Yes | Yes | - |  |
| Miura et al, 2020^87^ | 6 | Yes | - | | - | - | | - | - | - | - | - | - | - | - | Yes | Yes | - |  |
| Kanno et al, 2018^88^ | 7 | Yes | - | | - | - | | - | - | - | - | - | - | - | - | - | - | - |  |
| Silman et al, 2018^89^ | 8 | Yes | - | | - | - | | - | - | - | - | - | - | - | - | - | - | - |  |
| Fukuda et al, 2017^90^ | 5 | Yes | - | | - | - | | - | - | - | - | - | - | - | - | - | - | - |  |
| Ookura et al, 2023^91^ | 8 | Yes | - | | - | - | | - | - | - | Yes | Yes | - | - | - | Yes | Yes | - |  |
| Tomita et al, 2014^92^ | 6 | Yes | - | | - | - | | - | - | - | Yes | - | - | - | - | - | - | - |  |
| Sano et al, 2022^93^ | 7 | Yes | - | | - | - | | - | - | - | - | - | - | - | - | - | - | - |  |
| Ishibashi et al, 2020^94^ | 6 | Yes | - | | - | - | | - | - | - | - | - | - | - | - | - | - | - |  |
| Fujii et al, 2019^95^ | 8 | Yes | - | | - | - | | - | - | - | - | - | - | - | Yes | - | - | - |  |
| Horii et al, 2021^96^ | 5 | Yes | Yes | | - | - | | - | - | - | - | - | - | - | - | - | - | - |  |
| Hori et al, 2014^97^ | 8 | Yes | - | | - | - | | - | - | - | - | - | - | - | - | Yes | Yes | - |  |
| Kawamura et al, 2022^98^ | 8 | Yes | Yes | | - | Yes | | - | - | - | - | - | - | - | Yes | Yes | - | - |  |
| Otsuka et al, 2023^99^ | 8 | Yes | Yes | | Yes | - | | - | - | - | Yes | Yes | - | Yes | - | Yes | - | Yes |  |
| Kobashi et al, 2021^100^ | 5 | Yes | - | | - | - | | - | - | - | - | - | - | - | - | - | - | - |  |
| Matsumoto et al, 2024^101^ | 5 | Yes | - | | - | - | | - | - | - | - | - | - | - | - | - | - | - |  |
| Kobayashi et al, 2022^102^ | 6 | Yes | - | | - | - | | - | - | - | - | - | - | - | - | - | - | - |  |
| Makino et al, 2016^103^ | 8 | Yes | Yes | | - | - | | - | - | - | - | - | Yes | - | - | - | - | - |  |
| Choi et al, 2010^104^ | 7 | Yes | Yes | | - | - | | - | - | - | Yes | Yes | - | Yes | - | - | - | - |  |
| Park et al, 2022^105^ | 8 | Yes | - | | - | - | | - | - | - | Yes | Yes | - | Yes | Yes | - | - | Yes |  |
| Chung et al, 2024^106^ | 8 | Yes | - | | - | - | | - | - | - | Yes | Yes | - | Yes | Yes | - | - | Yes |  |
| Kang et al, 2019^107^ | 6 | Yes | Yes | | - | - | | - | - | - | - | - | - | - | - | - | - | - |  |
| An et al, 2019^108^ | 8 | Yes | Yes | | Yes | Yes | | - | - | - | - | - | - | - | - | - | - | - |  |
| Chang et al, 2017^109^ | 6 | Yes | - | | - | - | | - | - | - | - | - | - | - | - | - | - | - |  |
| Oh et al, 2022^110^ | 7 | Yes | Yes | | Yes | - | | - | - | - | - | - | - | - | - | - | - | - |  |
| Lee et al, 2009^111^ | 7 | Yes | Yes | | - | Yes | | - | - | - | - | - | - | - | - | - | - | - |  |
| Hong et al, 2024^112^ | 6 | Yes | - | | - | - | | - | - | - | Yes | - | - | Yes | Yes | - | - | - |  |
| Singh et al. 2019^113^ | 6 | Yes | Yes | | - | - | | - | - | - | - | - | - | - | - | - | - | - |  |
| Skudder-Hill et al, 2023^114^ | 7 | Yes | - | | - | - | | - | - | - | - | - | - | - | - | - | - | - |  |
| Cho et al, 2022^115^ | 6 | Yes | Yes | | Yes | - | | - | Yes | - | - | - | - | - | - | - | - | - |  |
| Sun et al, 2024^117^ | 7 | Yes | - | | - | - | | - | - | - | - | - | - | - | - | - | - | - |  |
| Lin et al, 2024^118^ | 6 | Yes | Yes | | Yes | - | | - | - | - | Yes | - | - | Yes | - | - | - | - |  |
| Becetti et al. 2024^61^ | 6 | Yes | - | | - | - | | - | - | - | - | - | - | - | - | - | - | - |  |
| Ozbulbul et al, 2010^50^ | 6 | Yes | - | | - | - | | - | - | - | - | - | - | - | - | - | - | - |  |
| Chan et al, 2024^119^ | 6 | Yes | Yes | | - | - | | - | - | - | Yes | - | - | - | Yes | - | - | - |  |
| Chan et al, 2022^120^ | 8 | - | - | | - | - | | - | - | Yes | - | - | - | - | - | - | - | Yes |  |
| Wang et al, 2014^121^ | 7 | - | - | | - | - | | - | Yes | - | - | - | - | - | - | - | - | - |  |
| Al-Haddad et al, 2024^122^ | 6 | - | Yes | | - | - | | - | - | - | Yes | - | - | Yes | - | - | - | - |  |
| Han et al, 2023^123^ | 7 | - | Yes | | - | - | | - | Yes | - | Yes | - | - | - | - | - | - | Yes |  |
| Tahtacı et al, 2018^124^ | 5 | - | Yes | | Yes | - | | - | - | - | - | - | - | - | - | - | - | - |  |

NAFLD, Nonalcoholic fatty liver disease

| **Supp. Table 5 Meta-regression analyses of potential sources of heterogeneity in fatty pancreas prevalence in the overall study population** | | | | | |
| --- | --- | --- | --- | --- | --- |
| Variables | N studies | Univariate analysis | | Multivariate analysis | |
|  |  | *p* value | R² (%) | *p* value | R² (%) |
| Multivariate global effect | 119 | *-* | - | 0.002 | 30.28 |
| Year of publication | 119 | 0.269 | 0.52 |  |  |
| Sample size | 119 | 0.001 | 10.35 |  |  |
| World Bank income level | 119 | 0.584 | -0.80 |  |  |
| Country | 119 | 0.263 | 7.8 |  |  |
| Population | 119 | 0.010 | 23.23 |  |  |
| Diagnostic modality | 119 | 0.376 | 0.34 |  |  |
| Human Development Index | 119 | 0.266 | 1.61 |  |  |
| WHO regions | 119 | 0.717 | -1.63 |  |  |
| Note: Meta-regression analyses were conducted on Freeman–Tukey-transformed prevalence using restricted maximum likelihood with Knapp–Hartung adjustment to explore sources of heterogeneity. Publication year was included as a predefined moderator. Temporal meta-regression in Figure 4 was conducted separately using raw study-level proportions.  Univariate analysis   1. Publication year   Meta-regression Number of obs = 119  REML estimate of between-study variance tau2 = .0378  % residual variation due to heterogeneity I-squared_res = 96.95%  Proportion of between-study variance explained Adj R-squared = 0.52%  With Knapp-Hartung modification  -----------------------------------------------------------------------------------------------------------  _ES \| Coef. Std. Err. t P>\|t\| [95% Conf. Interval]  ----------------+------------------------------------------------------------------------------------------  Publication year \| .0054204 .0048798 1.11 0.269 -.0042438 .0150845  _cons \| -10.52447 9.857092 -1.07 0.288 -30.04593 8.996981  -----------------------------------------------------------------------------------------------------------   2. Sample size   Meta-regression Number of obs = 119  REML estimate of between-study variance tau2 = .03407  % residual variation due to heterogeneity I-squared_res = 96.41%  Proportion of between-study variance explained Adj R-squared =10.35%  With Knapp-Hartung modification  --------------------------------------------------------------------------------------------------------  _ES \| Coef. Std. Err. t P>\|t\| [95% Conf. Interval]  -------------+------------------------------------------------------------------------------------------  Sample size \| -.0000107 3.13e-06 -3.41 0.001 -.0000169 -4.48e-06  _cons \| .4405397 .0194611 22.64 0.000 .4019981 .4790813  --------------------------------------------------------------------------------------------------------   3. World Bank income level   Meta-regression Number of obs = 119  REML estimate of between-study variance tau2 = .03831  % residual variation due to heterogeneity I-squared_res = 97.01%  Proportion of between-study variance explained Adj R-squared = -0.80%  Joint test for all covariates Model F(2,116) = 0.54  With Knapp-Hartung modification Prob > F = 0.5841  -----------------------------------------------------------------------------------------------------------------  _ES \| Coef. Std. Err. t P>\|t\| [95% Conf. Interval]  ----------------------+------------------------------------------------------------------------------------------  High income \| -.0259445 .0861153 -0.30 0.764 -.1965067 .1446178  Upper middle income \| -.0633223 .0872961 -0.73 0.470 -.2362232 .1095786  Lower middle income \| Reference  _cons \| .4645943 .0816925 5.69 0.000 .302792 .6263966  -----------------------------------------------------------------------------------------------------------------   4. Country   Meta-regression Number of obs = 119  REML estimate of between-study variance tau2 = .03504  % residual variation due to heterogeneity I-squared_res = 95.24%  Proportion of between-study variance explained Adj R-squared = 7.80%  Joint test for all covariates Model F(23,95) = 1.20  With Knapp-Hartung modification Prob > F = 0.2623  --------------------------------------------------------------------------------------------------------  _ES \| Coef. Std. Err. t P>\|t\| [95% Conf. Interval]  -------------+------------------------------------------------------------------------------------------  Chile \| -.4078407 .3403024 -1.20 0.234 -1.083426 .2677449  China \| -.3713723 .2789606 -1.33 0.186 -.9251791 .1824344  Czech \| -.0612745 .3740016 -0.16 0.870 -.8037615 .6812126  Denmark \| -.2237972 .3480634 -0.64 0.522 -.9147904 .4671959  Egypt \| -.2092237 .2913614 -0.72 0.474 -.7876493 .3692019  France \| -.2912262 .2911846 -1.00 0.320 -.8693007 .2868483  Georgia \| -.0737818 .3379474 -0.22 0.828 -.7446923 .5971286  India \| -.2735507 .3638641 -0.75 0.454 -.9959122 .4488108  Indonesia \| -.3692151 .3083932 -1.20 0.234 -.981453 .2430228  Iran \| -.4495614 .3395115 -1.32 0.189 -1.123577 .2244542  Israel \| -.5810212 .3072796 -1.89 0.062 -1.191048 .0290059  Italy \| -.1939464 .3067099 -0.63 0.529 -.8028425 .4149497  Japan \| -.2624706 .2790662 -0.94 0.349 -.8164871 .2915459  Korea \| -.2105389 .2827607 -0.74 0.458 -.7718898 .350812  Lebanon \| -.1820175 .402708 -0.45 0.652 -.981494 .617459  Netherlands \| -.335 .3523304 -0.95 0.344 -1.034464 .3644643  New Zealand \| -.0796595 .3113911 -0.26 0.799 -.6978491 .53853  Norway \| -.2361111 .3718722 -0.63 0.527 -.9743708 .5021486  Pakistan \| -.4436274 .354246 -1.25 0.214 -1.146895 .2596398  Spain \| .1929013 .3375994 0.57 0.569 -.4773183 .8631208  Türkiye \| -.244946 .2788244 -0.88 0.382 -.7984823 .3085904  United Kingdom \| -.4313949 .2978118 -1.45 0.151 -1.022626 .1598363  United States \| -.3399996 .2832733 -1.20 0.233 -.9023682 .2223691  Sweden \| Reference  _cons \| .7083333 .2754161 2.57 0.012 .1615633 1.255103  --------------------------------------------------------------------------------------------------------   5. Population   Meta-regression Number of obs = 119  REML estimate of between-study variance tau2 = .02918  % residual variation due to heterogeneity I-squared_res = 96.42%  Proportion of between-study variance explained Adj R-squared = 23.23%  Joint test for all covariates Model F(30,88) = 1.91  With Knapp-Hartung modification Prob > F = 0.0104  --------------------------------------------------------------------------------------------------------  _ES \| Coef. Std. Err. t P>\|t\| [95% Conf. Interval]  -------------+------------------------------------------------------------------------------------------  Acute Coronary Syndrome \| .3775901 .2661664 1.42 0.160 -.1513598 .90654  Breast cancer \| .4826406 .2558021 1.89 0.062 -.0257122 .9909935  Children \| .0627266 .2089727 0.30 0.765 -.3525627 .478016  Cholecystectomy \| .3174232 .2868792 1.11 0.272 -.252689 .8875354  Cystic fibrosis \| .1493073 .2780706 0.54 0.593 -.4032996 .7019141  Diabetes \| .4862537 .2082841 2.33 0.022 .0723329 .9001746  Diabetes+Kidney transplant \| -.0047589 .2519359 -0.02 0.985 -.5054286 .4959108  Gastric cancer \| .2739449 .260796 1.05 0.296 -.2443323 .7922221  Healthy population \| .2162539 .1801051 1.20 0.233 -.1416671 .5741748  Healthy population+Breast cancer \| .4108561 .2475936 1.66 0.101 -.0811841 .9028963  Healthy population+Cystic fibrosis \| .3548628 .2969707 1.19 0.235 -.235304 .9450296  Healthy population+Diabetes \| .2816554 .2563364 1.10 0.275 -.2277593 .79107  Healthy population+Hypothyroidism+Subclinical hypothyroidism \| .3665115 .2673752 1.37 0.174 -.1648405 .8978636  Healthy population+MASLD/formerly NAFLD \| .5049601 .2150315 2.35 0.021 .0776302 .93229  Healthy population+Overweight and obestiy \| .3671767 .2665534 1.38 0.172 -.1625423 .8968956  Healthy population+Pancreatic cancer \| .3067156 .20225 1.52 0.133 -.0952137 .7086449  Healthy population+Pancreatic cancer+Pancreatitis+IPMN \| .4011591 .2547569 1.57 0.119 -.1051168 .907435  Healthy population+Polycystic Ovary Syndrome \| .1326406 .2532234 0.52 0.602 -.3705878 .635869  Healthy population+Prediabetes+Diabetes \| .5008224 .2642803 1.90 0.061 -.0243792 1.026024  Liver transplant \| .2908039 .266358 1.09 0.278 -.2385268 .8201345  MASLD/formerly NAFLD \| .4765112 .1893563 2.52 0.014 .1002053 .8528171  MASLD/formerly NAFLD+IPMN \| .3340512 .2528324 1.32 0.190 -.1684 .8365025  Overweight and obestiy \| .564891 .1899164 2.97 0.004 .1874721 .9423098  Pancreatic cancer \| .1888795 .1992727 0.95 0.346 -.207133 .5848921  Pancreatic cancer+IPMN \| .5869884 .2635381 2.23 0.028 .0632618 1.110715  Pancreatic cancer+Pancreatic benign tumor \| .2826406 .3310209 0.85 0.396 -.3751938 .940475  Pancreatic cancer+Pancreatoduodenectomy and/or distal pancreatectomy \| .3853144 .257153 1.50 0.138 -.1257231 .8963519  Pancreatic hyperenzymemia \| .3826406 .3388929 1.13 0.262 -.2908378 1.056119  Pancreatic neuroendocrine neoplasms \| .2337144 .2247374 1.04 0.301 -.212904 .6803328  Pancreatoduodenectomy and/or distal pancreatectomy \| .3377609 .1838343 1.84 0.070 -.0275712 .7030929  Pancreatitis \| Reference  _cons \| .1173594 .177815 0.66 0.511 -.2360105 .4707293  --------------------------------------------------------------------------------------------------------   6. Diagnostic modality   Meta-regression Number of obs = 119  REML estimate of between-study variance tau2 = .03787  % residual variation due to heterogeneity I-squared_res = 96.75%  Proportion of between-study variance explained Adj R-squared = 0.34%  Joint test for all covariates Model F(4,114) = 1.07  With Knapp-Hartung modification Prob > F = 0.3760  ----------------------------------------------------------------------------------------------------------------  _ES \| Coef. Std. Err. t P>\|t\| [95% Conf. Interval]  ---------------------+------------------------------------------------------------------------------------------  CT \| .0431389 .0826783 0.52 0.603 -.1206461 .206924  HA \| .0905577 .0891485 1.02 0.312 -.0860448 .2671603  MRI \| .1153348 .0903643 1.28 0.204 -.0636761 .2943457  TUS \| .1231812 .0804019 1.53 0.128 -.0360944 .2824569  EUS \| Reference  _cons \| .3380221 .0734958 4.60 0.000 .1924275 .4836167  ---------------------------------------------------------------------------------------------------   7. Human Development Index   Meta-regression Number of obs = 119  REML estimate of between-study variance tau2 = .03739  % residual variation due to heterogeneity I-squared_res = 96.86%  Proportion of between-study variance explained Adj R-squared = 1.61%  Joint test for all covariates Model F(3,115) = 1.34  With Knapp-Hartung modification Prob > F = 0.2660  --------------------------------------------------------------------------------------------------------  _ES \| Coef. Std. Err. t P>\|t\| [95% Conf. Interval]  -------------------------------------------------------------------------------------------------------  High human development \| -.0713869 .2457423 -0.29 0.772 -.558155 .4153812  Low human development \| -.1700767 .3329949 -0.51 0.611 -.8296755 .4895221  Very high human development \| .0128428 .2437674 0.05 0.958 -.4700134 .495699  Medium human development \| Reference  _cons \| .4347826 .2426854 1.79 0.076 -.0459306 .9154958  --------------------------------------------------------------------------------------------------------   8. WHO regions   Meta-regression Number of obs = 119  REML estimate of between-study variance tau2 = .03862  % residual variation due to heterogeneity I-squared_res = 96.92%  Proportion of between-study variance explained Adj R-squared = -1.63%  Joint test for all covariates Model F(5,113) = 0.58  With Knapp-Hartung modification Prob > F = 0.7168  --------------------------------------------------------------------------------------------------------  _ES \| Coef. Std. Err. t P>\|t\| [95% Conf. Interval]  -------------+------------------------------------------------------------------------------------------  African region \| .1355507 .1592494 0.85 0.396 -.179951 .4510524  Eastern Mediterranean Region \| -.0470407 .1854766 -0.25 0.800 -.4145033 .3204219  European Region \| .0853974 .129282 0.66 0.510 -.1707336 .3415283  Region of the Americas \| -.001281 .1409938 -0.01 0.993 -.2806151 .2780531  Western Pacific Region \| .0563838 .1281919 0.44 0.661 -.1975874 .3103549  South-East Asia Region \| Reference  _cons \| .3638804 .1248721 2.91 0.004 .1164862 .6112746  Multivariate analysis   9. Sample size+ Population   Meta-regression Number of obs = 119  REML estimate of between-study variance tau2 = .02649  % residual variation due to heterogeneity I-squared_res = 96.20%  Proportion of between-study variance explained Adj R-squared = 30.28%  Joint test for all covariates Model F(31,87) = 2.25  With Knapp-Hartung modification Prob > F = 0.0017  --------------------------------------------------------------------------------------------------------  _ES \| Coef. Std. Err. t P>\|t\| [95% Conf. Interval]  -------------+-----------------------------------------------------------------------------------------  Sample size \| -7.93e-06 2.89e-06 -2.74 0.007 -.0000137 -2.18e-06  Acute Coronary Syndrome \| -.0480731 .2521447 -0.19 0.849 -.549238 .4530919  Breast cancer \| .057897 .2411713 0.24 0.811 -.421457 .5372509  Children \| -.3627657 .1954047 -1.86 0.067 -.7511537 .0256223  Cholecystectomy \| -.1086601 .2739229 -0.40 0.693 -.6531116 .4357914  Cystic fibrosis \| -.276665 .2646826 -1.05 0.299 -.8027503 .2494203  Diabetes \| .0583612 .1950908 0.30 0.766 -.3294029 .4461254  Diabetes+Kidney transplant \| -.4282501 .2370573 -1.81 0.074 -.8994272 .0429269  Gastric cancer \| -.1514091 .2464665 -0.61 0.541 -.6412879 .3384697  Healthy population \| -.1794088 .1666924 -1.08 0.285 -.5107281 .1519104  Healthy population+Cystic fibrosis \| -.0712997 .2844749 -0.25 0.803 -.6367244 .4941249  Healthy population+Diabetes \| -.1431834 .2417386 -0.59 0.555 -.6236651 .3372983  Healthy population+Hypothyroidism+Subclinical hypothyroidism \| -.0591992 .2534208 -0.23 0.816 -.5629004 .4445021  Healthy population+MASLD/formerly NAFLD \| .0789059 .2017247 0.39 0.697 -.3220439 .4798556  Healthy population+Overweight and obestiy \| -.0585023 .2525533 -0.23 0.817 -.5604794 .4434748  Healthy population+Pancreatic cancer \| -.1180668 .1887901 -0.63 0.533 -.4933075 .2571739  Healthy population+Pancreatic cancer+Pancreatitis+IPMN \| -.0233626 .2400608 -0.10 0.923 -.5005094 .4537841  Healthy population+Polycystic Ovary Syndrome \| -.2914293 .2384294 -1.22 0.225 -.7653335 .1824749  Healthy population+Prediabetes+Diabetes \| .0752465 .2501522 0.30 0.764 -.4219582 .5724511  Liver transplant \| -.1348672 .252347 -0.53 0.594 -.6364343 .3666999  MASLD/formerly NAFLD \| .0518691 .1759687 0.29 0.769 -.2978878 .4016261  MASLD/formerly NAFLD+IPMN \| -.089868 .2380129 -0.38 0.707 -.5629444 .3832083  Overweight and obestiy \| .1413412 .1766038 0.80 0.426 -.209678 .4923604  Pancreatic cancer \| -.2370741 .185906 -1.28 0.206 -.6065824 .1324343  Pancreatic cancer+IPMN \| \| .1614521 .2493677 0.65 0.519 -.3341931 .6570973  Pancreatic cancer+Pancreatic benign tumor \| -.1436488 .3198589 -0.45 0.654 -.779403 .4921054  Pancreatic cancer+Pancreatoduodenectomy and/or distal pancreatectomy \| -.0396512 .2426054 -0.16 0.871 -.5218556 .4425532  Pancreatic hyperenzymemia \| -.0436646 .327999 -0.13 0.894 -.6955982 .6082689  Pancreatic neuroendocrine neoplasms \| -.1919534 .2111382 -0.91 0.366 -.6116133 .2277066  Pancreatitis \| -.4232059 .2365583 -1.79 0.077 -.8933911 .0469794  Pancreatoduodenectomy and/or distal pancreatectomy \| -.087674 .1704528 -0.51 0.608 -.4264675 .2511194  Healthy population+Breast cancer \| Reference | | | | | |

| **Supp. Table 6 Meta-regression analyses of potential sources of heterogeneity in fatty pancreas prevalence in the healthy population** | | | | | |
| --- | --- | --- | --- | --- | --- |
| Variables | N studies | Univariate analysis | | Multivariate analysis | |
|  |  | *p* value | R² (%) | p value | R² (%) |
| Multivariate global effect | 55 | *-* | - | < 0.001 | 28.95 |
| Year of publication | 55 | 0.563 | -0.77 |  |  |
| Sample Size | 55 | 0.014 | 11.69 |  |  |
| WHO regions | 55 | 0.677 | -3.51 |  |  |
| Diagnostic modality | 55 | 0.679 | -2.5 |  |  |
| World Bank income level | 55 | 0.332 | 0.71 |  |  |
| Human Development Index | 55 | 0.004 | 19.39 |  |  |
| Country | 55 | 0.120 | 21.55 |  |  |
| Note: Meta-regression analyses were conducted on Freeman–Tukey-transformed prevalence using restricted maximum likelihood with Knapp–Hartung adjustment to explore sources of heterogeneity. Publication year was included as a predefined moderator. Temporal meta-regression in Figure 4 was conducted separately using raw study-level proportions.  Univariate analysis   1. Year of publication   Meta-regression Number of obs = 55  REML estimate of between-study variance tau2 = .02974  % residual variation due to heterogeneity I-squared_res = 97.53%  Proportion of between-study variance explained Adj R-squared = -0.77%  With Knapp-Hartung modification  ---------------------------------------------------------------------------------------------------------------------  _ES \| Coef. Std. Err. t P>\|t\| [95% Conf. Interval]  ----------------+----------------------------------------------------------------------------------------------------  Year of publication \| .0041649 .0071513 0.58 0.563 -.0101788 .0185087  _cons \| -8.091894 14.44666 -0.56 0.578 -37.06823 20.88444  ---------------------------------------------------------------------------------------------------------------------   2. Sample Size   Meta-regression Number of obs = 55  REML estimate of between-study variance tau2 = .02606  % residual variation due to heterogeneity I-squared_res = 97.54%  Proportion of between-study variance explained Adj R-squared = 11.69%  With Knapp-Hartung modification  -----------------------------------------------------------------------------------------------------------  _ES \| Coef. Std. Err. t P>\|t\| [95% Conf. Interval]  -------------+---------------------------------------------------------------------------------------------  Sample Size \| -7.20e-06 2.83e-06 -2.55 0.014 -.0000129 -1.53e-06  _cons \| .3449459 .0260861 13.22 0.000 .2926238 .397268  -----------------------------------------------------------------------------------------------------------   3. WHO regions   Meta-regression Number of obs = 55  REML estimate of between-study variance tau2 = .03055  % residual variation due to heterogeneity I-squared_res = 97.61%  Proportion of between-study variance explained Adj R-squared = -3.51%  Joint test for all covariates Model F(5,49) = 0.63  With Knapp-Hartung modification Prob > F = 0.6766  ----------------------------------------------------------------------------------------------------------  _ES \| Coef. Std. Err. t P>\|t\| [95% Conf. Interval]  -------------+-------------------------------------------------------------------------------------------  African region \| -.1444059 .233777 -0.62 0.540 -.6141984 .3253865  Eastern Mediterranean Region \| -.0338286 .1989654 -0.17 0.866 -.4336645 .3660073  European Region \| .0939929 .1390282 0.68 0.502 -.1853946 .3733805  Region of the Americas \| .1245362 .1629831 0.76 0.448 -.2029907 .4520631  Western Pacific Region \| .0369618 .1375267 0.27 0.789 -.2394086 .3133321  South-East Asia Region \| Reference  _cons \| .2644059 .132127 2.00 0.051 -.0011131 .529925  ---------------------------------------------------------------------------------------------------------   4. Diagnostic modality   Meta-regression Number of obs = 55  REML estimate of between-study variance tau2 = .03025  % residual variation due to heterogeneity I-squared_res = 97.61%  Proportion of between-study variance explained Adj R-squared = -2.50%  Joint test for all covariates Model F(4,50) = 0.58  With Knapp-Hartung modification Prob > F = 0.6788  --------------------------------------------------------------------------------------------------------------------  _ES \| Coef. Std. Err. t P>\|t\| [95% Conf. Interval]  ---------------------+----------------------------------------------------------------------------------------------  CT \| -.0849295 .2075591 -0.41 0.684 -.5018243 .3319652  EUS \| -.1498719 .2124773 -0.71 0.484 -.576645 .2769012  MRI\| -.1952851 .212734 -0.92 0.363 -.6225739 .2320037  TUS \| -.1043854 .2047553 -0.51 0.612 -.5156486 .3068778  HA \| Reference  _cons \| .4387755 .2010066 2.18 0.034 .0350419 .8425091  --------------------------------------------------------------------------------------------------------------------   5. World Bank income level   Meta-regression Number of obs = 55  REML estimate of between-study variance tau2 = .0293  % residual variation due to heterogeneity I-squared_res = 97.72%  Proportion of between-study variance explained Adj R-squared = 0.71%  Joint test for all covariates Model F(2,52) = 1.13  With Knapp-Hartung modification Prob > F = 0.3319  ---------------------------------------------------------------------------------------------------------------------  _ES \| Coef. Std. Err. t P>\|t\| [95% Conf. Interval]  ----------------------+---------------------------------------------------------------------------------------------  High income \| .2096071 .1545336 1.36 0.181 -.1004873 .5197014  Upper middle income \| .1631046 .1535976 1.06 0.293 -.1451115 .4713207  Lower middle income \| Reference  _cons \| .1433667 .1495306 0.96 0.342 -.1566885 .4434219  ------------------------------------------------------------------------------------------------------------------   6. Human Development Index   Meta-regression Number of obs = 55  REML estimate of between-study variance tau2 = .02379  % residual variation due to heterogeneity I-squared_res = 97.45%  Proportion of between-study variance explained Adj R-squared = 19.39%  Joint test for all covariates Model F(2,52) = 6.18  With Knapp-Hartung modification Prob > F = 0.0039  --------------------------------------------------------------------------------------------------------  _ES \| Coef. Std. Err. t P>\|t\| [95% Conf. Interval]  -------------+------------------------------------------------------------------------------------------  High human development \| .0147867 .235669 0.06 0.950 -.4581176 .4876911  Very high human development \| .1945308 .233316 0.83 0.408 -.2736519 .6627135  Low human development \| Reference  _cons \| .1818182 .2316073 0.79 0.436 -.2829359 .6465723  --------------------------------------------------------------------------------------------------------   7. Country   Meta-regression Number of obs = 55  REML estimate of between-study variance tau2 = .02315  % residual variation due to heterogeneity I-squared_res = 95.86%  Proportion of between-study variance explained Adj R-squared = 21.55%  Joint test for all covariates Model F(16,38) = 1.59  With Knapp-Hartung modification Prob > F = 0.1199  -----------------------------------------------------------------------------------------------------------  _ES \| Coef. Std. Err. t P>\|t\| [95% Conf. Interval]  -------------+---------------------------------------------------------------------------------------------  Chile \| -.2449619 .371719 -0.66 0.514 -.9974678 .5075439  China \| -.3611051 .3356013 -1.08 0.289 -1.040494 .3182842  Denmark \| -.4311688 .4017836 -1.07 0.290 -1.244537 .3821995  Egypt \| -.4254546 .3740395 -1.14 0.262 -1.182658 .3317488  Indonesia \| -.2784579 .351878 -0.79 0.434 -.9907976 .4338819  Iran \| -.2866826 .3709952 -0.77 0.444 -1.037723 .4643578  Israel \| -.408372 .3674465 -1.11 0.273 -1.152229 .3354847  Japan \| -.2074604 .3376796 -0.61 0.543 -.891057 .4761362  Korea \| -.0549954 .3392579 -0.16 0.872 -.7417871 .6317964  Netherlands \| -.1721212 .3827611 -0.45 0.655 -.9469806 .6027382  New Zealand \| -.29229 .3818896 -0.77 0.449 -1.065385 .4808051  Norway \| -.2121212 .4447214 -0.48 0.636 -1.112413 .6881703  Pakistan \| -.3636364 .403871 -0.90 0.374 -1.18123 .4539577  Türkiye \| -.1307515 .3349149 -0.39 0.698 -.8087514 .5472483  United Kingdom \| -.3902346 .3519996 -1.11 0.275 -1.102821 .3223514  United States \| -.1267327 .3462489 -0.37 0.716 -.827677 .5742116  Sweden \| Reference  _cons \| .5454546 .3318264 1.64 0.108 -.1262929 1.217202  Multivariate analysis   8. Sample Size+Human Development Index   Meta-regression Number of obs = 55  REML estimate of between-study variance tau2 =.02097  % residual variation due to heterogeneity I-squared_res = 96.76%  Proportion of between-study variance explained Adj R-squared = 28.95%  Joint test for all covariates Model F(3,51) = 6.66  With Knapp-Hartung modification Prob > F = 0.0007  ----------------------------------------------------------------------------------------------------------  _ES \| Coef. Std. Err. t P>\|t\| [95% Conf. Interval]  -------------+--------------------------------------------------------------------------------------------  Sample Size \| -6.31e-06 2.56e-06 -2.47 0.017 -.0000115 -1.18e-06  High human development \| .0451356 .2295023 0.20 0.845 -.4156094 .5058806  Very high human development \| .2117899 .227116 0.93 0.355 -.2441644 .6677443  Low human development \| Reference  _cons \| .1820266 .2254299 0.81 0.423 -.2705428 .6345959 | | | | | |

| **Supp. Table 7 Analyzing the risk of various pancreatic and metabolism-related diseases in patients with fatty pancreas** | | | | | | | | | | |
| --- | --- | --- | --- | --- | --- | --- | --- | --- | --- | --- |
| Variable | Number of  studies | Group | Number of Participants | Prevalence meta | | Risk meta | | | | |
|  |  |  |  | Prevalence  (95% CI)* | *p*^heterogeneity^ (I²) |  | OR (95%CI) | *p*-value | *p*^heterogeneity^ (I²) | *p*^Begg^; *p*^Egger^ |
| Pancreatic adenocarcinoma | 8 | FP | 773 | 42.6% (24.7-61.6) | < 0.001 (96.15%) | 2.12 (1.18-3.81) | | 0.011 | < 0.001 (78.00%) | 0.536; 0.053 |
|  |  | Non-FP | 1,089 | 24.6% (10.7-41.7) | < 0.001 (96.16%) |  |  |  |  |  |
| MASLD/formerly NAFLD | 15 | FP | 6,256 | 65.0% (55.7-73.7) | < 0.001 (97.56%) | 4.95 (2.67-9.19) | | < 0.001 | < 0.001 (98.04%) | 0.113; 0.903 |
|  |  | Non-FP | 46,321 | 29.6% (24.3-35.2) | < 0.001 (98.53%) |  |  |  |  |  |
| Overweight | 4 | FP | 217 | 41.3% (22.9-61.0) | < 0.001 (86.71%) | 1.89 (1.23-2.92) | | 0.004 | 0.303 (17.54%) | 0.308; 0.178 |
|  |  | Non-FP | 518 | 31.6% (25.6-37.8) | 0.153 (43.08%) |  |  |  |  |  |
| Central obesity | 5 | FP | 10,946 | 76.1% (60.3-88.9) | < 0.001 (99.44%) | 4.88 (3.51-6.77) | | < 0.001 | < 0.001 (94.85%) | 1.000; 0.245 |
|  |  | Non-FP | 70,226 | 40.4% (26.7-54.9) | < 0.001 (99.91%) |  |  |  |  |  |
| Type 2 diabetes | 12 | FP | 1,992 | 35.2% (21.6-50.1) | < 0.001 (97.68%) | 3.81 (2.11-6.87) | | < 0.001 | < 0.001 (92.75%) | 0.631; 0.974 |
|  |  | Non-FP | 7,322 | 13.4% (7.7-20.4) | < 0.001 (97.80%) |  |  |  |  |  |
| MASLD metabolic dysfunction-associated steatotic liver disease; NAFLD, nonalcoholic fatty liver disease; FP, fatty pancreas; OR, odds ratio; CI, confidence interval. | | | | | | | | | | |

| **Supp. Table 8 Diagnostic criteria for overweight, obesity, and central obesity** | | |
| --- | --- | --- |
| Author | Diagnostic criteria | |
| Berger et al, 2023^51^ | Overweight: BMI 25-29.9 kg/m² | |
| Kirsoy et al, 2024^31^ | Overweight: For females, those with a circumference between 90 cm and less than 105 cm were classified as overweight; For males, a waist circumference between 100 cm and less than 110 cm were overweight | |
| Qureshi et al, 2022^6^ | Overweight: N/A | |
| Gaujoux et al, 2012^60^ | Overweight: BMI 25-29.9 kg/m² | |
| Sbeit et al, 2021^18^ | Obesity: N/A | |
| Wang et al, 2018^81^ | Obesity: BMI ≥28 kg/m^2^ | |
| Wang et al, 2014^121^ | Obesity: BMI ≥25 kg/m^2^ | |
| Bi et al, 2022^69^ | Obesity: BMI ≥25 kg/m^2^ | |
| Berger et al, 2023^51^ | Obesity: BMI ≥30 kg/m^2^ | |
| Cho et al, 2022^115^ | Obesity: defined as a BMI greater than or equal to 95th percentile for age and sex | |
| Gaujoux et al, 2012^60^ | Obesity: BMI ≥30 kg/m^2^ | |
| Kirsoy et al, 2024^31^ | Obesity: For females, those with a circumference equal to or greater than 105 cm were classified as obese. For males, a waist circumference equal to or greater than 110 cm were classified as obese. | |
| Sbeit et al, 2023^17^ | Obesity: N/A | |
| Han et al, 2023 | Obesity: N/A | |
| Wang et al, 2018^81^ | Central obesity: defined as a male AC ≥90 cm or a female AC ≥80 cm | |
| Weng et al, 2018^70^ | Central obesity: defined as Chinese men with a waist ≥85 cm, or women with a waist ≥80 cm. | |
| Hung et al, 2018^76^ | Central obesity: defined as waist circumference of 90 cm or greater in men and 80 cm or greater in women | |
| Dong et al, 2024^47^ | Central obesity: defined as meeting one of the following criteria: for males, waist circumference ≥90 cm or waist-to-hip ratio ≥0.9, or waist-to-height ratio ≥ 0.5, and for females, waist circumference ≥80 cm or waist-to-hip ratio ≥0.85, or waist-to-height ratio ≥0.5 | |
| Chan et al, 2022^120^ | Central obesity: defined as a waist circumference of 90 or more cm in men and 80 cm or more in women | |

**Supp. Table 9. Restricted sensitivity analyses of fatty pancreas prevalence according to population selection**

| Analysis | Number of studies | Number of participants | Pooled prevalence (95% CI) | I² |
| --- | --- | --- | --- | --- |
| Overall study population | 119 | 152,684 | 42.3% (38.4–46.2) | 99.48% |
| Excluding pancreatic disease or pancreatic surgical populations | 89 | 147,922 | 41.7% (37.4–46.1) | 99.57% |
| Excluding metabolic high-risk populations | 96 | 148,666 | 35.1% (31.4–39.0) | 99.47% |
| Excluding both pancreatic disease/surgical and metabolic high-risk populations | 65 | 143,904 | 31.1% (27.0–35.5) | 99.59% |
| Healthy population | 55 | 141,416 | 30.5% (26.1–35.1) | 99.62% |

Pancreatic disease or pancreatic surgical populations included cohorts selected for pancreatic diseases, pancreatic neoplasms, or pancreatic resection procedures according to the population classifications used in the original studies.

Metabolic high-risk populations included cohorts selected for overweight or obesity, MASLD/NAFLD, prediabetes or diabetes mellitus, obstructive sleep apnoea, polycystic ovary syndrome, or other predefined metabolic-risk conditions.

When an original study reported more than one population, only the cohort meeting the exclusion criterion was removed, and other eligible cohorts from the same study were retained.

Pooled prevalence and 95% confidence intervals were estimated using the same random-effects model and Freeman–Tukey double-arcsine transformation as in the primary analysis.

**References of included studies:**

1.	Elhady M, Elazab A, Bahagat KA, Abdallah NA, Ibrahim GE. Fatty pancreas in relation to insulin resistance and metabolic syndrome in children with obesity. *J Pediatr Endocrinol Metab* 2019; **32**(1): 19-26.

2.	Hamed DH, Ibrahim RS, Hakim MA, Mohsen M. Insight into clinical, laboratory, and GIT ultrasound diagnostic findings of cystic fibrosis in pediatrics. *Egyptian Pediatric Association Gazette* 2022; **70**(1).

3.	Okasha HH, Hegazy MA, Shaker O, et al. Study of non-alcoholic fatty pancreatic disease among the Egyptian population and the value of serum fatty acid binding protein-1 (FABP-1) as a non-invasive biomarker. *Clin Res Hepatol Gastroenterol* 2024; **48**(6): 102364.

4.	Osman MAA, Alkhouly M, Elmohaseb GF, et al. Relation Between Non-Alcoholic Fatty Pancreas and Clinical and Biochemical Parameters in Women with Polycystic Ovary Syndrome: A Multi-Centric Study. *International Journal of General Medicine* 2022; **15**: 8225-33.

5.	Naguib MN, Kassem GK, Mansour LO, Abdelaziz AA, Asal FE. Study of Fatty Pancreas in Egyptian Patients with Non Alcoholic Fatty Liver Disease: A Cross Sectional Study. *African Journal of Biological Sciences (South Africa)* 2024; **6**(14): 6262-74.

6.	Qureshi S, Memon F, Chisti N, Ghazanfar S, Quraishy MS. Frequency of Nonalcoholic fatty pancreatic disease in patients with carcinoma pancreas presenting for upper abdominal Endoscopic Ultrasound in a tertiary care center. *Journal of the Pakistan Medical Association* 2022; **72**(11): 2209-12.

7.	Noun R, Riachy E, Ghorra C, et al. The impact of obesity on surgical outcome after pancreaticoduodenectomy. *Jop* 2008; **9**(4): 468-76.

8.	Sotoudehmanesh R, Tahmasbi A, Sadeghi A, Hosseini H, Mohamadnejad M. The Prevalence of Nonalcoholic Fatty Pancreas by Endoscopic Ultrasonography. *Pancreas* 2019; **48**(9): 1220-4.

9.	Blaho M, Macháčková J, Dítě P, et al. Use of Magnetic Resonance Imaging to Quantify Fat and Steatosis in the Pancreas in Patients after Bariatric Surgery: a Retrospective Study. *Obesity Surgery* 2022; **32**(11): 3666-74.

10.	Gjela M, Askeland A, Mellergaard M, Drewes AM, Handberg A, Frøkjær JB. Intra-pancreatic fat deposition and its relation to obesity: a magnetic resonance imaging study. *Scandinavian Journal of Gastroenterology* 2024.

11.	Previtali C, Sartoris R, Rebours V, et al. Quantitative imaging predicts pancreatic fatty infiltration on routine CT examination. *Diagnostic and Interventional Imaging* 2023; **104**(7-8): 359-67.

12.	Rebours V, Gaujoux S, D'Assignies G, et al. Obesity and fatty pancreatic infiltration are risk factors for pancreatic precancerous lesions (PanIN). *Clinical Cancer Research* 2015; **21**(15): 3522-8.

13.	Tranchart H, Gaujoux S, Rebours V, et al. Preoperative CT scan helps to predict the occurrence of severe pancreatic fistula after pancreaticoduodenectomy. *Ann Surg* 2012; **256**(1): 139-45.

14.	Gaujoux S, Cortes A, Couvelard A, et al. Fatty pancreas and increased body mass index are risk factors of pancreatic fistula after pancreaticoduodenectomy. *Surgery* 2010; **148**(1): 15-23.

15.	Rosso E, Casnedi S, Pessaux P, et al. The role of "Fatty Pancreas" and of BMI in the occurrence of pancreatic fistula after pancreaticoduodenectomy. *Journal of Gastrointestinal Surgery* 2009; **13**(10): 1845-51.

16.	Harrell KN, Jajja MR, Postlewait LM, et al. Influence of margin histology on development of pancreatic fistula following pancreatoduodenectomy. *J Surg Res* 2020; **246**: 315-24.

17.	Sbeit W, Abu Elheja F, Msheiil B, et al. Fatty pancreas was associated with a higher acute pancreatitis Systemic Inflammatory Response Syndrome score at hospital admission. *European Journal of Gastroenterology and Hepatology* 2023; **35**(9): 980-4.

18.	Sbeit W, Greener T, Kadah A, et al. Pancreatic and hepatobiliary manifestations of nonalcoholic fatty pancreatic disease: A referral multi-center experience. *European Journal of Gastroenterology and Hepatology* 2021; **33**(1): E297-E301.

19.	Gullo L, Salizzoni E, Serra C, Calculli L, Bastagli L, Migliori M. Can pancreatic steatosis explain the finding of pancreatic hyperenzymemia in subjects with dyslipidemia? *Pancreas* 2006; **33**(4): 351-3.

20.	Angrisani M, Ceresoli M, Ippolito D, et al. Estimating Fatty Pancreas - A Preoperative Bedside Assessment by Bioelectric Impedance Analysis: Implications for Pancreatic Surgery. *Pancreas* 2022; **51**(4): 345-50.

21.	Della Corte C, Mosca A, Majo F, et al. Nonalcoholic fatty pancreas disease and Nonalcoholic fatty liver disease: More than ectopic fat. *Clinical Endocrinology* 2015; **83**(5): 656-62.

22.	Mak AL, Wassenaar N, van Dijk AM, et al. Intrapancreatic fat deposition is unrelated to liver steatosis in metabolic dysfunction-associated steatotic liver disease. *JHEP Reports* 2024; **6**(3).

23.	Engjom T, Kavaliauskiene G, Tjora E, et al. Sonographic pancreas echogenicity in cystic fibrosis compared to exocrine pancreatic function and pancreas fat content at Dixon-MRI. *PLoS ONE* 2018; **13**(7).

24.	Marti-Aguado D, Ten-Esteve A, Baracaldo-Silva CM, et al. Pancreatic steatosis and iron overload increases cardiovascular risk in non-alcoholic fatty liver disease. *Frontiers in Endocrinology* 2023; **14**.

25.	Maetzel H, Rutkowski W, Panic N, et al. Non-alcoholic fatty pancreas disease and pancreatic exocrine insufficiency: pilot study and systematic review. *Scandinavian Journal of Gastroenterology* 2023; **58**(9): 1030-7.

26.	Sezgin O, Yaraş S, Cindoruk M, et al. Prevalence of Pancreatic Steatosis and Its Associated Factors in Turkey: A Nation-Wide Multicenter Study. *Turkish Journal of Gastroenterology* 2024; **35**(3): 239-54.

27.	Abbasoğlu A, Karçaaltıncaba M, Karaosmanoğlu AD, Özmen MN, Akata D, İdilman İ S. Associations Between Hepatic and Pancreatic Steatosis with Lumbar Spinal Bone Marrow Fat: A Single-Center Magnetic Resonance Imaging Study. *Turk J Gastroenterol* 2023; **34**(6): 618-25.

28.	Ozturk K, Dogan T, Celikkanat S, et al. The association of fatty pancreas with subclinical atherosclerosis in nonalcoholic fatty liver disease. *European Journal of Gastroenterology and Hepatology* 2018; **30**(4): 411-7.

29.	Uygun A, Kadayifci A, Demirci H, et al. The effect of fatty pancreas on serum glucose parameters in patients with nonalcoholic steatohepatitis. *Eur J Intern Med* 2015; **26**(1): 37-41.

30.	Gursoy Coruh A, Uzun C, Akkaya Z, Halil Elhan A. The relation of CT quantified pancreatic fat index with visceral adiposity and hepatic steatosis. *Turk J Surg* 2020; **36**(3): 241-8.

31.	Kirsoy F, Yalniz M, Bahçecioğlu İ, et al. The gut-pancreas axis: investigating the relationship between microbiota metabolites and pancreatic steatosis. *Internal and emergency medicine* 2024.

32.	Koc U, Taydas O. Investigation of the Relationship between Fatty Pancreas and Cholecystectomy Using Noncontrast Computed Tomography. *Journal of Medical Imaging and Radiation Sciences* 2019; **50**(2): 220-6.

33.	Koç U, Taydaş O. Evaluation of pancreatic steatosis prevalence and anthropometric measurements using non-contrast computed tomography. *Turkish Journal of Gastroenterology* 2020; **31**(9): 640-8.

34.	Doğan H, Uzer E, Esengür Ö T, Hassoy H, Güneyli S. Relationship between hepatic and pancreatic steatosis and the COVID-19 pneumonia total severity score and prognosis with an emphasis on prognostic strength. *Diagn Interv Radiol* 2023; **29**(4): 363-70.

35.	Ahbab S, Ünsal A, Ataoğlu HE, Can TS, Kayaş D, Savaş Y. Prediabetes and Type 2 Diabetes are Independent Risk Factors for Computed Tomography-Estimated Nonalcoholic Fatty Pancreas Disease. *Clinics (Sao Paulo, Brazil)* 2019; **74**: e1337.

36.	Altinmakas E, Guler B, Copur S, et al. Determinants of pancreatic steatosis: A retrospective observational study. *Middle East Journal of Digestive Diseases* 2021; **13**(4): 343-9.

37.	Ulasoglu C, Tekin ZN, Akan K, Yavuz A. Does nonalcoholic pancreatic steatosis always correlate with nonalcoholic fatty liver disease? *Clinical and Experimental Gastroenterology* 2021; **14**: 269-75.

38.	Bayyigit A, Gokden Y, Onol S, Ozek FZ, Saglam S, Adas M. Hypothyroidism and subclinical hypothyroidism are associated with fatty pancreas (Non-Alcoholic Fatty Pancreas Disease). *Diabetes/Metabolism Research and Reviews* 2024; **40**(2).

39.	Hasbal NB, Copur S, Peltek IB, et al. Pancreatic steatosis is an independent risk factor for post-transplant diabetes mellitus in kidney transplant patients. *Clinical Transplantation* 2024; **38**(1).

40.	Guneyli S, Dogan H, Esengur OT, Hassoy H. Computed tomography evaluation of pancreatic steatosis: Correlation with COVID-19 prognosis. *Future Virology* 2022; **17**(4): 231-7.

41.	Öz A, Akçalar S. Increased Incidence of Pancreatic Steatosis Detected Using Computed Tomography at Initial Diagnosis of Coronavirus Disease 2019. *Turkish Journal of Gastroenterology* 2023; **34**(3): 270-7.

42.	Sezgin O, Yaraş S, Özdoğan O. Pancreatic Steatosis Is Associated with Both Metabolic Syndrome and Pancreatic Stiffness Detected by Ultrasound Elastography. *Digestive Diseases and Sciences* 2022; **67**(1): 293-304.

43.	Sahin S, Yerlikaya MG, Ozderya A, et al. Non-Alcoholic Fatty Pancreas Disease is Associated with SYNTAX Score in Acute Coronary Syndrome. *Acta Cardiologica Sinica* 2022; **38**(6): 683-90.

44.	Sahin S, Karadeniz A. Pancretic Fat Accummulation is Associated with Subclinical Atherosclerosis. *Angiology* 2022; **73**(6): 508-13.

45.	Kul S, Karadeniz A, Dursun İ, et al. Non-Alcoholic Fatty Pancreas Disease is Associated with Increased Epicardial Adipose Tissue and Aortic Intima-Media Thickness. *Acta Cardiol Sin* 2019; **35**(2): 118-25.

46.	Diamond C, Pansini M, Hamid A, et al. Quantitative Imaging Reveals Steatosis and Fibroinflammation in Multiple Organs in People With Type 2 Diabetes: A Real-World Study. *Diabetes* 2024; **73**(8): 1285-99.

47.	Dong X, Zhu Q, Yuan C, et al. Associations of Intrapancreatic Fat Deposition With Incident Diseases of the Exocrine and Endocrine Pancreas: A UK Biobank Prospective Cohort Study. *Am J Gastroenterol* 2024; **119**(6): 1158-66.

48.	Halle-Smith JM, Vinuela E, Brown RM, et al. A comparative study of risk factors for pancreatic fistula after pancreatoduodenectomy or distal pancreatectomy. *HPB (Oxford)* 2017; **19**(8): 727-34.

49.	Karasu S, Gungor F, Onak C, Dilek ON. Relation of computed tomography features of the pancreatic tissue and development of pancreatic fistula after pancreaticoduodenectomy. *Clinical Imaging* 2021; **72**: 114-9.

50.	Ozbulbul NI, Yurdakul M, Tola M. Does the visceral fat tissue show better correlation with the fatty replacement of the pancreas than with BMI? *Eurasian J Med* 2010; **42**(1): 24-7.

51.	Berger Z, Orellana F, Cocio R, et al. Pancreatic steatosis: A frequent finding in a Chilean population. *Revista de Gastroenterologia de Mexico* 2023; **88**(2): 118-24.

52.	Swauger SE, Fashho K, Hornung LN, et al. Association of pancreatic fat on imaging with pediatric metabolic co-morbidities. *Pediatric Radiology* 2023; **53**(10): 2030-9.

53.	Trout AT, Hunte DE, Mouzaki M, et al. Relationship between abdominal fat stores and liver fat, pancreatic fat, and metabolic comorbidities in a pediatric population with non-alcoholic fatty liver disease. *Abdom Radiol (NY)* 2019; **44**(9): 3107-14.

54.	Hoogenboom SA, Bolan CW, Chuprin A, et al. Pancreatic steatosis on computed tomography is an early imaging feature of pre-diagnostic pancreatic cancer: A preliminary study in overweight patients. *Pancreatology* 2021; **21**(2): 428-33.

55.	Mathur A, Hernandez J, Shaheen F, et al. Preoperative computed tomography measurements of pancreatic steatosis and visceral fat: Prognostic markers for dissemination and lethality of pancreatic adenocarcinoma. *HPB* 2011; **13**(6): 404-10.

56.	Pham YH, Bingham BA, Bell CS, et al. Prevalence of pancreatic steatosis at a pediatric tertiary care center. *Southern Medical Journal* 2016; **109**(3): 196-8.

57.	Krill JT, Szafron D, Elhanafi S, et al. Endoscopic Ultrasound Finding of Diffuse Echogenicity in the Pancreas, Is It Relevant? *Digestive Diseases and Sciences* 2022; **67**(7): 3244-51.

58.	Sepe PS, Ohri A, Sanaka S, et al. A prospective evaluation of fatty pancreas by using EUS. *Gastrointestinal Endoscopy* 2011; **73**(5): 987-93.

59.	Rosenblatt R, Mehta A, Snell D, Hissong E, Kierans AS, Kumar S. Ultrasonographic Nonalcoholic Fatty Pancreas Is Associated with Advanced Fibrosis in NAFLD: A Retrospective Analysis. *Digestive Diseases and Sciences* 2019; **64**(1): 262-8.

60.	Gaujoux S, Torres J, Olson S, et al. Impact of obesity and body fat distribution on survival after pancreaticoduodenectomy for pancreatic adenocarcinoma. *Annals of Surgical Oncology* 2012; **19**(9): 2908-16.

61.	Becetti I, Lopez Lopez AP, Galbiati F, et al. Changes in pancreatic steatosis by computed tomography 24 months after sleeve gastrectomy in youth with severe obesity. *Surg Obes Relat Dis* 2024.

62.	Patel AC, Yagnik VD. Evaluation of risk factors for postoperative pancreatic fistula following pancreaticoduodenectomy. *Formosan Journal of Surgery* 2019; **52**(3): 76-83.

63.	Lesmana CR, Pakasi LS, Inggriani S, Aidawati ML, Lesmana LA. Prevalence of Non-Alcoholic Fatty Pancreas Disease (NAFPD) and its risk factors among adult medical check-up patients in a private hospital: a large cross sectional study. *BMC Gastroenterol* 2015; **15**: 174.

64.	Lesmana CRA, Gani RA, Lesmana LA. Non-alcoholic fatty pancreas disease as a risk factor for pancreatic cancer based on endoscopic ultrasound examination among pancreatic cancer patients: A single-center experience. *JGH Open* 2018; **2**(1): 4-7.

65.	Sun P, Fan C, Wang R, et al. Computed tomography-estimated pancreatic steatosis is associated with carotid plaque in type 2 diabetes mellitus patients: A cross-sectional study from China. *Diabetes, Metabolic Syndrome and Obesity* 2021; **14**: 1329-37.

66.	Bai L, Ma M, Lin M, et al. Abdominal magnetic resonance imaging assessment and quantification of pancreatic and liver adipose tissues in obesity before and after laparoscopic sleeve gastrectomy. *Journal of Radiation Research and Applied Sciences* 2023; **16**(3).

67.	Chen Z, Wang H, Jiang T. Value of Two-Dimensional Shear-Wave Elastography in Differentiating Pancreatic Steatosis From Pancreatic Fibrosis. *J Ultrasound Med* 2024.

68.	Chiyanika C, Chan DFY, Hui SCN, et al. The relationship between pancreas steatosis and the risk of metabolic syndrome and insulin resistance in Chinese adolescents with concurrent obesity and non-alcoholic fatty liver disease. *Pediatric Obesity* 2020; **15**(9).

69.	Bi Y, Lin HY, Li ML, Zhou J, Sun XL. The Association between Pancreatic Steatosis and Metabolic Syndrome: A 5-Year Follow-up Study among a General Chinese Population. *Pancreas* 2022; **51**(8): 1000-6.

70.	Weng S, Zhou J, Chen X, Sun Y, Mao Z, Chai K. Prevalence and factors associated with nonalcoholic fatty pancreas disease and its severity in China. *Medicine (United States)* 2018; **97**(26).

71.	Hong C, Yan Y, Su L, Chen D, Zhang C. Development of a risk-stratification scoring system for predicting risk of breast cancer based on non-alcoholic fatty liver disease, non-alcoholic fatty pancreas disease, and uric acid. *Open Medicine (Poland)* 2022; **17**(1): 619-25.

72.	Ke H, Lin J, Han L, Lyu G, Li S. Predictive model for pancreatic steatosis after neoadjuvant chemotherapy in breast cancer: Construction and ultrasound validation. *Journal of Radiation Research and Applied Sciences* 2024; **17**(2).

73.	Xiao Y, Wang H, Han L, Huang Z, Lyu G, Li S. Predictive value of anthropometric and biochemical indices in non-alcoholic fatty pancreas disease: a cross-sectional study. *BMJ Open* 2024; **14**(4).

74.	Li Y, Yang J, Fan Z, et al. Pancreatic steatosis and its correlation with clinicopathological features in patients with pancreatic neuroendocrine neoplasms. *Journal of Pancreatology* 2023; **6**(2): 48-54.

75.	Chen Y, Zhang P, Lv S, et al. Ectopic fat deposition and its related abnormalities of lipid metabolism followed by nonalcoholic fatty pancreas. *Endoscopic Ultrasound* 2022; **11**(5): 407-13.

76.	Hung CS, Tseng PH, Tu CH, et al. Increased Pancreatic Echogenicity with US: Relationship to Glycemic Progression and Incident Diabetes. *Radiology* 2018; **287**(3): 853-63.

77.	Luo PJ, Chang CC, Liu KL, Chang YC. Cross-Sectional Comparison of Magnetic Resonance Imaging and Ultrasound on the Quantitative Assessment of Pancreatic Fat. *Journal of Radiological Science* 2023; **48**(1).

78.	Wen Y, Chen C, Kong X, et al. Pancreatic fat infiltration, β-cell function and insulin resistance: A study of the young patients with obesity. *Diabetes Res Clin Pract* 2022; **187**: 109860.

79.	Xingjun G, Feng Z, Meiwen Y, et al. A score model based on pancreatic steatosis and fibrosis and pancreatic duct diameter to predict postoperative pancreatic fistula after Pancreatoduodenectomy. *BMC surgery* 2019; **19**(1): 75.

80.	Li C, Chen X, Zhu X, Cao M, Tang Q, Wu W. MRI-Measured Pancreatic Fat Correlates with Increased Arterial Stiffness in Patients Who are Overweight and Obese. *Diabetes Metab Syndr Obes* 2024; **17**: 2283-91.

81.	Wang D, Yu XP, Xiao WM, et al. Prevalence and clinical characteristics of fatty pancreas in Yangzhou, China: A cross-sectional study. *Pancreatology* 2018; **18**(3): 263-8.

82.	Dei H, Natsume S, Okuno M, et al. Impact of pancreatic fat infiltration on postoperative pancreatic fistula occurrence in patients undergoing invagination pancreaticojejunostomy. *HPB* 2022; **24**(12): 2119-24.

83.	Tanaka K, Yamada S, Sonohara F, et al. Pancreatic Fat and Body Composition Measurements by Computed Tomography are Associated with Pancreatic Fistula After Pancreatectomy. *Annals of Surgical Oncology* 2021; **28**(1): 530-8.

84.	Kuriyama T, Ishibashi C, Kozawa J, et al. Effects of liraglutide on intrapancreatic fat deposition in patients with type 2 diabetes. *Clinical Nutrition ESPEN* 2024; **59**: 208-13.

85.	Okada K, Watahiki T, Horie K, et al. The prevalence and clinical implications of pancreatic fat accumulation identified during a medical check-up. *Medicine (Baltimore)* 2021; **100**(41): e27487.

86.	Mandai K, Uno K, Nakase K, Kawamura T, Yasuda K. Association between hyperechogenic pancreas and pancreatic ductal adenocarcinoma concomitant with intraductal papillary mucinous neoplasms. *Journal of Medical Ultrasonics* 2019; **46**(4): 435-9.

87.	Miura S, Kume K, Kikuta K, et al. Focal Parenchymal Atrophy and Fat Replacement Are Clues for Early Diagnosis of Pancreatic Cancer with Abnormalities of the Main Pancreatic Duct. *Tohoku J Exp Med* 2020; **252**(1): 63-71.

88.	Kanno A, Masamune A, Hanada K, et al. Multicenter study of early pancreatic cancer in Japan. *Pancreatology* 2018; **18**(1): 61-7.

89.	Silman C, Matsumoto S, Mori H, et al. Evaluation of the fat plane of the pancreatic groove using multidetector CT. *Clin Radiol* 2018; **73**(10): 910.e7-.e13.

90.	Fukuda Y, Yamada D, Eguchi H, et al. CT Density in the Pancreas is a Promising Imaging Predictor for Pancreatic Ductal Adenocarcinoma. *Ann Surg Oncol* 2017; **24**(9): 2762-9.

91.	Ookura R, Usuki N. Visual assessment of pancreatic fat deposition: useful grading system and the relation to BMI and diabetes. *Japanese Journal of Radiology* 2023; **41**(2): 172-9.

92.	Tomita Y, Azuma K, Nonaka Y, et al. Pancreatic fatty degeneration and fibrosis as predisposing factors for the development of pancreatic ductal adenocarcinoma. *Pancreas* 2014; **43**(7): 1032-41.

93.	Sano S, Okamura Y, Ohgi K, et al. Histological pancreatic findings correlate with computed tomography attenuation and predict postoperative pancreatic fistula following pancreatoduodenectomy. *HPB (Oxford)* 2022; **24**(9): 1519-26.

94.	Ishibashi C, Kozawa J, Hosakawa Y, et al. Pancreatic fat is related to the longitudinal decrease in the increment of C-peptide in glucagon stimulation test in type 2 diabetes patients. *Journal of Diabetes Investigation* 2020; **11**(1): 80-7.

95.	Fujii M, Ohno Y, Yamada M, Kamada Y, Miyoshi E. Impact of fatty pancreas and lifestyle on the development of subclinical chronic pancreatitis in healthy people undergoing a medical checkup. *Environmental Health and Preventive Medicine* 2019; **24**(1).

96.	Horii T, Kozawa J, Fujita S, et al. Amelioration of pancreatic fat accumulation in Japanese type 2 diabetes patients treated with sodium-glucose cotransporter 2 inhibitors: a retrospective study. *Obesity Science and Practice* 2021; **7**(3): 346-52.

97.	Hori M, Takahashi M, Hiraoka N, et al. Association of pancreatic fatty infiltration with pancreatic ductal adenocarcinoma. *Clinical and Translational Gastroenterology* 2014; **5**.

98.	Kawamura A, Takakura K, Torisu Y, et al. Impact of qualitative endoscopic ultrasonography on fatty pancreas at a referral medical center. *JGH Open* 2022; **6**(1): 44-9.

99.	Otsuka N, Shimizu K, Taniai M, Tokushige K. Risk factors for fatty pancreas and effects of fatty infiltration on pancreatic cancer. *Frontiers in Physiology* 2023; **14**.

100.	Kobashi Y, Uchiyama M, Matsui J. The "K-Sign"-A Novel CT Finding Suggestive before the Appearance of Pancreatic Cancer. *Cancers (Basel)* 2021; **13**(16).

101.	Matsumoto N, Kumagawa M, Saito K, et al. Correlation between pathology and quantitative ultrasonographic evaluation of pancreatic fat with ultrasonographic classification. *Journal of Medical Ultrasonics* 2024; **51**(2): 301-9.

102.	Kobayashi N, Shinohara H, Haruta S, Udagawa H, Ueno M. Reducing the risk of postoperative pancreatic fistula in radical gastrectomy: pre-assessment with computed tomography for the diagnosis of pancreatic steatosis. *Langenbeck's Archives of Surgery* 2022; **407**(2): 587-96.

103.	Makino N, Shirahata N, Honda T, et al. Pancreatic hyperechogenicity associated with hypoadiponectinemia and insulin resistance: A Japanese population study. *World J Hepatol* 2016; **8**(33): 1452-8.

104.	Choi CW, Kim GH, Kang DH, et al. Associated factors for a hyperechogenic pancreas on endoscopic ultrasound. *World J Gastroenterol* 2010; **16**(34): 4329-34.

105.	Park CH, Chung MJ, Park DH, Min S, Park SW. Impact of pancreatic fat on the risk of post-endoscopic retrograde cholangiopancreatography pancreatitis. *Surgical Endoscopy* 2022; **36**(8): 5734-42.

106.	Chung MJ, Park SW, Lee KJ, et al. Clinical impact of pancreatic steatosis measured by CT on the risk of post-ERCP pancreatitis: a multicenter prospective trial. *Gastrointestinal Endoscopy* 2024; **99**(2): 214-23.e4.

107.	Kang BK, Kim M, Shin SJ, Kim YJ. Correlation of Clinical and Histopathologic Parameters with Ultrasonographic Grades in Pediatric Non-Alcoholic Fatty Liver Disease. *Journal of Korean medical science* 2019; **34**(47): e298.

108.	An Y, Yun SJ, Yang IH, Kim DH, Yang DM. Predictive Performance of Ultrasound-Determined Non-Alcoholic Fatty Pancreas Disease Severity for Intermediate and High Risk of Coronary Heart Disease. *Journal of the Korean Society of Radiology* 2019; **80**(6): 1190.

109.	Chang YR, Kang JS, Jang JY, et al. Prediction of Pancreatic Fistula After Distal Pancreatectomy Based on Cross-Sectional Images. *World J Surg* 2017; **41**(6): 1610-7.

110.	Oh H, Park HJ, Oh J, et al. Hyperechoic pancreas on ultrasonography: an analysis of its severity and clinical implications. *Ultrasonography* 2022; **41**(2): 335-43.

111.	Lee JS, Kim SH, Jun DW, et al. Clinical implications of fatty pancreas: Correlations between fatty pancreas and metabolic syndrome. *World Journal of Gastroenterology* 2009; **15**(15): 1869-75.

112.	Hong JJ, Park HJ, Lee ES, Kim MJ. Severity of hyperechoic pancreas on preoperative ultrasonography: high potential as a clinically useful predictor of a postoperative pancreatic fistula. *Ultrasonography* 2024; **43**(4): 272-83.

113.	Singh RG, Nguyen NN, Cervantes A, Kim JU, Stuart CE, Petrov MS. Circulating levels of lipocalin-2 are associated with fatty pancreas but not fatty liver. *Peptides* 2019; **119**.

114.	Skudder-Hill L, Coffey S, Sequeira-Bisson IR, Ko J, Poppitt SD, Petrov MS. Comprehensive analysis of dyslipidemia states associated with fat in the pancreas. *Diabetes and Metabolic Syndrome: Clinical Research and Reviews* 2023; **17**(11).

115.	Cho JY, You SK, Lim HH, Kim HJ. Clinical Significance of Pancreatic Fat in Children: A Single-Center Experience. *Pancreas* 2022; **51**(8): 972-5.

116.	Wong VW, Wong GL, Yeung DK, et al. Fatty pancreas, insulin resistance, and β-cell function: a population study using fat-water magnetic resonance imaging. *Am J Gastroenterol* 2014; **109**(4): 589-97.

117.	Sun Y, Zhang L, Huang JQ, Su J, Cui LG. Non-invasive diagnosis of pancreatic steatosis with ultrasound images using deep learning network. *Heliyon* 2024; **10**(17): e37580.

118.	Lin J, Wang H, Chen X, et al. Obstructive sleep apnoea is independently associated with non-alcoholic fatty pancreatic disease. *Scand J Gastroenterol* 2024: 1-7.

119.	Chan CH, Chang CC, Peng YC. The Clinical Significance of Pancreatic Steatosis in Pancreatic Cancer: A Hospital-Based Study. *Diagnostics (Basel)* 2024; **14**(19).

120.	Chan TT, Tse YK, Lui RN, et al. Fatty Pancreas Is Independently Associated With Subsequent Diabetes Mellitus Development: A 10-Year Prospective Cohort Study. *Clin Gastroenterol Hepatol* 2022; **20**(9): 2014-22.e4.

121.	Wang CY, Ou HY, Chen MF, Chang TC, Chang CJ. Enigmatic ectopic fat: Prevalence of nonalcoholic fatty pancreas disease and its associated factors in a chinese population. *Journal of the American Heart Association* 2014; **3**(1).

122.	Al-Haddad M, Khashab M, Zyromski N, et al. Risk factors for hyperechogenic pancreas on endoscopic ultrasound: A case-control study. *Pancreas* 2009; **38**(6): 672-5.

123.	Han F, Yin L, Yu X, et al. High circulating fibroblast growth factor-21 levels as a screening marker in fatty pancreas patients. *PeerJ* 2023; **11**.

124.	Tahtacı M, Algın O, Karakan T, et al. Can pancreatic steatosis affect exocrine functions of pancreas? *Turk J Gastroenterol* 2018; **29**(5): 588-94.

**Supp. Figure 1 Forest plot of fatty pancreas prevalence in the overall study population**

**Supp. Figure 2** **Forest plot of fatty pancreas prevalence in the overall study population by sex**

**Supp. Figure 3** **Forest plot of fatty pancreas prevalence in the overall study population** **by WHO region**

**Supp. Figure 4 Forest plot of fatty pancreas prevalence in the overall study population across different countries**

**Supp. Figure 5 Forest plot of fatty pancreas prevalence in the overall study population by World Bank income levels**

**Supp. Figure 6 Forest plot of fatty pancreas prevalence in the overall study population** **by Human Development Index levels**

**Supp. Figure 7 Forest plot of fatty pancreas prevalence in the overall study population by diagnostic modalities**

CT, computed tomography; TUS, transabdominal ultrasonography; EUS, endoscopic ultrasound; MRI, magnetic resonance imaging; HA, histology assessment

**Supp. Figure 8 Forest plot of fatty pancreas prevalence across different populations**

IPMN, Intraductal papillary mucinous neoplasms; NAFLD, nonalcoholic fatty liver disease

**Supp. Figure 9 Forest plot of fatty pancreas prevalence in the healthy population by sex**

**Supp. Figure 10 Forest plot of fatty pancreas prevalence in the healthy population by age at diagnosis**

**Supp. Figure 11 Forest plot of fatty pancreas prevalence in the healthy population by WHO region**

**Supp. Figure 12 Forest plot of fatty pancreas prevalence in the healthy population across different countries**

**Supp. Figure 13 Forest plot of fatty pancreas prevalence in the healthy population by World Bank income levels**

**Supp. Figure 14 Forest plot of fatty pancreas prevalence in the healthy population by Human Development Index levels**

**Supp. Figure 15 Forest plot of fatty pancreas prevalence in the healthy population by diagnostic modalities**

MRI, Magnetic resonance imaging; TUS, Transabdominal ultrasonography; HA, Histology assessment; CT, Computed Tomography; EUS, Endoscopic ultrasound**.**

**Supp. Figure 16 Forest plot of fatty pancreas prevalence in the overall study population by publication period**

**Supp. Figure 17 Forest plot of fatty pancreas prevalence in the healthy population by publication period**

**Supp. Figure 18 Normality histogram for the FP prevalence in the overall study population**

**Supp. Figure 19** **Normality Q-Q plot for the FP prevalence in the overall study population**

**Supp. Figure 20 Normality histogram for the FP prevalence in healthy population**

**Supp. Figure 21** **Normality Q-Q plot for the FP prevalence in healthy population**

**Supp. Figure 22** **Forest plot of pancreatitis prevalence in fatty pancreas**

**Supp. Figure 23 Forest plot of pancreatitis prevalence in the population without fatty pancreas**

**Supp. Figure 24 Forest plot of the risk of pancreatitis in patients with fatty pancreas**

**Supp. Figure 25 Sensitivity analysis of the risk of pancreatitis in patients with fatty pancreas**

**Supp. Figure 26 Forest plot of pancreatic cancer in fatty pancreas**

**Supp. Figure 27 Forest plot of pancreatic cancer prevalence in the population without fatty pancreas**

**Supp. Figure 28 Forest plot of the risk of pancreatic cancer in patients with fatty pancreas**

**Supp. Figure 29 Sensitivity analysis of the risk of pancreatic cancer in patients with fatty pancreas**

**Supp. Figure 30 Forest plot of pancreatic adenocarcinoma in fatty pancreas**

**Supp. Figure 31 Forest plot of pancreatic adenocarcinoma prevalence in the population without fatty pancreas**

**Supp. Figure** **32 Forest plot of the risk of pancreatic adenocarcinoma in patients with fatty pancreas**

**Supp. Figure 33 Sensitivity analysis of the risk of pancreatic adenocarcinoma in patients with fatty pancreas**

**Supp. Figure 34 Forest plot of fatty liver prevalence in fatty pancreas**

**Supp. Figure 35 Forest plot of fatty liver prevalence in the population without fatty pancreas**

**Supp. Figure 36 Forest plot of the risk of fatty liver in patients with fatty pancreas**

**Supp. Figure 37 Sensitivity analysis of the risk of fatty liver in patients with fatty pancreas**

**Supp. Figure 38 Forest plot of nonalcoholic fatty liver disease prevalence in fatty pancreas**

**Supp. Figure 39 Forest plot of nonalcoholic fatty liver disease prevalence in the population without fatty pancreas**

**Supp. Figure 40 Forest plot of the risk of nonalcoholic fatty liver disease in patients with fatty pancreas**

**Supp. Figure 41 Sensitivity analysis of the risk of nonalcoholic fatty liver disease in patients with fatty pancreas**

**Supp. Figure 42 Forest plot of metabolic syndrome prevalence in fatty pancreas**

**Supp. Figure 43 Forest plot of metabolic syndrome prevalence in the population without fatty pancreas**

**Supp. Figure 44 Forest plot of the risk of metabolic syndrome in patients with fatty pancreas**

**Supp. Figure 45 Sensitivity analysis of the risk of metabolic syndrome in patients with fatty pancreas**

**Supp. Figure 46 Forest plot of overweight prevalence in fatty pancreas**

**Supp. Figure 47 Forest plot of overweight prevalence in the population without fatty pancreas**

**Supp. Figure 48 Forest plot of the risk of overweight in patients with fatty pancreas**

**Supp. Figure 49 Sensitivity analysis of the risk of overweight in patients with fatty pancreas**

**Supp. Figure 50 Forest plot of obesity prevalence in fatty pancreas**

**Supp. Figure 51 Forest plot of obesity prevalence in the population without fatty pancreas**

**Supp. Figure 52 Forest plot of the risk of obesity in patients with fatty pancreas**

**Supp. Figure 53** **Sensitivity analysis of the risk of obesity in patients with fatty pancreas**

**Supp. Figure 54 Forest plot of central obesity prevalence in fatty pancreas**

**Supp. Figure 55 Forest plot of central obesity prevalence in the population without fatty pancreas**

**Supp. Figure 56 Forest plot of the risk of central obesity in patients with fatty pancreas**

**Supp. Figure 57 Sensitivity analysis of the risk of central obesity in patients with fatty pancreas**

**Supp. Figure 58 Forest plot of diabetes prevalence in fatty pancreas**

**Supp. Figure 59 Forest plot of diabetes prevalence in the population without fatty pancreas**

**Supp. Figure 60 Forest plot of the risk of diabetes in patients with fatty pancreas**

**Supp. Figure 61 Sensitivity analysis of the risk of diabetes in patients with fatty pancreas**

**Supp. Figure 62 Forest plot of type 2 diabetes prevalence in fatty pancreas**

**Supp. Figure 63** **Forest plot of type 2 diabetes prevalence in the population without fatty pancreas**

**Supp. Figure 64 Forest plot of the risk of type 2 diabetes in patients with fatty pancreas**

**Supp. Figure 65 Sensitivity analysis of the risk of type 2 diabetes in patients with fatty pancreas**

**Supp. Figure 66 Forest plot of insulin resistance prevalence in fatty pancreas**

**Supp. Figure 67 Forest plot of insulin resistance prevalence in the population without fatty pancreas**

**Supp. Figure 68 Forest plot of the risk of insulin resistance in patients with fatty pancreas**

**Supp. Figure 69 Sensitivity analysis of the risk of insulin resistance in patients with fatty pancreas**

**Supp. Figure 70 Forest plot of hypertension in fatty pancreas**

**Supp. Figure 71 Forest plot of hypertension in the population without fatty pancreas**

**Supp. Figure 72 Forest plot of the risk of hypertension in patients with fatty pancreas**

**Supp. Figure 73 Sensitivity analysis of the risk of hypertension in patients with fatty pancreas**

**Supp. Figure 74 Forest plot of hyperlipidemia in fatty pancreas**

**Supp. Figure 75 Forest plot of hyperlipidemia in the population without fatty pancreas**

**Supp. Figure 76 Forest plot of the risk of hyperlipidemia in patients with fatty pancreas**

**Supp. Figure 77 Sensitivity analysis of the risk of hyperlipidemia in patients with fatty pancreas**
